# Insights into the Mechanism of Carbon Dioxide and Propylene Oxide Ring-Opening Copolymerization Using a Co(III)/K(I) Heterodinuclear Catalyst

**DOI:** 10.1021/jacs.2c06921

**Published:** 2022-09-21

**Authors:** Arron C. Deacy, Andreas Phanopoulos, Wouter Lindeboom, Antoine Buchard, Charlotte K. Williams

**Affiliations:** † Department of Chemistry, Chemistry Research Laboratory, University of Oxford, Oxford OX1 3TA, U.K.; ‡ Department of Chemistry, Molecular Sciences Research Hub, Imperial College London, 82 Wood Lane, Shepherds Bush, London W12 OBZ, U.K.; § Department of Chemistry, Centre for Sustainable and Circular Technologies, University of Bath, Bath BA2 7AY, U.K.

## Abstract

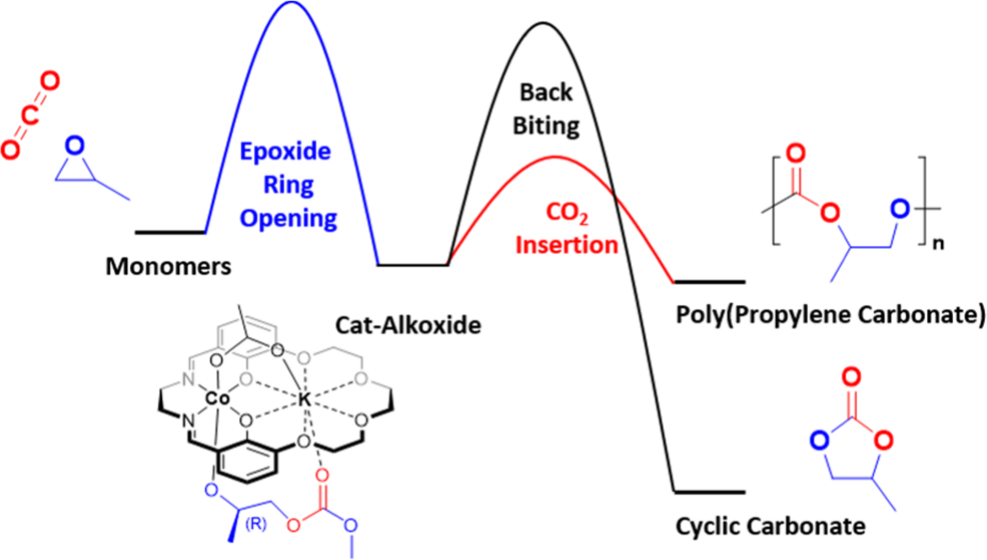

A combined computational and experimental investigation into the catalytic cycle of carbon dioxide and propylene oxide ring-opening copolymerization is presented using a Co(III)K(I) heterodinuclear complex (DeacyA. C.Co(III)/Alkali-Metal(I) Heterodinuclear Catalysts for the Ring-Opening Copolymerization of CO_2_ and Propylene Oxide. J. Am. Chem. Soc.2020, 142( (45), ), 19150−191603310873610.1021/jacs.0c07980PMC7662907). The complex is a rare example of a dinuclear catalyst, which is active for the copolymerization of CO_2_ and propylene oxide, a large-scale commercial product. Understanding the mechanisms for both product and byproduct formation is essential for rational catalyst improvements, but there are very few other mechanistic studies using these monomers. The investigation suggests that cobalt serves both to activate propylene oxide and to stabilize the catalytic intermediates, while potassium provides a transient carbonate nucleophile that ring-opens the activated propylene oxide. Density functional theory (DFT) calculations indicate that reverse roles for the metals have inaccessibly high energy barriers and are unlikely to occur under experimental conditions. The rate-determining step is calculated as the ring opening of the propylene oxide (Δ*G*_calc_^†^ = +22.2 kcal mol^–1^); consistent with experimental measurements (Δ*G*_exp_^†^ = +22.1 kcal mol^–1^, 50 °C). The calculated barrier to the selectivity limiting step, i.e., backbiting from the alkoxide intermediate to form propylene carbonate (Δ*G*_calc_^†^ = +21.4 kcal mol^–1^), is competitive with the barrier to epoxide ring opening (Δ*G*_calc_^†^ = +22.2 kcal mol^–1^) implicating an equilibrium between alkoxide and carbonate intermediates. This idea is tested experimentally and is controlled by carbon dioxide pressure or temperature to moderate selectivity. The catalytic mechanism, supported by theoretical and experimental investigations, should help to guide future catalyst design and optimization.

## Introduction

Polyurethanes (PUs) are produced on a 24 M tons per year scale and are widely applied, e.g., in automotive, electronics, clothing, construction, and consumer goods sectors.^1^ A key ingredient in making PUs are the polyols that are chain extended with diisocyanates. Currently, the most widely applied are short-chain polyethers (<5 kg mol^–1^), e.g., poly(ethylene oxide) and poly(propylene oxide). In recent years, carbon dioxide and propylene oxide (PO) ring-opening copolymerization (ROCOP) has furnished poly(propylene carbonate) (PPC) polyols which can also be used to form PU, showing high strength to weight ratios, high chemical-, UV- and hydrolytic resistance, and optical clarity.^1^ The copolymerization of propylene oxide and carbon dioxide significantly reduces greenhouse gas emissions compared with polypropylene oxide polyols. Life cycle assessments (cradle-to-gate) suggest for every CO_2_ molecule used, two more are saved by reducing propylene oxide usage.^2^ The same carbon dioxide/propylene oxide polymerization catalysis can be modified to yield high molar mass poly(propylene carbonate), which is a biodegradable plastic or solid-state electrolyte.^2^

The ring-opening copolymerization is a true carbon dioxide utilization process, furnishing polymers which are 44 wt % carbon dioxide derived. Catalysis has also proved effective in using captured carbon dioxide.^3^ The future for CO_2_-derived polymer production and application requires improvements and a better understanding of catalysis. Currently, heterogeneous double metal cyanides show outstanding rates but may be challenged by rather low carbon dioxide uptake and polycarbonate selectivity.^4^ Homogeneous catalysts can show high rates and high carbon dioxide uptake but have variable selectivity.^9−11^ They may also allow for insights into the catalytic mechanism through structure–activity investigations. This work sets out to investigate the mechanism for the copolymerization and its dominant side reaction, cyclic carbonate formation, using a recently reported dinuclear Co(III)K(I) catalyst.^8^

**Figure 1:**
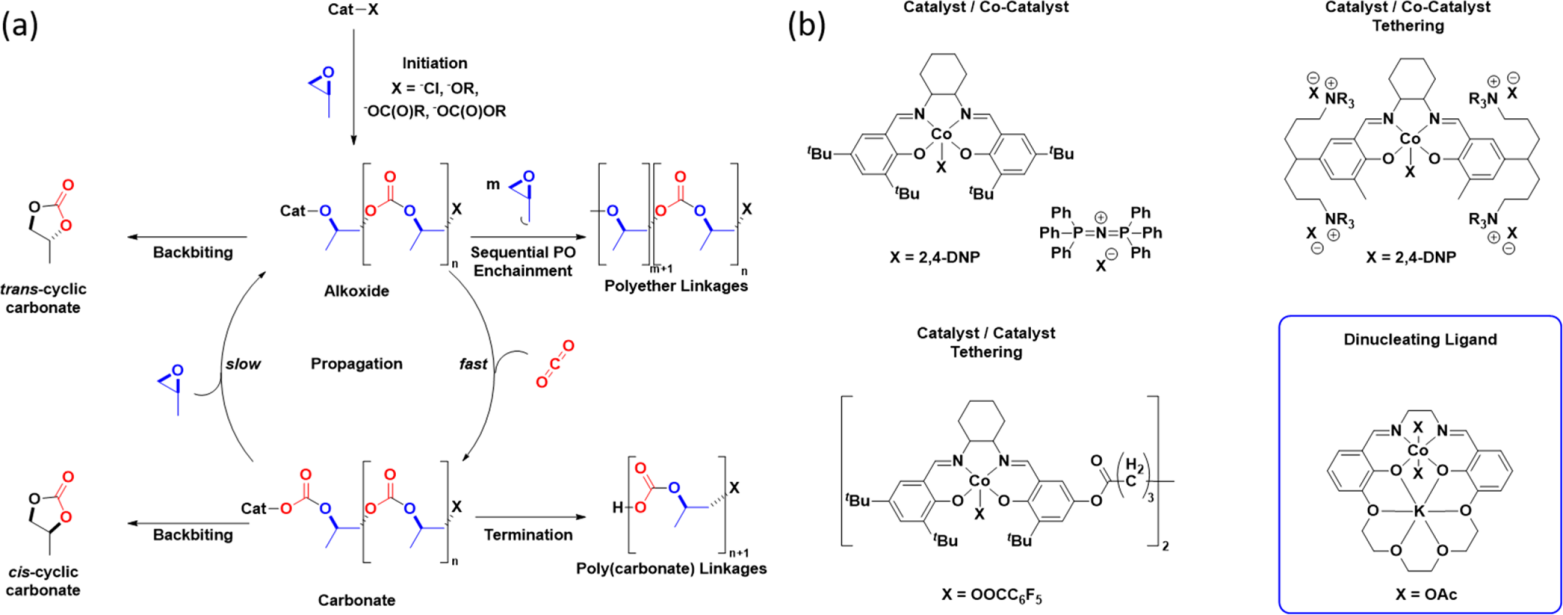
(a) Illustration of the reaction mechanism for the ring-opening copolymerization of carbon dioxide with propylene oxide along with the anticipated side reactions (cyclic carbonate and polyether formation). (b) Selection of cobalt salen catalysts and different design strategies applied to improve catalysis rates and selectivity.^5−8^

The elementary steps occurring during CO_2_/PO ring-opening copolymerization catalysis are empirically understood as (Figure 1a):

(1) Initiation involves the activation of an epoxide by coordination to a Lewis acid center followed by ring opening from an initiating group provided by the cocatalyst and/or coligand. The initiator is often a halide, carboxylate, or alkoxide species.
(2) Carbon dioxide insertion occurs with a rapid reaction of CO_2_ into an activated catalyst–alkoxide bond. In the cases where kinetic analyses are conducted, this step is usually not rate determining, although there are some exceptions.
(3) Propylene oxide ring opening involves its preactivation via coordination to a Lewis acid center (metal or nonmetal) and its subsequent nucleophilic attack and ring opening by a labile carbonate group.
(4) Termination occurs after the addition of protic reagents, including water, and results in the formation of a hydroxyl end-capped polymer chain.

CO_2_/PO ROCOP catalysis can suffer from reaction selectivity challenges (Figure 1a). For heterogeneous catalysts, sequential epoxide ring opening results in the formation of (poly)ether linkages, which alter the polymer physical–chemical properties, for example, by reducing the glass transition temperature (*T*_g_). Most homogeneous catalysts do not form ether linkages with the Co(III)K(I) catalyst explored in this work showing high carbon dioxide uptake.^8^ Another concerning side reaction is polymer chain backbiting to form the 5-membered heterocycle propylene carbonate (PC). Backbiting reactions may occur from either alkoxide or carbonate intermediates, and PC cannot re-enter polymerization cycles.^11^ Propylene carbonate is the thermodynamic product of the reaction between CO_2_/PO, and thus its formation is favored at higher temperatures.^9,10^

The most widely studied homogeneous catalysts tend to be transition metal complexes used in conjunction with an ionic cocatalyst (Figure 1b).^9−11^ These include the widely studied (salen)Co(III)X (X = halide) catalyst system, pioneered for CO_2_/PO ROCOP by Coates and team in 2003 and showing TOF = 81 h^–1^ (25 °C, 0.2 mol %, 55 bar).^12^ Polymerization rates and selectivity were both increased using an ionic cocatalyst, the most active was bis(triphenylphosphine)iminium chloride (PPNCl), resulting in TOF = 520 h^–1^ (22 °C, 0.05 mol %, 14 bar).^5,13^ One excellent design strategy is to tether the cocatalyst to the salen ligand, first reported by Nozaki and team and really improving the resulting activity.^6,14,15^ Lee and co-workers reported the best catalyst to date, featuring four such tethered ammonium groups attached to the Co(III) salen structure achieving an impressive TOF = 26,000 h^–1^ (80 °C, 0.002 mol %, 20 bar).^6^ The same design principle was also successfully used to increase the performances of metal porphyrin and organo-borane catalyst systems.^16−18^ The only drawback is that it often necessitates multistep syntheses and complicates catalyst speciation and structure–activity studies.^19,20^

An alternative design invokes a dinuclear mechanism and applies di- or multimetals in the active site—in such catalysts, the cocatalysts are not required. For example, Nozaki and team reported a dimeric [(salen)Co(O_2_C(C_6_F_5_))]_2_ complex showing a TOF = 430 h^–1^ (40 °C, 0.03 mol %, 53 bar).^7^ Rieger and team demonstrated a similar effect using a dimeric[(salen)Cr(Cl)]_2_ but with lower activity TOF = 67 h^–1^ (60 °C, 0.05 mol %, 40 bar).^21^ More recently, Chen and team reported a trimetallic system, [(salen)Co(III)(DNP)]_3_ (DNP = 2,4-dinitrophenolate), with TOF = 1740 h^–1^ (60 °C, 0.017 mol %, 30 bar).^22^ Our team has investigated dinuclear catalysts coordinated by macrocyclic diphenolate ligands. These complexes show metal–metal distance of 3–5 Å, akin to those observed in the active sites of heterogeneous, Zn-glutarate PO/CO_2_ ROCOP catalysts.^23^ Some of these dinuclear catalysts have proven highly active for cyclohexene oxide (CHO)/CO_2_ ring-opening copolymerization. For example, a synergic Mg(II)Co(II) catalyst showed TOF = 12,000 h^–1^ (140 °C, 0.05 mol %, 20 bar).^24^ However, most of the reported dinuclear catalysts underperform in PO/CO_2_ ROCOP.

Recently, we reported a heterodinuclear Co(III)K(I) complex coordinated by a macrocyclic ligand featuring both Schiff base and crown ether binding pockets. This complex showed good activity for PO/CO_2_ ROCOP with TOF = 800 h^–1^ (0.025 mol %, 70 °C, 30 bar).^8^ Even in the first report, its activity exceeds that of many di-Co(III) catalysts, and it has the additional benefit of removing half the cobalt and replacing it with potassium, a cheaper, nontoxic, readily available alkali metal. The catalyst is synthesized in three synthetic steps in good yields (74%). Further, it was stable to excess chain-transfer agent (up to 250 equiv); such additives are important to make polyols (<5 kg mol^–1^).^8^ This new catalyst meets many of the requirements for carbon dioxide/PO ROCOP. As such, a better understanding of its mechanism and how to improve activity would be very useful. In this area of catalysis, there are surprisingly few prior mechanistic investigations and thus there are many open questions including: (1) What are the roles of the different metals in the catalytic cycle? (2) What are the relative reaction barriers to polymerization? (3) How can polymer selectivity, over cyclic carbonate, be maximized? and (4) Are there design implications for future dinuclear catalysts that can be uncovered?

## Results and Discussion

### Initiation

**Figure 2:**
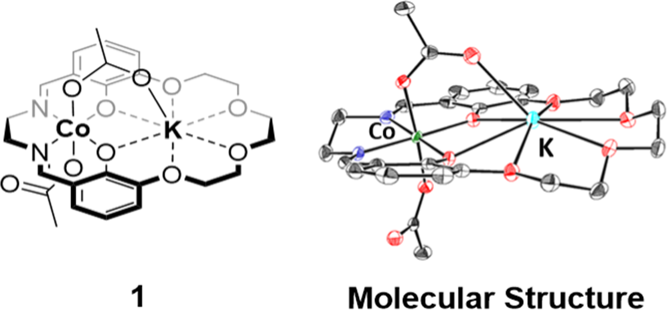
Illustration of the Co(III)K(I) heterodinuclear catalyst **1** alongside an ORTEP representation of the molecular structure of catalyst **1** obtained through single-crystal X-ray diffraction. Image adapted with permission from ref (8) Copyright 2020 American Chemical Society.

Initiation is the first monomer insertion into the catalyst structure, the speciation is dependent upon the initiating group. Where initiating groups are acetate, benzoate, or halide, propylene oxide ring opening is the first step, and it produces a new metal alkoxide intermediate. Where the initiators are alkyl, alkoxide, or phenoxide, carbon dioxide insertion is the first step to generate a metal carboxylate/carbonate intermediate. Since catalyst **1** features acetate coligands, propylene oxide ring opening should occur first (Figure 2). Density functional theory (DFT) calculations of the initiation process were conducted with propylene oxide coordination at the cobalt center, as opposed to potassium, as this coordination geometry results in significantly lower propagation barriers (*vide infra*). Given that the “Z”-shape “bottom” face shows the lowest energy barrier to PO ring opening (Figure S1, Table S3), this ligand conformation/complex geometry and positioning of PO were used for all subsequent calculations. As a side note, both *R*-PO and *S*-PO produced very similar transition state energy barriers, +21.1 and +22.4 kcal mol^–1^, respectively. The lack of stereoselectivity is unsurprising given the complex lacks chirality. For subsequent calculations, only *R*-PO was modeled (Table S3).

### Propagation

**Figure 3:**
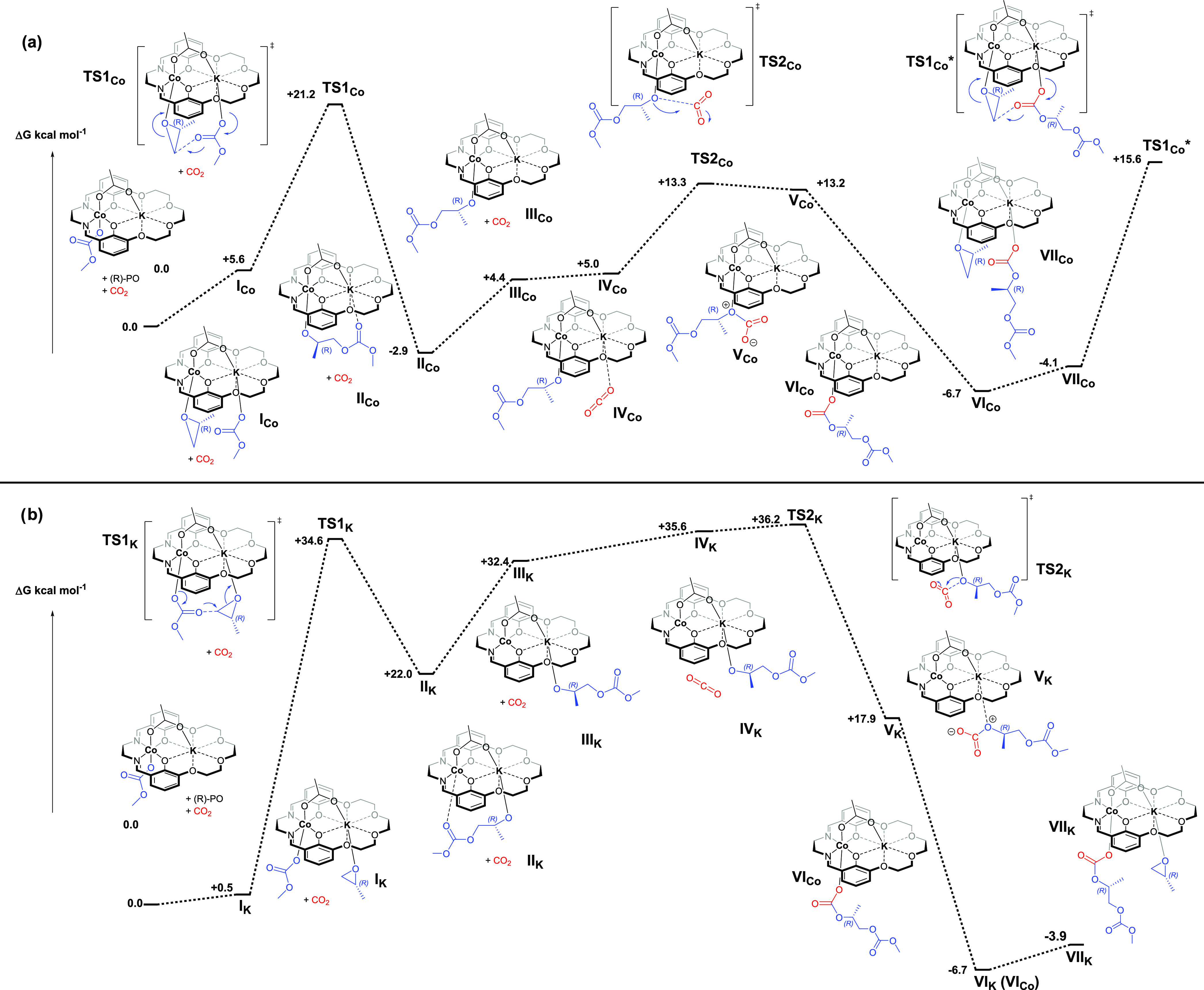
Illustrations of the potential energy surfaces for the alternating copolymerization of propylene oxide and carbon dioxide using the Co(III)K(I) catalyst **1**, where (a) propylene oxide coordination occurs at Co(III) and (b) propylene oxide coordination occurs at K(I).

The goals of modeling the propagation steps are to understand the roles of the two different metals and the reaction energy barriers and to identify the rate-limiting step. Two different dinuclear polymerization pathways were considered: one involving propylene oxide coordination at the Co(III) center (Figure 3a, Table S4) and the other with propylene oxide coordination at the K(I) center (Figure 3b, Table S5).

### Cobalt-Activated Epoxide

Propylene oxide coordination at Co(III) forms an intermediate (**I**_**Co**_) which is +5.6 kcal mol^–1^ higher in energy than the ground state catalyst structure. As the reactions are carried out in neat PO, its coordination is considered to be concentration favored. The ring opening of the PO, via nucleophilic attack from the potassium carbonate, has a transition state (**TS1**_**Co**_) energy of +21.2 kcal mol^–1^ and forms a stabilized cobalt–alkoxide intermediate (**II**_**Co**_) having an energy of −2.9 kcal mol^–1^. In the calculated structure of **II**_**Co**_, an adjacent polymer chain carbonate (or acetate during initiation) group coordinates to the potassium center, primarily via an electrostatic interaction (Figure S4), although very weak covalent bonding interactions are observed by natural bonding analysis (NBO) and quantum theory of atoms in molecules (QTAIM) calculations (Figure S5, Table S8). This is in line with previous reports of alkali metals playing an active role in carbon dioxide activation.^25,26^ A preorganization step precedes CO_2_ insertion and involves breaking of the potassium carbonate interaction, yielding a Co–alkoxide intermediate (**III**_**Co**_, +4.4 kcal mol^–1^) where the growing polymer chain has rotated 180° along the Co–O bond axis.

Subsequently, carbon dioxide coordination at potassium occurs by an end-on binding mode with a K–O_CO2_ distance of 3.13 Å (**IV**_**Co**_, +5.0 kcal mol^–1^). Carbon dioxide insertion involves a moderate energy transition state (**TS2**_**Co**_, +13.3 kcal mol^–1^) to form an isoenergetic zwitterionic intermediate (**V**_**Co**_, +13.2 kcal mol^–1^). In the last propagation step, the zwitterionic intermediate rearranges to form a stabilized Co–carbonate intermediate (**VI**_**Co**_, −6.7 kcal mol^–1^). Therefore, the Gibbs free energy to CO_2_ insertion is Δ*G*_calc_^†^ = +16.2 kcal mol^–1^ with respect to the alkoxide intermediate **II**_**Co**_. The next ring opening of PO has a transition state (**TS1**_Co_*) energy of +15.6 kcal mol^–1^ and a Gibbs free energy with respect to the carbonate intermediate **VI**_**Co**_ of Δ*G*_calc_^†^ = +22.2 kcal mol^–1^.

### Potassium-Activated Epoxide

The alternative pathway is where the propylene oxide is coordinated at K(I), and in the first step, this route results in only a small energy increase from the ground state catalyst structure (**I**_**K**_, +0.5 kcal mol^–1^). Notably, the potassium coordinated intermediate is +5.1 kcal mol^–1^ lower in energy than the respective cobalt coordinated epoxide (**I**_**Co**_), likely due to the greater number of accessible coordination sites and lack of geometric constraints at potassium. These are qualitatively visualized in a noncovalent interaction (NCI) plot, which shows a larger electrostatic attractive envelope around PO when coordinated to potassium in comparison to cobalt (Figure S2). Additionally, an attractive interaction between PO and the Co-bound carbonate is observed in **I**_k_, while Wiberg Bonding Index (WBI) analysis shows a stronger Co–O bond is preserved in **I**_k_ over **I**_Co_ (0.33 and 0.26, respectively). In the next step, the potassium-activated epoxide is ring-opened by the Co-carbonate nucleophile (**TS1**_**K**_, +34.6 kcal mol^–1^), forming a potassium–alkoxide intermediate. The intermediate is stabilized by the chelation of the polymer chain carbonate (or acetate) group to the adjacent cobalt center (**II**_**K**_, +22.0 kcal mol^–1^). The potassium–alkoxide intermediate (**II**_**K**_) is +24.9 kcal mol^–1^ higher in energy than the respective cobalt–alkoxide intermediate (**II**_**Co**_). The lower stability of **II**_**K**_ than **II**_**Co**_ is attributed to both the weaker potassium–oxygen bond (compared with the analogous cobalt–oxygen bond; WBI: 0.02 and 0.48, respectively) and to a weaker cobalt–carbonate chelation (compared to the analogous interaction with potassium, Figure S4). The insertion of carbon dioxide follows similar structural changes to those observed for the Co-activated epoxide mechanism. First, a preorganization of the polymer chain occurs to form a higher energy intermediate (**III**_**K**_, +32.4 kcal mol^–1^), then carbon dioxide enters the reactivity sphere but is not activated by the cobalt center with a Co–O_CO2_ distance measuring 4.35 Å (**IV**_**K**_, +35.6 kcal mol^–1^). This is followed by CO_2_ insertion into the potassium–alkoxide bond (**TS2**_**K**_, +36.2 kcal mol^–1^), forming a zwitterionic intermediate (**V**_**K**_, +17.9 kcal mol^–1^), and finally by rearrangement to form the cobalt–carbonate intermediate (**VI**_**K**_, −6.7 kcal mol^–1^; identical to **VI**_**Co**_ intermediate).

### Cobalt- vs Potassium-Activated Epoxide

Comparing the energy barriers for propylene oxide ring opening reveals that the cobalt-activated epoxide mechanism has significantly lower energy than the potassium-activated epoxide mechanism. The critical rate-limiting barriers are Δ*G*_calc_^†^ = +22.2 kcal mol^–1^ (Co-activated) and Δ*G*_calc_^†^ = +36.2 kcal mol^–1^ (K-activated), respectively. The difference between the barriers suggests that the potassium-activated pathway is very unlikely to occur experimentally, particularly given that typical conditions are 50 °C and 20 bar CO_2_. To substantiate this hypothesis, the propylene oxide ring-opening transition state barriers (**TS1**) were calculated for both pathways using a series of other appropriate functionals (ωB97X-D, B3LYP-D3BJ, PBE0-D3BJ, M06-GD3, and MN15). All functionals gave the same conclusion: the cobalt-activated epoxide mechanism (**TS1**_**Co**_, +16.2 to +21.2 kcal mol^–1^) resulted in a significantly lower energy barrier than the potassium-activated epoxide mechanism (**TS1**_**K**_, +29.2 to +34.6 kcal mol^–1^) (Table S6).

The cobalt-activated epoxide pathway shows a rate-determining step with the ring opening of the epoxide having an energy barrier of Δ*G*_calc_^†^ = +22.2 kcal mol^–1^. This step is clearly higher in energy compared with the insertion of CO_2_ (Δ*G*_calc_^†^ = +16.2 kcal mol^–1^). On the other hand, the potassium-activated epoxide mechanism shows a less clear rate-determining step, with both the ring opening of the epoxide and CO_2_ insertion having similar energy barriers, Δ*G*_calc_^†^ = +34.6 kcal mol^–1^ and +36.2 kcal mol^–1^, respectively. Further support for the Co-activated pathway comes when the experimental transition state energy barrier is compared with the calculated value.

The polymerization kinetics analysis already indicated that the rate-determining step is propylene oxide ring opening since the rate law was first order in epoxide and catalyst concentrations but zero order in CO_2_ pressure.

**Figure 4:**
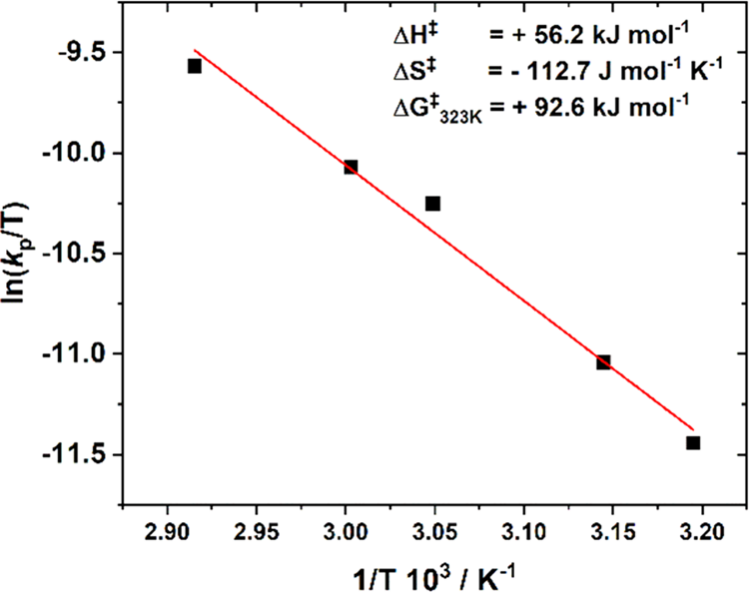
Eyring analysis for the transition state barrier to ring opening of propylene oxide during propagation using catalyst **1**. Image adapted with permission from ref (8) Copyright 2020 American Chemical Society.

The calculated epoxide ring-opening barrier (Δ*G*_calc_^†^ = +22.2 kcal mol^–1^) is closely comparable to the experimentally determined ring-opening barrier, Δ*G*_exp_^†^ = +22.1 kcal mol^–1^ (92.6 kJ mol^–1^) at 50 °C (Figure 4). Previously, a DFT investigation into the mechanism of cyclohexene oxide/carbon dioxide ROCOP using a di-zinc macrocyclic catalyst implicated a “chain-shuttling” mechanism. In that mechanism, the polymer chain migrates between the Zn(II) centers twice during each complete propagation cycle.^27^ The outcome is that one Zn(II) site is always coordinated to the alkoxide intermediate (after epoxide ring opening) and the other coordinates the carbonate intermediate (after carbon dioxide insertion). In contrast, for the Co(III)K(I) PO/CO_2_ ROCOP catalysis, the DFT investigation suggests that both the alkoxide and carbonate intermediates are coordinated to the cobalt center during propagation (**II**_**Co**_ and **VI**_**Co**_). The rate-determining step (**TS1**_**Co**_) involves propylene oxide activation at the cobalt center with the potassium center transiently stabilizing a polymer carbonate group. The Co-activated dinuclear pathway suggests that the role of cobalt is both to activate epoxide and to provide the labile alkoxide and carbonate nucleophiles. This mechanism suggests that future structure–activity relationship studies should focus on making changes to the Schiff base binding pocket coordinating to the Co(III) center. In the Co-activated epoxide dinuclear mechanism, the carbonate nucleophile is modeled as covalently bonded to potassium (WBI = 0.14, ρ(*r*) = 0.02, Table S8). Nonetheless, an alternative speciation where the carbonate anion is only associated with the cationic potassium center resulted in a transition state barrier that was only slightly higher (**TS1**_**Co**_**’**; +22.5 kcal mol^–1^). Given the similarity in the two barriers, it is experimentally credible that either K-coordinated or anionic carbonate nucleophiles are involved in the propagation mechanism. In the latter model, the role of potassium in the catalyst structure would be to stabilize the carbonate anion. Such an ionic coordination mode is reminiscent of the understanding of how catalyst/cocatalyst systems operate. Nonetheless, it is very important to emphasize that using potassium salts as separate additives to Co(III) salen catalysts results in nonselective polymerization catalysis (polymer selectivity = 41%, 0.05 mol %, 15 bar CO_2_, 25 °C).^28^ Thus, even if the role of potassium is as a stabilizing cation, it must be coordinated within the macrocyclic ligand for effective catalysis. Overall, the DFT calculations do not allow for unambiguous characterization of the role of potassium in the cycle, but it is clear that the Co-coordinated mechanism is more likely.

### Polymerization Selectivity Limits

The thermodynamic product of carbon dioxide/propylene oxide reaction is propylene carbonate, i.e., the 5-membered ring cyclic carbonate. During polymerizations, this cyclic carbonate can form by backbiting of either the alkoxide or carbonate intermediates. Darensbourg and team calculated anionic chain backbiting reactions and found barriers of 18.5 and 11.8 kcal mol^–1^ for carbonate and alkoxide anions, respectively.^29,30^ Metal coordinated intermediates (chain end groups) are less nucleophilic and thus expected to be less susceptible to backbiting reactions. Many researchers have attempted to stabilize the polymer chain ends to reduce backbiting reactions, particularly using metal-salen/cocatalyst systems. For example, Luinstra and Rieger calculated dissociation energies for Cr(III) and Al(III) salen catalyst systems, and their work suggested that Al(III) catalysts have lower polymer dissociation barriers and hence favor cyclic carbonate formation.^31^ Metal-salen catalysts always require cocatalysts (onium salts) to deliver the highest rates and selectivity for polycarbonate.^9−11^ These cocatalysts are proposed to stabilize the polymer chain end against backbiting. Metal-salen catalysts bearing tethered cocatalysts showed exceptional selectivity for polymer, even at elevated temperatures, but the detailed mechanisms for these systems are, so far, not reported.^6,14,15,19^

### Carbonate Backbiting

**Figure 5:**
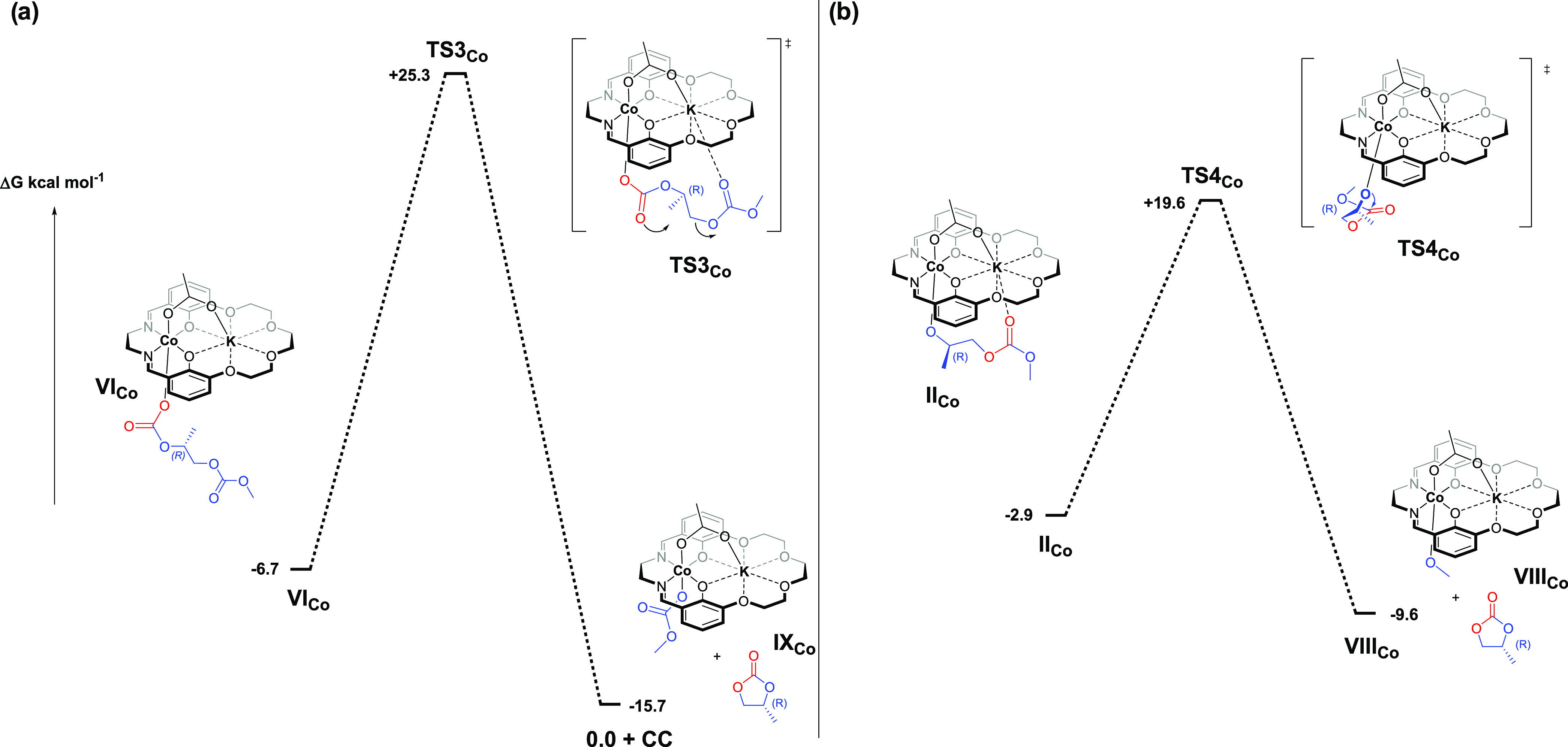
Illustration of the potential energy surfaces for the selectivity limiting step for reactions of CO_2_ with propylene oxide using Co(III)K(I) catalyst **1**. The selectivity limiting step involves either desirable copolymerization or undesirable backbiting to form propylene carbonate. Two pathways are examined for backbiting starting from either (a) the cobalt–carbonate intermediate **VI_Co_** or (b) the cobalt–alkoxide intermediate **II_Co_**.

DFT calculations of the two backbiting reactions were conducted using catalyst **1** (Figure 5, Table S7). The Co–carbonate intermediate reacts via a transition state showing coordination at the potassium of a neighboring carbonate group from the next unit in the polymer chain. Nucleophilic attack (S_N_2) of the cobalt–carbonate at the least hindered carbon–oxygen bond proceeds with a transition state barrier of +25.8 kcal mol^–1^ (**TS3**_**Co**_). This results in the formation of a chain-shortened Co–carbonate intermediate (**0.0**) together with an equivalent of propylene carbonate (Figure 5a). Cyclic carbonate is thermodynamically favored and thus has an energy of −15.7 kcal mol^–1^. This backbiting reaction has a Gibbs free energy of Δ*G*_calc_^†^ = +31.9 kcal mol^–1^ (against **VI**_**Co**_) and is +9.7 kcal mol^–1^ higher in energy than epoxide ring opening and, therefore, is unlikely to compete with propagation under the experimental polymerization conditions.

### Alkoxide Backbiting

An alternative route to propylene carbonate formation is through backbiting of the Co–alkoxide intermediate (**II**_**Co**_) (Figure 5b). To access the transition state, the polymer chain de-coordinates from the adjacent potassium center, with the chain rotating 90° along the Co–O bond axis. Nucleophilic attack occurs from the Co–alkoxide at the neighboring carbonyl carbon with a transition state energy of +19.6 kcal mol^–1^ (**TS4**_**Co**_). No subsequent TS was identified, and the transformation results in the reformation of a chain-shortened cobalt–alkoxide intermediate (**VIII**_**Co**_), along with one equivalent of propylene carbonate. The Gibbs free energy barrier of this backbiting reaction is Δ*G*_calc_^†^ = 22.4 kcal mol^–1^, which is competitive with the epoxide ring-opening barrier. Thus, the calculations suggest that the cobalt–alkoxide intermediate might undergo both copolymerization and backbiting under experimental conditions.

### Experimental Barrier to Propylene Carbonate Formation

It is essential to measure the rate of propylene carbonate formation to understand the product selectivity, but there are challenges to conducting such analyses during polymerizations. To consider how best to make the measurements, it is useful to consider the criteria for the determination of a reaction energy barrier:

(1) The barrier being measured must be rate-determining (pre- or post-transition state barriers must be lower in energy).
(2) The chemical structure of any model compound must resemble the energy minima prior to the rate-determining step.
(3) Competing side reactions must be minimized or removed (high product selectivity).

In this field, the barrier to backbiting (**TS4**) is almost always determined by the byproducts of CO_2_/PO ROCOP. However, such analyses must assume that the barrier to backbiting (**TS4**) is greater than the barrier to epoxide ring opening (**TS1**). Such an assumption may be acceptable for cyclohexene oxide/CO_2_ ROCOP since backbiting transitions through a strained bicyclic carbonate but is much less likely to be correct for propylene carbonate.

Indeed, here the barrier to Co–alkoxide backbiting was calculated as equivalent to epoxide ring opening (+22.4 vs +22.2 kcal mol^–1^), suggesting it cannot be determined appropriately during polymerization. Another detraction of using the polymerization side reactions to determine the barriers to cyclic carbonate formation is that cyclic carbonate is often the minor product, especially at low reaction temperatures. An alternative approach would be to determine the rate of propylene carbonate formation from the catalyzed depolymerization of poly(propylene carbonate). The PPC end groups are all hydroxyls since any carbonate chain ends rapidly decarboxylate upon carbon dioxide removal. These hydroxyl chain ends can react with the catalyst to generate a Co–alkoxide intermediate, which is structurally similar to (**II**_**Co**_). Since there is no epoxide ring-opening step (**TS1**) during depolymerization, the alkoxide intermediate backbiting will unequivocally be the rate-determining step. Measuring the rate of PPC backbiting also removes any competition from CO_2_ insertion (**TS2**), ensuring that all of the catalyst effects backbiting.

**Figure 6:**
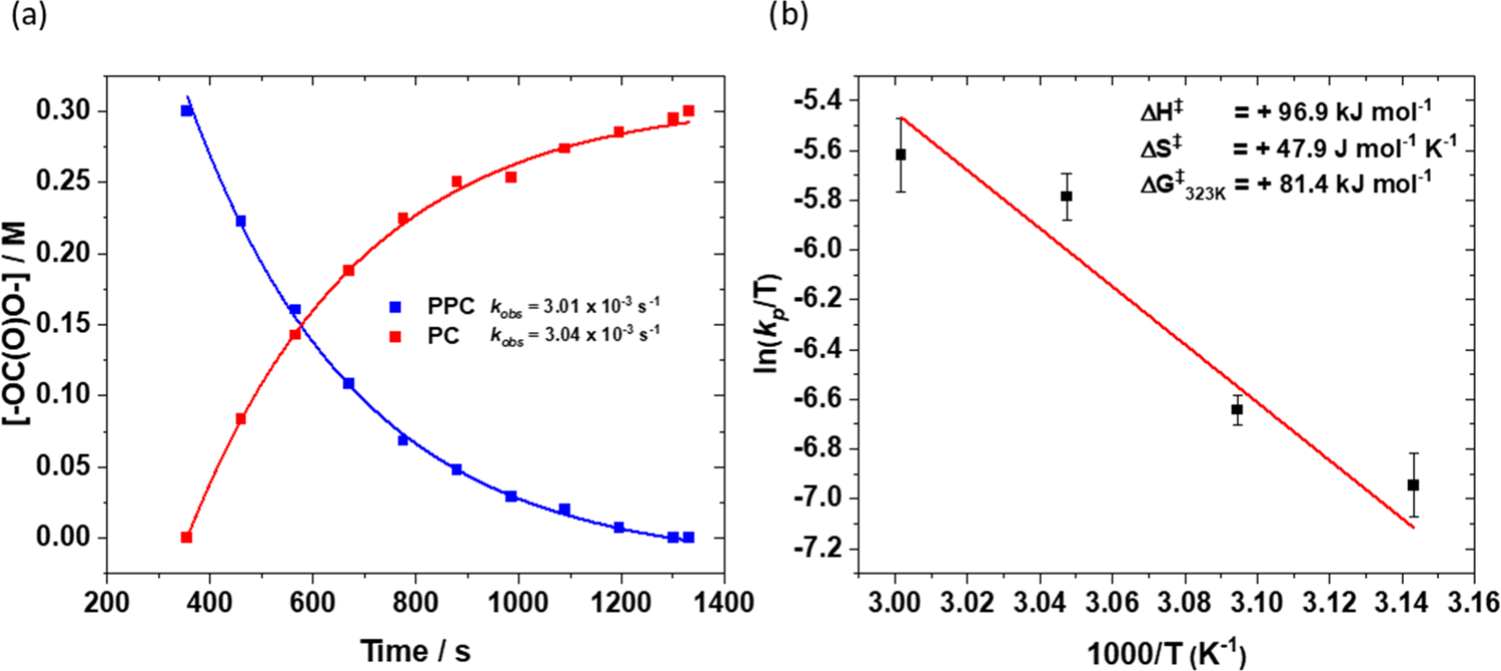
(a) Plot showing the change in concentration of carbonate linkages (−OC(O)O−) in both PPC and PC against time using catalyst **1**, at 55 °C, with an exponential fit to the data allowing for the determination of the pseudo first-order rate coefficient. (b) Eyring analysis, i.e., a plot of ln(*k*_b_/*T*) vs 1/*T*, for the decomposition of PPC into PC using catalyst **1**. Where *k*_b_ = *k*_obs_/[**1**] and *k*_obs_ is the gradient of the plot of ln[PPC]_t_/[PPC]_0_ vs time (s).

To experimentally determine the Co–alkoxide backbiting barrier, the rate of poly(propylene carbonate) (PPC) depolymerization to form propylene carbonate, using catalyst **1**, was monitored over the temperature range of 40–60 °C. The reaction was conducted by adding 3 mM catalyst to a 0.3 M solution of PPC in PO (6 mL), and *in situ* IR spectroscopy was used to interrogate changes in the intensity of absorptions assigned to PPC (1750 cm^–1^) and propylene carbonate, PC, (1800 cm^–1^) (Table S1). A dilute polymer solution was used to prevent any diffusion limitations to the rate of reaction and to ensure that the correct barrier was measured. An exponential decrease in PPC concentration with a concomitant increase in PC concentration was observed, indicating a 1^st^ order rate dependence (Figure 6a). Eyring analysis allowed for the determination of the free energy of backbiting as Δ*G*_323_^‡^ = +19.5 kcal mol^–1^ (where Δ*H*^‡^ = +24.8 kcal mol^–1^ Δ*S*^‡^ = −0.016 kcal mol^–1^ K^–1^), which is in line with the DFT calculations for propylene carbonate formation (**TS4**_**Co**_ = +22.4 kcal mol^–1^) (Figure 6b).

### Testing Polymerization Selectivity

**Figure 7:**
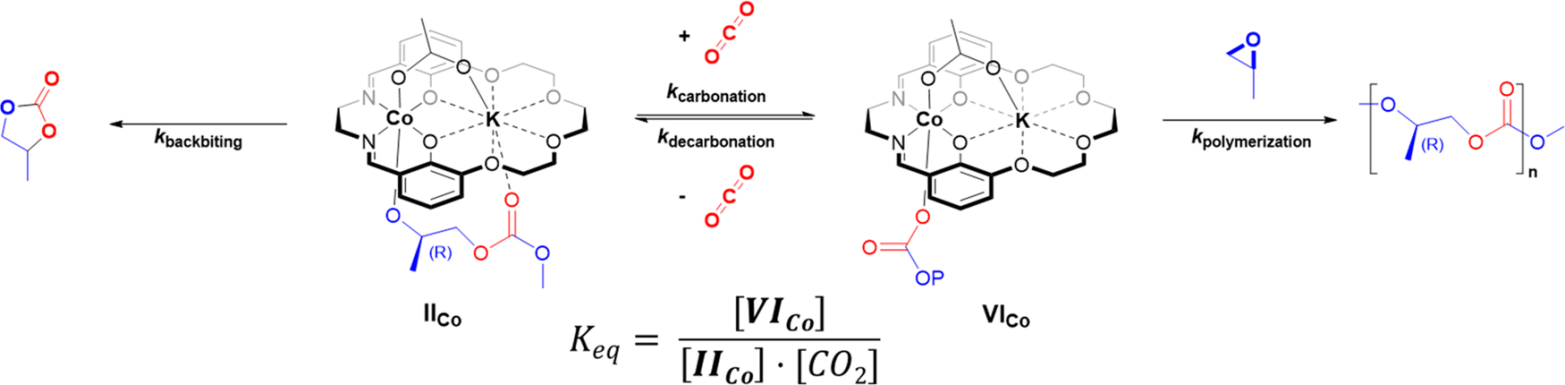
Illustration of the reaction equilibria for reversible CO_2_ insertion between intermediates **II_Co_** and **VI_Co_**.

The DFT calculations show that the barriers to carbonation (**II**_**C0**_ → **VI**_**Co**_) and decarbonation (**VI**_**Co**_ → **II**_**Co**_) reactions are accessible under the conditions of polymerization (16.2 and 20.0 kcal mol^–1^, respectively). The difference in free energy between the alkoxide and carbonate intermediates, **II**_**Co**_ and **VI**_**Co**_, is also small (Δ*G* ∼ 3.7 kcal mol^–1^), and the reaction system is sealed. These findings suggest the insertion of CO_2_ into the cobalt–alkoxide bond is an equilibrium (*K*_eq_, Figure 7). To test this notion, the experimental conditions were moderated and changes to the PPC vs PC selectivity were monitored. At 30 bar CO_2_ pressure and constant temperature (70 °C), the reaction showed high polymer selectivity (>90%). As the pressure was decreased from 30 to 6 bar, at the same constant temperature, the cyclic carbonate selectivity increased from 7 to 86% (Table 1).

**Table 1:** Pressure Dependence on the Polymer Selectivity for CO_2_/PO Reaction Using Catalyst **1**^a^

entry	CO_2_ (bar)	CO_2_ (molar)^b^	conv. (%)^c^	CO_2_ (%)^d^	polym. (%)^e^	cyclic (%)	TOF (h^–1^)^f^
1	6	0.6	11	>99	14	86	277
2	10	1.3	23	>99	46	54	667
3^8^	20	2.8	30	>99	63	33	833
4^8^	30	4.3	28	>99	93	7	834

a Reaction conditions: **1** (3 mM), PO (6 mL), 1,2-cyclohexane diol (60 mM), 70 °C, 1.4 h.

b Data supplied by ref (32).

c PO conversion determined from the relative integrals in the ^1^H NMR spectrum of the resonances assigned to PPC (4.92 ppm, 1H), PC (4.77 ppm, 1H), and PPO (3.46–3.64 ppm, 3H) against mesitylene (6.70 ppm).

d CO_2_ uptake (%) determined by the relative integrals in the ^1^H NMR spectrum of the resonances assigned to (PPC + PC)/PPO.

e Polym selectivity (%) determined by the relative integrals in the ^1^H NMR spectrum of the resonances assigned to PPC/(PC + PPC).

f Turn-over frequency (TOF) = TON/time (h).

These experimental observations are fully consistent with an insertion equilibrium since high pressures drive the equilibrium to the Co–carbonate intermediate, which does not undergo backbiting. Decreasing the carbon dioxide pressure results in a lower concentration of **VI**_**Co**_. The lower carbonate intermediate concentration reduces the rate of polymer propagation and increases the concentration of the alkoxide intermediate, **II**_**Co**_. Since backbiting reactions are feasible from the alkoxide intermediate, increasing its concentration increases the selectivity for (and rate of) cyclic carbonate formation. The overall rate of propylene oxide consumption decreases with decreasing pressure and may be due to the competitive coordination of propylene carbonate vs propylene oxide.

Next, a series of experiments changing the polymerization temperature were used to test the equilibrium hypothesis. The polymerization temperature was increased from 50 to 70 °C at constant (20 bar) CO_2_ pressure. At higher temperatures, the formation of cyclic carbonate was favored (7% at 50 °C vs 27% at 70 °C). This can also be rationalized as the insertion of CO_2_ into the cobalt–alkoxide bond is exothermic (Δ*H*_II–VI_ = −5.4 kcal mol^–1^), and an increase in temperature should decrease the equilibrium constant, *K*_eq_, thereby increasing the concentration of **II**_**Co**_. In addition, the concentration of CO_2_ dissolved in an epoxide, at a fixed pressure, decreases with increasing temperature, further driving the equilibrium in favor of **II**_**Co**_. Finally, considering the entropic factors, as Δ*S*^‡^ < 0 for polymerization, epoxide ring opening becomes less favored with increasing temperature. In contrast, as Δ*S*^‡^ > 0 for the backbiting reaction, the formation of cyclic carbonate becomes more favored with increasing temperature.

### Comparisons with Other PO/CO_2_ ROCOP Mechanisms

The Co(III)K(I) catalyst is a rare example of a dinuclear complex active using propylene oxide/carbon dioxide and operating without any cocatalyst. Regardless of the catalyst structure, there are very few other investigations into the PO/CO_2_ ROCOP mechanism, the majority of studies apply DFT calculations to investigate specific steps such as epoxide binding,^33^ CO_2_ insertion,^34−37^ chain dissociation,^31^ or backbiting reactions,^38^ independent of the complete cycle. One rationale for these “simplified” investigations is that the presence of the cocatalyst complicates the active site speciation.

CHO is significantly more reactive than PO due to its greater ring strain, and it often shows higher rates of polymerization. Further, the formation of bicyclic carbonate (cyclohexene carbonate) has a high barrier to formation, thereby increasing selectivity in CHO/CO_2_ ROCOP and enabling polymerizations at higher temperatures (>100 °C are typical). Catalysts such as the Mg(II)Co(II) catalyst showed activities >12,000 h^–1^ for CO_2_/CHO ROCOP (20 bar CO_2_), but an activity of just 5 h^–1^ and a polymer selectivity of 2% when using propylene oxide.^24^ Rieger and team reported a dinuclear “tethered” Zn-β-diimine catalyst showing an outstanding activity in CHO/CO_2_ ROCOP (TOF = 155,000 h^–1^, 100 °C, 30 bar), but it showed <1% conversion when using propylene oxide,^39,40^ producing mostly polyether. The same group subsequently used DFT to investigate the differences in reactivity between CHO and PO using the di-Zn catalyst.^39^ It was suggested that the formation of a highly stable zinc alkoxide intermediate prohibited further reactivity. The insertion of carbon dioxide was proposed to become rate-determining, and both sequential propylene oxide insertion and backbiting to cyclic carbonate were proposed as competitive. The di-Zn(II) alkoxide intermediate was proposed to be stabilized by a very close intermetallic distance of 3.58 Å, a value that is significantly lower than for the other calculated catalyst structures (5–6 Å).

**Figure 8:**
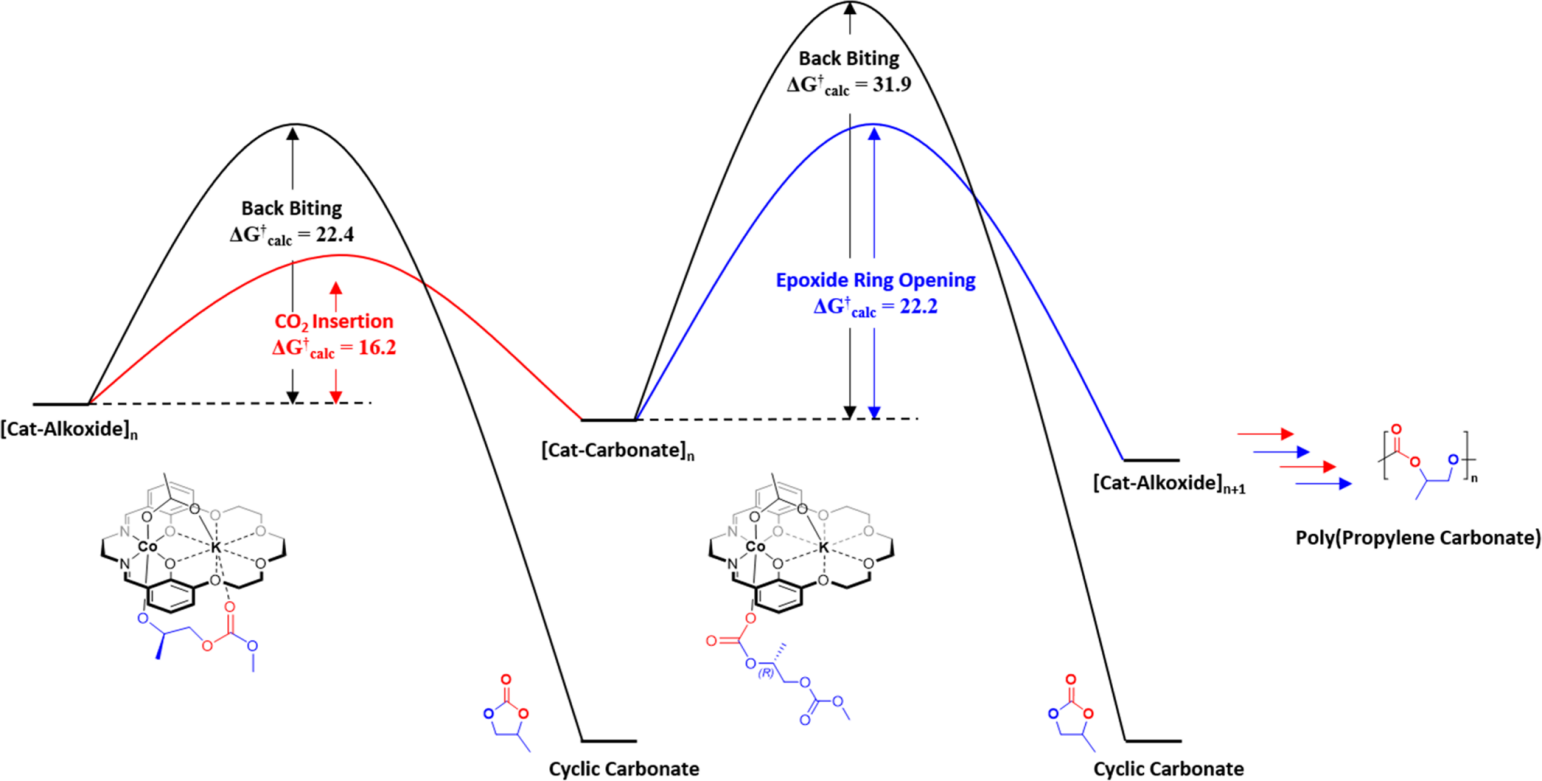
Illustration of the overall experimental and calculated barriers for the reactions of propylene oxide with carbon dioxide using catalyst **1**.

In this work, the Co–alkoxide intermediate is comparatively less stable and, thus, onward reactions to form poly(propylene carbonate) are feasible (Figure 8). The calculated metal–metal distances for intermediates **I**_**Co**_–**VI**_**Co**_ range between 3–4 Å, and thus short metal–metal distances are not in themselves a limitation to PO/CO_2_ ROCOP activity. Rather, it appears that the K(I) plays a pivotal role in enhancing the reactivity of the alkoxide intermediate, perhaps through its weaker chelation to the polymer chain compared with metals like Zn(II) or Mg(II), which have previously failed to effect forward reactions using PO/CO_2_. In our opinion, catalyst design has, to date, perhaps been “overly focussed” on attempting to reduce the epoxide ring-opening barrier rather than also considering the intermediate stability. In our view, when using propylene oxide, it is imperative to control the relative stability of the alkoxide intermediate and the CO_2_ insertion equilibrium. These aspects are likely to be controlled by both the catalyst structure and by the process conditions. In the best-case scenario, the transition state for epoxide ring opening has a low barrier, the catalyst–alkoxide intermediate is relatively destabilized compared with other intermediates, and the carbon dioxide insertion equilibrium favors the catalyst–carbonate intermediate.

## Conclusions

The copolymerization mechanism of carbon dioxide with propylene oxide using a heterodinuclear Co(III)K(I) complex was investigated by DFT calculations. The calculated transition state energy barriers were similar to experimental values for both polymerization and cyclic carbonate formation, providing support for the mechanism. The proposed mechanism involves a rate-determining step in which a cobalt-activated propylene oxide is attacked by a potassium-stabilized carbonate intermediate. The selectivity limiting step depends upon the equilibrium between the cobalt–alkoxide and cobalt–carbonate intermediates. The equilibrium can be externally manipulated, for example, by pressure or temperature, to favor the carbonate intermediate and increase the polymer selectivity.

Thus, the optimum conditions for polymerization involve reaction temperatures from 50–70 °C and carbon dioxide pressures 20–30 bar. These conditions are fully consistent with the proposed mechanism since they reduce the concentration of the cobalt–alkoxide intermediate and reduce the rate of chain backbiting to form propylene carbonate. The combined mechanism, underpinned by both DFT and experimental measurements, allows for the design of new dinuclear catalysts. The mechanism is a rare complete catalytic cycle for carbon dioxide/propylene oxide ROCOP catalysis and thus should also be useful to others designing metal-based or organocatalysts for carbon dioxide utilization.

## Supporting Information Available

DFT calculations; structures of all species; and experimental data for PPC degradation (PDF)
