# Supplementary material for: Insights into the Mechanism of Carbon Dioxide and Propylene Oxide Ring-Opening Copolymerization Using a Co(III)/K(I) Heterodinuclear Catalyst
Source: J Am Chem Soc. 2022 Sep 21;144(39):17929–38. doi: 10.1021/jacs.2c06921 (PMC9545154; doi:10.1021/jacs.2c06921)
Supplement: Supplementary file 1 — ja2c06921_si_001.pdf [file ja2c06921_si_001.pdf]

Supporting Information

Insights into the Mechanism of Carbon Dioxide and Propylene Oxide Ring Opening Copolymerization using a Co(III)/K(I) Heterodinuclear Catalyst.

Arron C. Deacy,[†] Andreas Phanopoulos,[†] Wouter Lindeboom, Antoine Buchard and Charlotte K. Williams*

Experimental 3

Table S1: Depolymerization of PPC using Catalyst **1** under standard conditions.[a] 3

a) Reaction Conditions: [cat] : [PPC] = 1 : 100 where [PPC] = 0.3 M in toluene. All reactions were run to completion as determined by in-situ IR monitoring. All reactions resulted in the depolymerization of PPC to PC where no polyether linkages were detected by ^{1}H NMR spectroscopy. 4

Density Functional Theory Calculations 4

Figure S1: a) Illustration of the Co(III)K(I) heterodinuclear catalyst **1**. b) illustration of the 'bowl'-shape conformation of the ligand resulting in 'concave' and 'convex' faces. c) ORTEP representation of the molecular structure of catalyst **1** obtained through single crystal X-ray diffraction. d) Illustration of the 'Z'-shape conformation of the ligand resulting in complexes with 'top' (κ_2-acetate) and 'bottom' (κ_1-acetate) faces. 5

Table S2: Computed Free Gibbs Energies of the bowl and Z-shape conformations of **1** (without and with temperature and concentration corrections).[a] 5

Table S3: Computed Free Gibbs Energies of the transition states for the first ring-opening of propylene oxide by **1** (**TS1$_{Co}$**; propylene oxide coordination at the cobalt center; without and with temperature and concentration corrections).[a] 6

Table S4: Computed Free Gibbs Energies of intermediates and transition states identified for the propagation step of the alternating copolymerization of R-propylene oxide (PO) and carbon dioxide using catalyst **1**, where PO coordination occurs at Co(III) (without and with temperature and concentration corrections). The starting point of the calculations is structure **1_prop_Z**, in which the ligand adopts a Z-shape conformation and one acetate ligand of **1** is replaced by one methyl carbonate ligand, modelling a growing polymer chain.[a] 6

Table S5: Computed Free Gibbs Energies of intermediates and transition states identified for the propagation step of the alternating copolymerization of R-propylene oxide (PO) and carbon dioxide using catalyst **1**, where PO coordination occurs at K(I) (without and with temperature and concentration corrections). The starting point of the calculations is structure **1_prop_Z**, in which the ligand adopts a Z-shape conformation and one acetate ligand of **1** is replaced by one methyl carbonate ligand, modelling a growing polymer chain.[a] 7

Table S6: Computed Free Gibbs Energies of **TS1$_{Co}$** and **TS1$_K$** transition states using various DFT functionals. (without and with temperature and concentration corrections).[a] 8

Table S7: Computed Free Gibbs Energies of intermediates and transition states identified for the carbonate and alkoxide back-biting reactions.[a] 9

Figure S2. Comparison of NCI plots of I_{Co} and I_{K}, as well as Wiberg Bonding Indices of key metal–oxygen bonds from NBO analysis 10

Figure S3. Comparison of NCI plots of $TS1_{Co}$ and $TS1_{K}$, as well as Wiberg Bonding Indices of key metal–oxygen bonds from NBO analysis 10

Figure S4. Comparison of NCI plots of II_{Co} and II_{K}, as well as Wiberg Bonding Indices of key metal–oxygen bonds from NBO analysis 11

Figure S5. Comparison of selected bond critical points (bcp) calculated for I_{Co} and I_{K} from QTAIM. ρ(r) and $\nabla^2\rho(r)$ in parentheses given in a.u. Data in include bcp for each primary metal–oxygen interaction as well as any between the (R)-PO and methylcarbonate fragments. 11

Figure S6. Comparison of selected bond critical points (bcp) calculated for $TS1_{Co}$ and $TS1_{K}$ from QTAIM. ρ(r) and $\nabla^2\rho(r)$ in parentheses given in a.u. Data in include bcp for each primary metal–oxygen interaction as well as any between the (R)-PO and methylcarbonate fragments. 12

Figure S7. Comparison of selected bond critical points (bcp) calculated for II_{Co} and II_{K} from QTAIM. ρ(r) and $\nabla^2\rho(r)$ in parentheses given in a.u. Data in include bcp for each primary metal–oxygen interaction as well as any between the (R)-PO and methylcarbonate fragments. 12

Table S8. NPA charges, Wiberg Bond Indices and selected QTAIM data (ρ(r) and $\nabla^2\rho(r)$ in parentheses from QTAIM given in a. u.) for $\mathbf{I_{Co}}$, $\mathbf{TS1_{Co}}$, $\mathbf{II_{Co}}$, $\mathbf{I_{K}}$, $\mathbf{TS1_{K}}$ and $\mathbf{II_{K}}$........ 13

Coordinates 14

References 53

Experimental

General Experimental

All experimental manipulations were performed using a dual-manifold nitrogen-vacuum Schlenk line or in a nitrogen filled glovebox. All solvents and reagents were obtained from commercial sources and used as received, unless stated otherwise. Propylene Oxide was dried over CaH_2 overnight and fractionally distilled prior to use. CP grade carbon dioxide (BOC, 99.995 %) was dried by passing it through two drying columns (VICI Metronics carbon dioxide purifier) in series, at 50 bar pressure, before use at lower pressures in the copolymerization. Catalyst **1** was synthesised according to literature procedure.[1]

Polymerization and depolymerization reactions were followed using a 100 mL Parr reactor fitted with a DiComp sentinel probe for in-situ-IR spectroscopic measurements.

General polymerization procedure: A solution of catalyst (13 mg, 0.02 mmol), *trans*-1,2-cyclohexene diol (46 mg, 0.4 mmol) and mesitylene (30 µL, 0.2 mmol) in PO (6 mL, 85.7 mmol) was injected into a 100 mL Parr Reactor, under a stream of dry carbon dioxide. The reactor was pressurized to the target reaction pressure and allowed to reach the required temperature. Upon reaction completion, the reactor vessel was cooled to room temperature, depressurized, and the reaction quenched via the addition of a 1 M solution of benzoic acid in $CHCl_3$.

General depolymerization procedure: A 100 mL Parr reactor vessel was charged with catalyst **1** (11.3 mg, 0.018 mmol) as a solid, followed by a solution of PPC (0.184 g, 1.8 mmol) in PO (6 mL, 85.7 mmol) and heated to the target temperature. Upon completion, the reactor vessel was cooled to room temperature and a sample of the crude reaction mixture was removed for NMR analysis.

Table S1: *Depolymerization of PPC using Catalyst **1** under standard conditions.*[a]

Entry	T (°C)	k_{obs} (s^{-1})	k_p ($dm^3\ mol^{-1}\ s^{-1}$)	$\ln(k_p/T)$	Average $\ln(k_p/T)$	Standard Deviation
1	45	0.93	0.31	-6.94	-6.95	0.127
2	45	0.77	0.26	-7.13		
3	45	1.02	0.34	-6.84		
4	45	0.98	0.33	-6.88		
5	50	1.36	0.45	-6.57	-6.64	0.058
6	50	1.19	0.40	-6.71		
7	50	1.27	0.42	-6.64		
8	50	1.24	0.41	-6.66		
9	55	3.40	1.13	-5.67	-5.79	0.093
10	55	2.71	0.90	-5.89		
11	55	3.04	1.01	-5.78		
12	55	2.98	0.99	-5.80		
13	60	4.52	1.51	-5.40	-5.62	0.148
14	60	3.33	1.11	-5.70		
15	60	3.43	1.14	-5.68		
16	60	3.34	1.11	-5.70		

a) Reaction Conditions: [cat] : [PPC] = 1 : 100 where [PPC] = 0.3 M in toluene. All reactions were run to completion as determined by in-situ IR monitoring. All reactions resulted in the depolymerization of PPC to PC where no polyether linkages were detected by ^{1}H NMR spectroscopy.

Density Functional Theory Calculations

Computational Methods

DFT calculations were run using Gaussian 16 (Revision C.01).[2] The reaction free energies were calculated using the hybrid exchange-correlation ωB97X-D functional, which includes D2 dispersion corrections described by Grimme.[3,4] A functional screening was undertaken on key transition states for both pathways, with the surveyed functionals including B3LYP[5,6] with Grimme's D3 dispersion correction including Becke-Johnson damping (GD3BJ),[7,8] M06[9] with Grimme's D3 dispersion correction (GD3)[10] and PBE0[11,12] with GD3BJ.[7,8] NBO analysis was performed using NBO 6.0.[13] QTAIM analysis was conducted using the AIMAll package.[14] Data are presented from *very fine* mesh calculations. Standard cut-offs for plotting the data with AIMAll were used. Non-covalent interactions were analysed using the NCIPLOT 3.0 program.[15]

The split valence 6-31+g(d,p) basis sets were used for carbon and hydrogen. This lower basis set was chosen as these elements do not bind directly to either catalytic metal center, but extra diffuse functions were added to capture more mid- and long-range interactions, for instance with growing polymer chains. The triple-ζ 6-311+g(d) basis set was used for potassium and all heteroatoms. Cobalt centers were described with the Stuttgart SDD ECP and associated basis sets.

Geometry optimisation calculations were performed without symmetry constraints and using an "ultrafine" grid for numerical integration. All structures are optimized using the self-consistent reaction field (SCRF) approach with conductor-like polarisable continuum model (CPCM).[16] In polymerization reactions, the propylene oxide (PO) monomer also serves as the solvent but is not implemented as a standard solvent in Gaussian16. A CPCM model for PO was therefore implemented by using the built-in non-polar parameters of tetrahydrofuran, modified with the polar parameters of propylene oxide (ε=16, n^2=1.867) ([SCRF=(cpcm,solvent=tetrahydrofuran),read] and [eps=16/epsinf=1.867)].

Free enthalpies were corrected using Goodvibes software,[17] with Grimme's quasiharmonic approximation applied with a frequency cut-off value of 100.0 wavenumbers, a frequency scale factor = 1.0. To account for experimental conditions, a temperature of 323.15 K and concentrations of 14.1156 mol L^{-1} (for neat PO), 4.787 mol L^{-1} (for CO_2 dissolved in PO at 50 bar and 313.15 K)[18] and 0.003 mol L^{-1} (for any metal complex species) were also applied.

All intermediates and transition states were characterised by normal coordinate analysis revealing either precisely zero or one imaginary frequency, respectively. In the case of transition states, the imaginary frequency corresponds to the mode of the intended reaction step. Full coordinates for all the calculated stationary points are included as part of the supplementary information (.xyz).

Full coordinates for all structures, together with computed energies and vibrational frequency data, are available via the corresponding Gaussian 16 output files and calculation spreadsheet, stored in the open-access digital repository, DOI: 10.6084/m9.figshare.20026409.

Optimisation of catalyst 1 structure

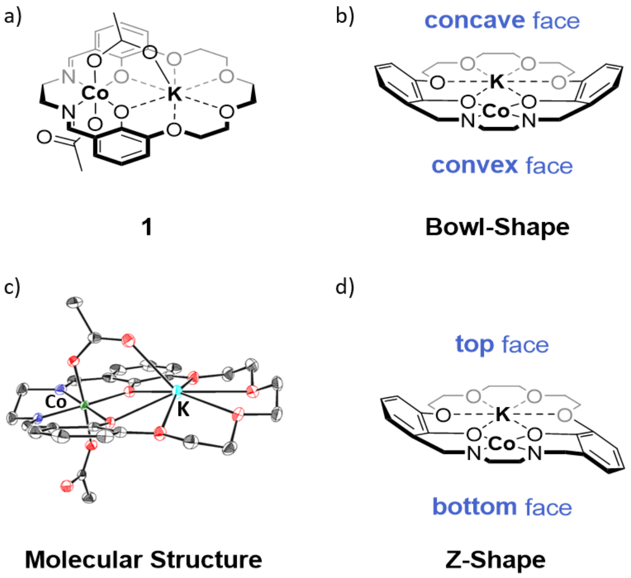

Figure S1: a) Illustration of the Co(III)K(I) heterodinuclear catalyst **1**. b) illustration of the 'bowl'-shape conformation of the ligand resulting in 'concave' and 'convex' faces. c) ORTEP representation of the molecular structure of catalyst **1** obtained through single crystal X-ray diffraction. d) Illustration of the 'Z'-shape conformation of the ligand resulting in complexes with 'top' (κ_2-acetate) and 'bottom' (κ_1-acetate) faces.

The molecular structure of Co(III)K(I), **1**, elucidated via single crystal X-ray diffraction, reveals the cobalt center is coordinated by the ligand's Schiff base binding cavity and the potassium is coordinated by the crown ether binding cavity. In the solid-state structure, the ligand adopts a Z-shape conformation and, because the acetate groups are both bridging and terminally coordinated, the catalyst has two different faces, labelled as 'top' and 'bottom' (Figure 2). The top face features the κ^2-acetate bridging between the cobalt and potassium, whereas the bottom face features the κ^1-acetate coordinated to cobalt. Previous investigations of similar macrocyclic complexes have revealed that the ligand can adopt either a 'Z'-shape or 'bowl'-shape and that often the latter is lower in energy.

When the ligand adopts the 'bowl'-shape, the complex has two different faces, labelled as 'concave' (ligand tilted towards the bridging acetate group) and 'convex' (ligand tilted away from the bridging acetate group). To understand which conformation would be most appropriate to use in subsequent DFT investigations, both 'Z'- and 'bowl'-shape conformations were subjected to geometry optimizations (Figure 2, Table S2). Both ground state geometries are energetically similar but the Z-shape is slightly lower in Gibbs free energy compared with the bowl shape (+4.8 kcal mol^{-1}).

DFT calculations of the initiation process were conducted with propylene oxide coordination at the cobalt center, as opposed to potassium, as this coordination geometry results in significantly lower propagation barriers (*vide infra*). The calculations were conducted using both the 'Z'- and 'bowl'-shape ligand conformations since their energy differences in the ground state were small (Table S3). For the 'Z'-shape ligand conformation, propylene oxide ring-opening barriers were +21.1 and +25.1 kcal mol^{-1} for the 'bottom' and 'top' faces, respectively. Whereas, the 'bowl' shape conformation, propylene oxide ring-opening on the catalyst's 'convex' face resulted in a transition state barrier of +25.9 kcal mol^{-1} (relative to 'Z'-shape ground state). The equivalent attack from the catalyst's 'concave' face could not be optimized due to the increased steric crowding and is much higher in energy.

Table S2*: Computed Free Gibbs Energies of the bowl and Z-shape conformations of* ***1*** *(without and with temperature and concentration corrections).*[a]

Structure	*G (Hartree)*	*ΔΔG (kcal mol^{-1})*	*G (Hartree)*[b]	*ΔΔG (kcal mol^{-1})*[b]
**1_Z**	-2615.850307	0.0	-2615.852097	0.0
**1_bowl**	-2615.842032	5.2	-2615.84444	4.8
**1_prop_Z**	-2691.070732	0.0	-2691.073021	0.0
**1_prop_bowl**	-2691.062276	5.3	-2691.06501	5.0

Calculations performed using rωb97xD functional, 6-31+g(d,p)/ 6-311+g(d)/SDD basis set and ECP, and modified cpcm solvation model (solvent=THF, ε=16, n^2=1.867). Default temperature = 298.15 K, concentration = 1.0 mol L^{-1}. 1_prop structures feature one methylcarbonate co-ligand in place of one acetate to model a propagating chain. [b]Goodvibes correction applied at 323.15 K and concentration = 0.003 mol L^{-1}.

Modelling of the initiation step of the copolymerisation

Table S3: *Computed Free Gibbs Energies of the transition states for the first ring-opening of propylene oxide by* ***1*** *(**TS1$_{Co}$**; propylene oxide coordination at the cobalt center; without and with temperature and concentration corrections).[a]*

Structure	*G (Hartree)*	*ΔΔG (kcal mol^{-1})*	*G (Hartree)[b]*	*ΔΔG (kcal mol^{-1})[b]*
**R-PO**	-193.025528		-193.02215	
**S-PO**	-193.025516		-193.022138	
**1_Z**	-2615.850307		-2615.852097	
**Reference for R-PO opening (1_Z + R-PO)**	-2808.875835	0.0	-2808.882901	0.0
**Reference for S-PO opening (1_Z +S-PO)**	-2808.875823	0.0	-2808.882889	
**Co_bowl_R**	-2808.831301	27.9	-2808.833052	25.9
**Co_bowl_S**	-2808.827754	30.2	-2808.830941	27.2
**Co_Z_bottom_R**	-2808.837199	24.2	-2808.840665	21.1
**Co_Z_bottom_S**	-2808.834532	25.9	-2808.838474	22.4
**Co_Z_top_R**	-2808.832643	27.1	-2808.834192	25.1
**Co_Z_top_S**	-2808.832655	27.1	-2808.834171	25.1

Calculations performed using rωb97xD functional, 6-31+g(d,p)/ 6-311+g(d)/SDD basis set and ECP, and modified cpcm solvation model (solvent=THF, ε=16, n^2=1.867). Default temperature = 298.15 K, concentration = 1.0 mol L^{-1}. [b]Goodvibes correction applied at 323.15 K and concentration = 0.003 mol L^{-1} (14.1156 mol L^{-1} for [PO]).

Modelling of the propagation step of the copolymerisation

Cobalt activated-epoxide pathway

Table S4: *Computed Free Gibbs Energies of intermediates and transition states identified for the propagation step of the alternating copolymerization of R-propylene oxide (PO) and carbon dioxide using catalyst* ***1****, where PO coordination occurs at Co(III) (without and with temperature and concentration corrections). The starting point of the calculations is structure* ***1_prop_Z****, in which the ligand adopts a Z-shape conformation and one acetate ligand of* ***1*** *is replaced by one methyl carbonate ligand, modelling a growing polymer chain.[a]*

Structure	G (Hartree)	ΔΔG (kcal mol^{-1})	G (Hartree)[b]	ΔΔG (kcal mol^{-1})[b]
**R-PO**	-193.025528		-193.02215	
**CO_2**	-188.583009		-188.580102	
**0.0 (1_prop_Z)**	2691.070732		-2691.073021	
**Reference (0.0 +R-PO)**	-2884.09626	0.0	-2884.095171	0.0
**Reference (0.0 +R-PO+CO_2)**	-3072.679269	0.0	-3072.675273	0.0
**Reference (0.0 +2 R-PO+CO_2)**	-3261.262278	0.0	-3265.697423	0.0
**I_{Co}**	-2884.082375	8.7	-2884.086202	5.6
**$TS1_{Co}$**	-2884.057402	24.4	-2884.06133	21.2
**$TS1_{Co}$'**	-2884.056148	25.2	-2884.059263	22.5
**II_{Co}**	-2884.096972	-0.4	-2884.099825	-2.9
**III_{Co}**	-2884.08681	5.9	-2884.088175	4.4
**IV_{Co}**	-3072.665935	8.4	-3072.667256	5.0
**$TS2_{Co}$**	-3072.652366	16.9	-3072.654037	13.3
**V_{Co}**	-3072.652354	16.9	-3072.654227	13.2
**VI_{Co}**	-3072.683798	-2.8	-3072.68588	-6.7
**VII_{Co}**	-3265.701869	1.8	-3265.70388	-4.1
**$TS3_{Co}$**	-3265.670705	21.4	-3265.67261	15.6

Calculations performed using rωb97xD functional, 6-31+g(d,p)/ 6-311+g(d)/SDD basis set and ECP, and modified cpcm solvation model (solvent=THF, ε=16, n^2=1.867). Default temperature = 298.15 K, concentration = 1.0 mol L^{-1}. [b]Goodvibes correction applied at 323.15 K and concentration = 0.003 mol L^{-1} (14.1156 mol L^{-1} for [PO]; 4.787 mol L^{-1} for [CO_2])..

Potassium activated-epoxide pathway

***Table S5**: Computed Free Gibbs Energies of intermediates and transition states identified for the propagation step of the alternating copolymerization of R-propylene oxide (PO) and carbon dioxide using catalyst **1**, where PO coordination occurs at K(I) (without and with temperature and concentration corrections). The starting point of the calculations is structure **1_prop_Z**, in which the ligand adopts a Z-shape conformation and one acetate ligand of **1** is replaced by one methyl carbonate ligand, modelling a growing polymer chain.[a]*

Structure	G (Hartree)	ΔΔG (kcal mol^{-1})	G (Hartree)[b]	ΔΔG (kcal mol^{-1})[b]
**R-PO**	-193.025528		-193.02215	
**CO_2**	-188.583009		-188.580102	
**0.0 (1_prop_Z)**	-2691.070732		-2691.073021	
**Reference (0.0 +R-PO)**	-2884.09626	0.0	-2884.095171	0.0
**Reference (0.0 +R-PO+CO_2)**	-3072.679269	0.0	-3072.675273	0.0
**Reference (0.0 +2 R-PO+CO_2)**	-3261.262278	0.0	-3265.697423	0.0
**I_K**	-2884.091051	3.3	-2884.094399	0.5
**$TS1_K$**	-2884.036085	37.8	-2884.040086	34.6
**II_K**	-2884.056033	25.2	-2884.060074	22.0
**III_K**	-2884.042542	33.7	-2884.043595	32.4
**IV_K**	-3072.618289	38.3	-3072.618592	35.6
**$TS2_K$**	-3072.616858	39.2	-3072.617581	36.2
**V_K**	-3072.645914	20.9	-3072.646795	17.9
**VI_K (VI_{Co})**	-3072.683798	-2.8	-3072.68588	-6.7
**VII_K**	-3265.702792	1.3	-3265.703589	-3.9

Calculations performed using rωb97xD functional, 6-31+g(d,p)/ 6-311+g(d)/SDD basis set and ECP, and modified cpcm solvation model (solvent=THF, ε=16, n^2=1.867). Default temperature = 298.15 K, concentration = 1.0 mol L^{-1}. [b]Goodvibes correction applied at 323.15 K and concentration = 0.003 mol L^{-1} (14.1156 mol L^{-1} for [PO]; 4.787 mol L^{-1} for [CO_2])..

Influence of DFT method (functional) on $TS1_{Co}$ and $TS1_K$ Free Gibbs Energies

***Table S6**: Computed Free Gibbs Energies of **$TS1_{Co}$** and **$TS1_K$** transition states using various DFT functionals. (without and with temperature and concentration corrections).[a]*

Functional	*Structure*	*G (Hartree)*	*ΔΔG (kcal mol^{-1})*	*G (Hartree)*[b]	*ΔΔG (kcal mol^{-1})*[b]
**rωb97xD**	**R-PO**	-193.025528		-193.02215	
	**1_prop_Z (0.0)**	-2691.070732		-2691.073021	
	**Reference**	-2884.09626	0.0	-2884.095171	0.0
	**$TS1_{Co}$**	-2884.057402	24.4	-2884.06133	21.2
	**$TS1_K$**	-2884.036085	37.8	-2884.040086	34.6
**B3LYP-D3BJ**	**R-PO**	-193.100182		-193.096811	
	**1_prop_Z (0.0)**	-2691.893649		-2691.896552	
	**Reference**	-2884.993831	0.0	-2884.993363	0.0
	**$TS1_{Co}$**	-2884.965592	17.7	-2884.968984	15.3
	**$TS1_K$**	-2884.943403	31.6	-2884.94682	29.2
**M06-D3**	**R-PO**	-192.955836		-192.952461	
	**1_prop_Z (0.0)**	-2690.474913		-2690.478616	
	**Reference**	-2883.430749	0.0	-2883.431077	0.0
	**$TS1_{Co}$**	-2883.40183	18.1	-2883.406006	15.7
	**$TS1_K$**	-2883.378609	32.7	-2883.383501	29.9
**MN15**	**R-PO**	-192.851937		-192.848563	
	**1_prop_Z (0.0)**	-2689.474129		-2689.477857	
	**Reference**	-2882.326066	0.0	-2882.32642	0.0
	**$TS1_{Co}$**	-2882.290791	22.1	-2882.295209	19.6
	**$TS1_K$**	-2882.268018	36.4	-2882.272834	33.6
**PBE0-D3BJ**	**R-PO**	-192.867421		-192.864043	
	**1_prop_Z (0.0)**	-2689.364354		-2689.367298	
	**Reference**	-2882.231775	0.0	-2882.231341	0.0
	**$TS1_{Co}$**	-2882.196923	21.9	-2882.200613	19.3
	**$TS1_K$**	-2882.173904	36.3	-2882.177451	33.8

Calculations performed using named functional, 6-31+g(d,p)/ 6-311+g(d)/SDD basis set and ECP, and modified cpcm solvation model (solvent=THF, ε=16, n^2=1.867). Default temperature = 298.15 K, concentration = 1.0 mol L^{-1}. [b]Goodvibes correction applied at 323.15 K and concentration = 0.003 mol L^{-1} (14.1156 mol L^{-1} for [PO]).

Back-biting reaction pathways

***Table S7**: Computed Free Gibbs Energies of intermediates and transition states identified for the carbonate and alkoxide back-biting reactions.*[a]

Structure	G (Hartree)	ΔΔG (kcal mol^{-1})	G (Hartree)[b]	ΔΔG (kcal mol^{-1})[b]
**R-PO**	-193.025528		-193.02215	
**CO_2**	-188.583009		-188.580102	
**0.0 (1_prop_Z)**	-2615.850307		-2691.073021	
**R-propylene carbonate**	-381.62166	-8.2		
**Carbonate back-biting pathway**				
**Reference (0.0 +R-PO+CO_2)**	-3072.679269	0.0	-3072.675273	0.0
**VI_{Co}**	-3072.683798	-2.8	-3072.68588	-6.7
**$TS3_{Co}$**	-3072.631852	29.8	-3072.63502	25.3
**IX_{Co}**	-3072.673336	3.7	-3072.67687	-1.0
**0.0 + R-propylene carbonate**	-3072.692392	-8.2	-3072.70025	-15.7
**Alkoxide back-biting pathway**				
**Reference (0.0 +R-PO)**	-2884.09626	0.0	-2884.095171	0.0
**II_{Co}**	-2884.096972	-0.4	-2884.099825	-2.9
**$TS4_{Co}$**	-2884.060373	22.5	-2884.063982	19.6
**X_{Co}**	-2884.096295	0.0	-2884.098289	-2.0
**VIII_{Co}**	-2502.479331		-2502.483277	
**VIII_{Co} + R-propylene carbonate**	-2884.100991	-3.0	-2884.110506	-9.6

Calculations performed using named functional, 6-31+g(d,p)/ 6-311+g(d)/SDD basis set and ECP, and modified cpcm solvation model (solvent=THF, ε=16, n^2=1.867). Default temperature = 298.15 K, concentration = 1.0 mol L^{-1}. [b]Goodvibes correction applied at 323.15 K and concentration = 0.003 mol L^{-1} (14.1156 mol L^{-1} for [PO]).

IX_{Co}

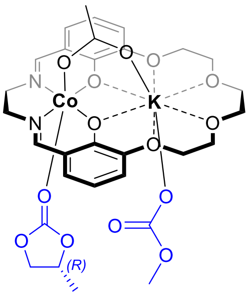

X_{Co}

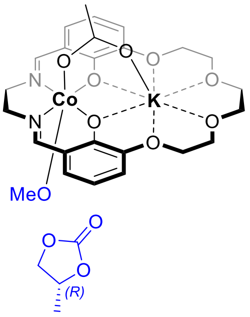

VIII_{Co}

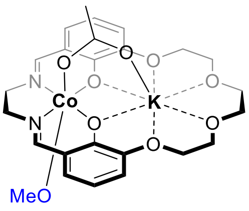

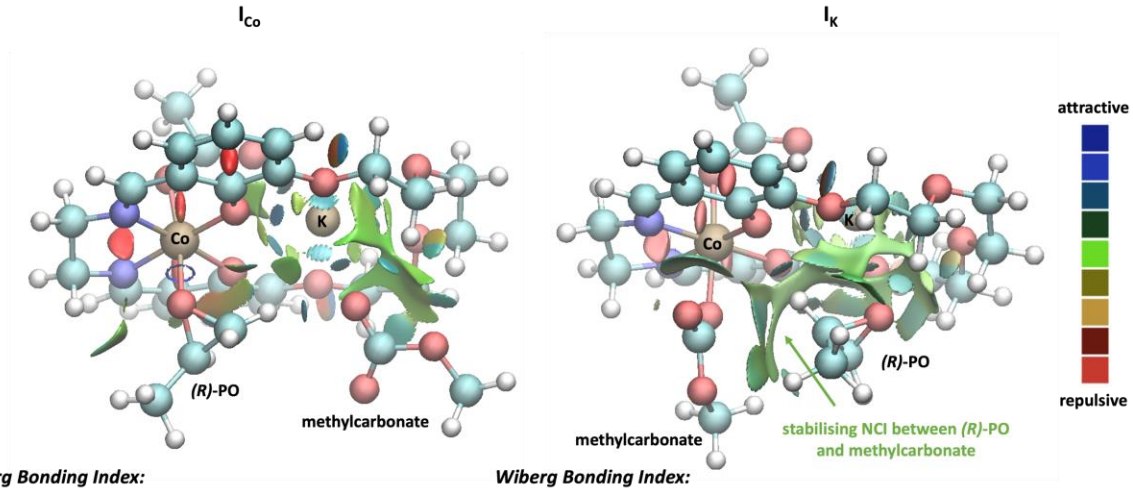

Wiberg Bonding Index:
Co–O: 0.26
K–O: 0.14

Wiberg Bonding Index:
Co–O: 0.33
K–O: 0.11

Figure S2. Comparison of NCI plots of I_{Co} and I_K, as well as Wiberg Bonding Indices of key metal–oxygen bonds from NBO analysis

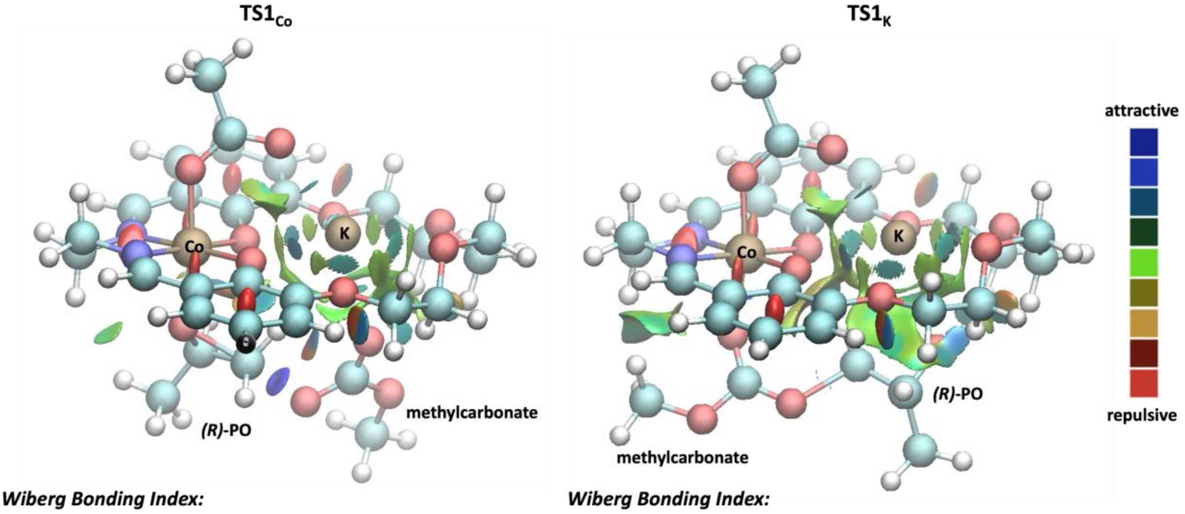

Wiberg Bonding Index:
Co–O: 0.36
K–O: 0.13

Wiberg Bonding Index:
Co–O: 0.23
K–O: 0.12

Figure S3*. Comparison of NCI plots of $TS1_{Co}$ and $TS1_K$, as well as Wiberg Bonding Indices of key metal–oxygen bonds from NBO analysis*

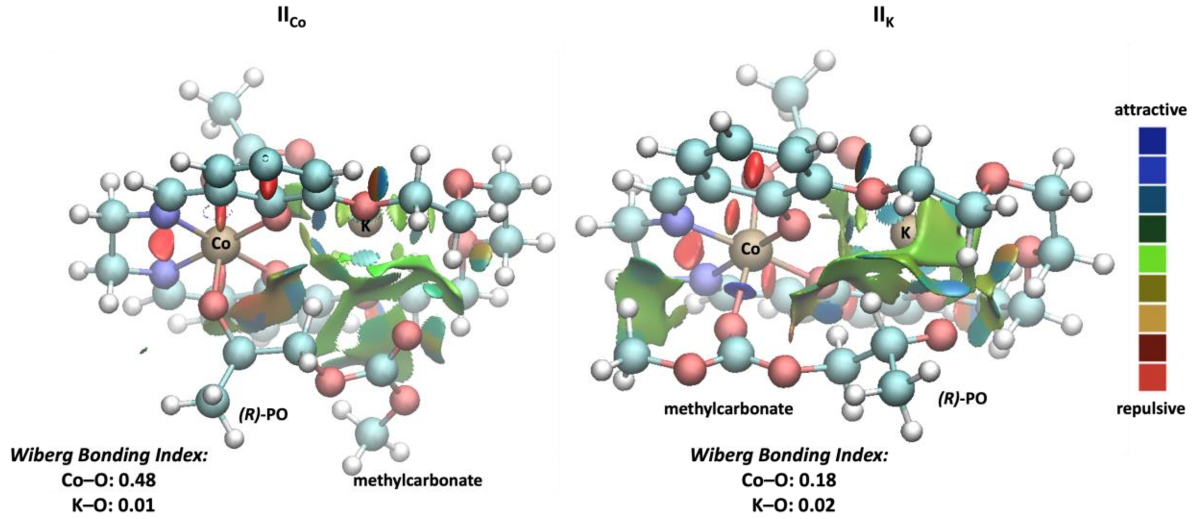

Figure S4. Comparison of NCI plots of II_{Co} and II_K, as well as Wiberg Bonding Indices of key metal–oxygen bonds from NBO analysis

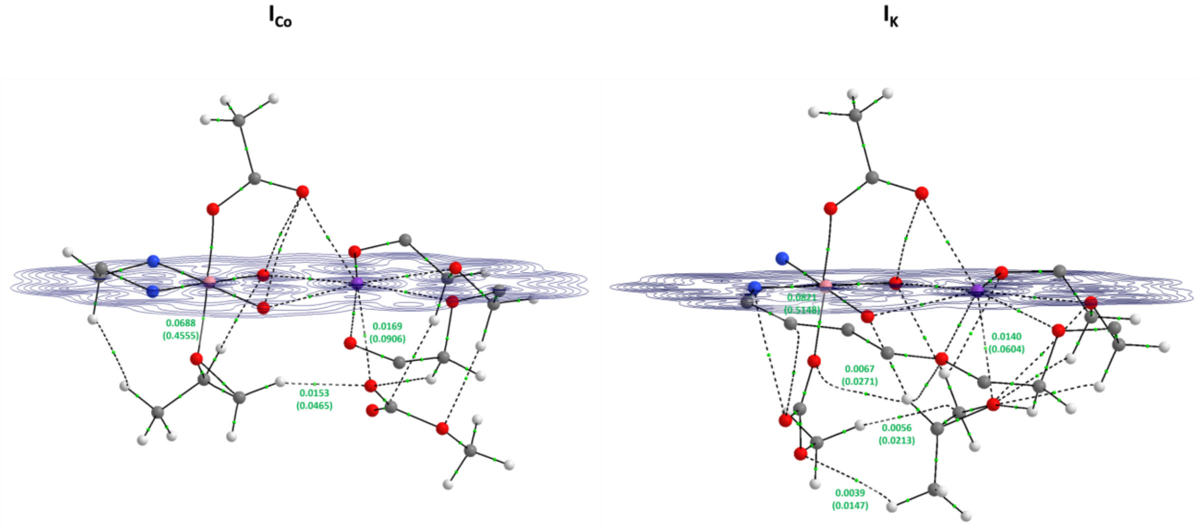

Figure S5. *Comparison of selected bond critical points (bcp) calculated for I_{Co} and I_K from QTAIM. $\rho(r)$ and $\nabla^2\rho(r)$ in parentheses given in a.u. Data in include bcp for each primary metal–oxygen interaction as well as any between the (R)-PO and methylcarbonate fragments.*

$TS1_{Co}$ **$TS1_K$**

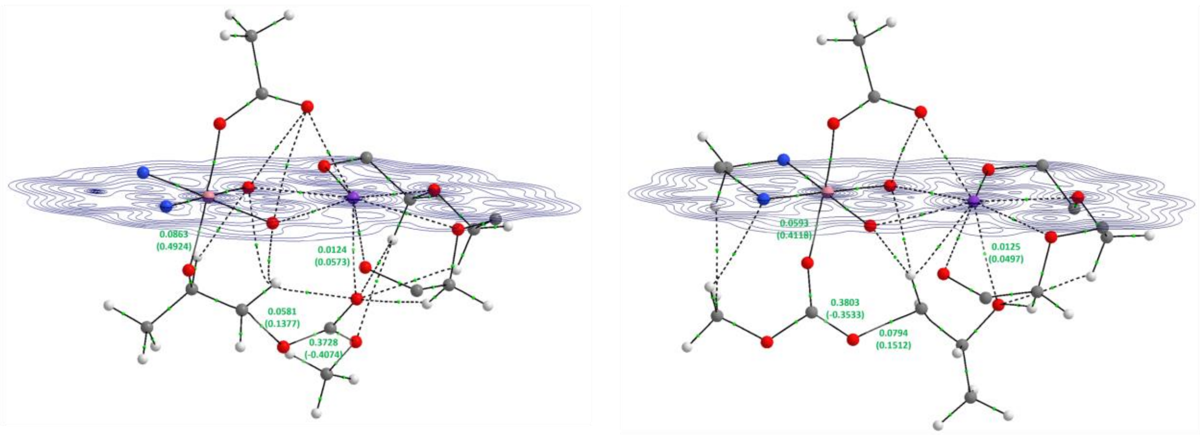

***Figure S6**. Comparison of selected bond critical points (bcp) calculated for $TS1_{Co}$ and $TS1_K$ from QTAIM. $\rho(r)$ and $\nabla^2\rho(r)$ in parentheses given in a.u. Data in include bcp for each primary metal–oxygen interaction as well as any between the (R)-PO and methylcarbonate fragments.*

II_{Co} **II_K**

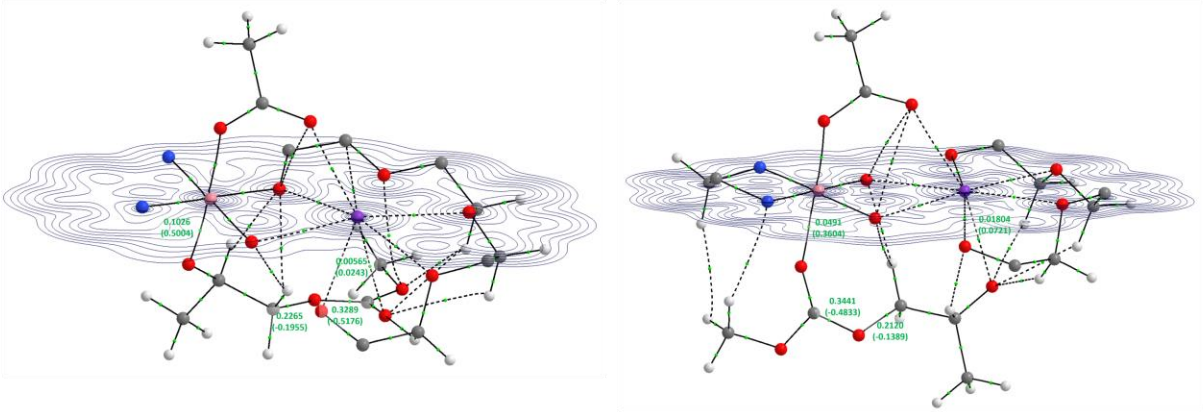

Figure S7. Comparison of selected bond critical points (bcp) calculated for II_{Co} and II_K from QTAIM. $\rho(r)$ and $\nabla^2\rho(r)$ in parentheses given in a.u. Data in include bcp for each primary metal–oxygen interaction as well as any between the (R)-PO and methylcarbonate fragments.

Table S8. *NPA charges, Wiberg Bond Indices and selected QTAIM data ($\rho(r)$ and $\nabla^2\rho(r)$ in parentheses from QTAIM given in a. u.) for* $\mathbf{I_{Co}}$, $\mathbf{TS1_{Co}}$, $\mathbf{II_{Co}}$, $\mathbf{I_K}$, $\mathbf{TS1_K}$ *and* $\mathbf{II_K}$.

	$\mathbf{I_{Co}}$	$\mathbf{TS1_{Co}}$	$\mathbf{II_{Co}}$	$\mathbf{I_K}$	$\mathbf{TS1_K}$	$\mathbf{II_K}$
			NPA charges			
Co	1.32	1.31	1.31	1.34	1.35	1.35
K	0.90	0.91	0.91	0.91	0.91	0.90
			WBIs			
Co–O	0.26	0.36	0.48	0.33	0.23	0.18
K–O	0.14	0.13	0.01	0.11	0.12	0.02
PO–carbonate interaction(s)	0.02	0.01	-	0.0004<br>0.001	-	-
New PO–carbonate bond formed	-	0.28	0.83	-	0.37	0.80
			$\rho(r)$ and $\nabla^2\rho(r)$ in parentheses from QTAIM given in a. u.			
Co–O	0.07(0.46)	0.09(0.49)	0.10(0.50)	0.08(0.52)	0.06(0.41)	0.05(0.36)
K–O	0.02(0.09)	0.01(0.06)	0.01(0.02)	0.01(0.06)	0.01(0.05)	0.02(0.07)
PO–carbonate interaction(s)	0.02(0.05)	0.02(0.06)	-	0.01(0.03)<br>0.01(0.02)	-	-
New PO–carbonate bond formed	-	0.06(0.14)	0.23(-0.20)	-	0.08(0.15)	0.21(-0.14)

Coordinates

Ligand Conformational Screening

1_bowl.log

SCF (wB97xd) = -2616.32148935
E(SCF)+ZPE(0 K)= -2615.769255
H(298 K)= -2615.729767
G(298 K)= -2615.842032
Lowest Frequency = 17.1257cm-1

O	1.15602400	-0.02358400	0.41601700
C	2.41038900	0.08141700	0.13870000
C	3.20166900	-1.11969700	0.08567100
C	-3.50165500	-0.94016300	-0.52700300
C	-2.72476200	0.21498200	-0.17162100
O	-1.57070000	0.04951100	0.39498200
C	-3.30716300	1.47832100	-0.45998200
C	-2.63281800	2.70668300	-0.11956100
H	-3.21884600	3.62720800	-0.17820400
N	-1.41354100	2.79171200	0.24725200
C	-0.80379300	4.05017300	0.64449000
H	-0.71374300	4.05085000	1.73515100
H	-1.41248500	4.90591600	0.33816800
C	0.57252700	4.08189800	-0.00228600
N	1.15067700	2.74160500	0.12546000
C	2.40530900	2.56818500	-0.06536100
H	3.02148100	3.45603300	-0.23240100
C	3.08981900	1.30224800	-0.11144400
C	4.46787700	1.31449600	-0.43859400
H	4.95214200	2.26874500	-0.62834500
C	5.17997500	0.14382500	-0.53303400
C	4.53476000	-1.08329600	-0.26789100
H	5.10828900	-1.99970600	-0.34664500
C	-4.73515200	-0.81324000	-1.13926200
H	-5.30063500	-1.69408200	-1.41987400
C	-5.28622600	0.45345700	-1.41136900
C	-4.58063800	1.58085800	-1.06307400
H	-4.99067000	2.56855200	-1.25640500
O	-0.17386900	-0.84855100	-2.16213300
C	-0.25120400	0.35024300	-2.44082300
O	-0.21792000	1.33920800	-1.62022100
C	-0.43820900	0.78622700	-3.88767900
H	-0.18553400	-0.02687300	-4.56988600
H	-1.48920500	1.05654400	-4.03597400
H	0.16590600	1.66829200	-4.11168200
H	-6.25755700	0.52559800	-1.88798100
H	6.23005500	0.14902800	-0.80343900
Co	-0.17108700	1.36088000	0.30403800
K	-0.23532900	-2.21193400	0.17122400
H	0.47066500	4.30734300	-1.06940000
H	1.21655600	4.83596800	0.45857400
O	2.51749400	-2.25418600	0.41033800
O	-2.93053600	-2.14429400	-0.22358500
C	-3.52139100	-3.31095500	-0.77546300
H	-3.54359800	-3.23128500	-1.87006300
H	-4.54789400	-3.43448800	-0.40771100
C	-2.73578700	-4.52953600	-0.35313800
H	-3.26387500	-5.41264600	-0.73975400
H	-2.70564400	-4.60156400	0.74403600
C	3.16956300	-3.50796300	0.30072100
H	3.30876700	-3.76158600	-0.75853900
H	4.15446200	-3.48430700	0.78341100
C	2.33991700	-4.54316000	1.04167200
H	2.85380400	-5.51258500	0.97005400
H	2.28288400	-4.26089600	2.09617000
O	1.00169000	-4.65558700	0.60815600
O	-1.42092600	-4.46967800	-0.86828900
C	0.78804000	-5.35864700	-0.60246200
H	1.35373800	-6.30087300	-0.60460200
H	1.10821300	-4.76199400	-1.46852100
C	-0.68889700	-5.67225000	-0.69693800
H	-1.00017900	-6.17723400	0.22714300
H	-0.87424100	-6.34817400	-1.54253300
O	0.00208800	1.47942900	2.21333800
C	-0.89604700	1.61244000	3.12745600
O	-2.09374000	1.83938200	2.95787800
C	-0.32565900	1.46067800	4.53319600
H	-0.00011200	0.42613800	4.68093500
H	0.55150900	2.10112900	4.65838100
H	-1.07728200	1.71071400	5.28331000

1_prop_bowl.log

SCF (wB97xd) = -2691.54706719
E(SCF)+ZPE(0 K)= -2690.988975
H(298 K)= -2690.948806
G(298 K)= -2691.062276
Lowest Frequency = 17.3086cm-1

O	1.15602600	-0.01369500	0.37085400
C	2.41636400	0.09221100	0.12150300
C	3.20833700	-1.10880300	0.09245700
C	-3.50251200	-0.92509400	-0.51927500
C	-2.72809100	0.23117100	-0.16653900
O	-1.57267000	0.07143300	0.40347500
C	-3.31089000	1.49317600	-0.45640800
C	-2.63284300	2.72100600	-0.12047900
H	-3.21815400	3.64226500	-0.17194800
N	-1.40894300	2.80401600	0.23168300
C	-0.78965500	4.06229400	0.61563100
H	-0.68028700	4.06458900	1.70442200
H	-1.40209400	4.91834800	0.31819400
C	0.57413700	4.08737500	-0.05720600
N	1.15526900	2.74959700	0.07954400
C	2.41262900	2.57687600	-0.09462400
H	3.02903200	3.46474600	-0.25967000
C	3.09939000	1.31212400	-0.12098400
C	4.48402500	1.32374400	-0.41952900
H	4.97152700	2.27723800	-0.60446500
C	5.19821300	0.15291400	-0.49214100
C	4.54850600	-1.07325800	-0.23325700
H	5.12446800	-1.98942000	-0.29470200
C	-4.73669200	-0.79934900	-1.13145000
H	-5.30160000	-1.68081400	-1.41108500
C	-5.28887200	0.46620100	-1.40462200
C	-4.58473300	1.59519600	-1.05796500
H	-4.99544100	2.58229900	-1.25237900
O	-0.21820600	-0.85345900	-2.15320200
C	-0.28847100	0.34036000	-2.44825900
O	-0.24022300	1.34253300	-1.64085200

C	-0.48516300	0.76172600	-3.89745900
H	-0.24722100	-0.06232800	-4.57161800
H	-1.53473700	1.04096500	-4.03857800
H	0.12581500	1.63464100	-4.13780900
H	-6.26042500	0.53666900	-1.88099200
H	6.25375100	0.15718500	-0.74028200
Co	-0.16602500	1.37166200	0.26919900
K	-0.24219700	-2.19868400	0.20436100
H	0.45226200	4.29711400	-1.12541200
H	1.22583600	4.84811400	0.38111200
O	2.51754900	-2.24080000	0.41090400
O	-2.93155000	-2.12833000	-0.21399400
C	-3.51871300	-3.29543000	-0.76971700
H	-3.53763500	-3.21357000	-1.86417200
H	-4.54598200	-3.42139700	-0.40512200
C	-2.73261000	-4.51364700	-0.34729900
H	-3.25855900	-5.39653500	-0.73727300
H	-2.70576300	-4.58789400	0.74982200
C	3.17160700	-3.49555000	0.32415400
H	3.32389400	-3.76099400	-0.73031600
H	4.15032600	-3.46591200	0.81873100
C	2.33418700	-4.52294400	1.06740700
H	2.84675900	-5.49386900	1.00719200
H	2.27003500	-4.23223400	2.11919100
O	0.99890100	-4.63627500	0.62579000
O	-1.41628000	-4.45125200	-0.85838500
C	0.79247800	-5.33833200	-0.58664800
H	1.35970000	-6.27962700	-0.58718700
H	1.11582000	-4.73983700	-1.45021300
C	-0.68361700	-5.65370200	-0.68830200
H	-0.99807800	-6.16223400	0.23273300
H	-0.86471900	-6.32681400	-1.53701700
O	0.03154400	1.48207700	2.18565300
C	-0.84639100	1.60602400	3.10160700
O	-2.01772800	1.95340000	3.02178500
O	-0.38105100	1.32732800	4.35788500
C	0.96768300	0.89214500	4.49912000
H	1.66767900	1.65653300	4.15145000
H	1.11008500	0.71692100	5.56618600
H	1.14454400	-0.03286000	3.94356800

1_prop_Z.log

SCF (wB97xd) = -2691.55409192
E(SCF)+ZPE(0 K)= -2690.996443
H(298 K)= -2690.956061
G(298 K)= -2691.070732
Lowest Frequency = 8.8283cm-1

Co	2.48473900	6.37211900	23.17419900
K	5.77646300	7.50889100	22.08532700
O	1.63268800	6.10195600	21.47577300
O	3.15880300	8.05301100	22.61409600
O	4.02121700	5.56064400	22.38880100
O	4.65208100	10.06521100	21.94138500
O	3.26182500	6.46928100	24.93000500
O	5.83850100	5.08761100	20.60437700
O	8.02859100	6.81642600	20.49574400
O	7.18533700	9.58710800	20.80183600
O	5.31248700	7.36471300	24.67812600
O	0.43885200	7.97303500	21.04312300
N	1.75346500	4.68842100	23.70920900
N	0.93444400	7.12648400	23.97006700
C	4.05671900	4.35088000	21.93686000
C	5.07468800	4.02405700	20.98052200
C	2.84973200	9.18359200	23.15931200
C	3.66196300	10.32341200	22.84235300
C	1.02881200	6.94635100	20.73555700
C	0.80445800	8.34958400	24.31749500
H	-0.09830600	8.63663200	24.86259300
C	3.35286600	1.99518900	21.82726800
H	2.67398600	1.21384700	22.15847300
C	4.38127600	6.92663900	25.35869900
C	0.65818100	4.85368600	24.65733200
H	1.08870200	4.98857400	25.65433200
H	-0.00227800	3.98185300	24.66385100
C	1.51760800	10.67620200	24.59441200
H	0.66722000	10.80743100	25.25835500
C	5.22267400	2.73746000	20.50289000
H	6.00508100	2.50039000	19.79056400
C	3.18284900	3.30654200	22.33174900
C	2.34840200	11.73494700	24.31292600
H	2.17838400	12.70988900	24.75622700
C	2.10559200	3.54149500	23.26297000
H	1.54820500	2.66431300	23.60196600
C	5.50604600	11.12496800	21.54927600
H	6.13553700	11.43395000	22.39397700
H	4.91896500	11.98789000	21.20997800
C	1.75175100	9.39940500	24.03069700
C	3.42794300	11.55379600	23.42254900
H	4.07003100	12.39860300	23.19989500
C	6.90058200	4.87359700	19.69207400
H	6.53107100	4.40282500	18.77186200
H	7.66100700	4.22462300	20.14519400
C	4.35585600	1.70918300	20.93089500
H	4.48731600	0.70423300	20.54522300
C	8.08811400	9.17918400	19.79335700
H	8.81696800	9.97704900	19.58717900
H	7.54436700	8.97861200	18.85845100
C	-0.08316500	6.12098300	24.24221600
H	-0.63746800	5.94100300	23.31480900
H	-0.78012100	6.45393800	25.01664200
C	8.84483800	7.94947800	20.26200600
H	9.62131900	7.71321400	19.52002100
H	9.33282700	8.16347900	21.21625600
C	7.49063800	6.21733900	19.33368400
H	8.27638900	6.06279200	18.58042100
H	6.70908500	6.84952700	18.88979700
C	6.35556700	10.65488000	20.39298000
H	5.71108700	10.33428000	19.56137600
H	6.96424700	11.50375400	20.04900000
C	4.48401100	6.91624700	26.87752900
H	3.86965600	7.73052700	27.27574800
H	5.51684000	7.06664400	27.19471500
H	4.09724400	5.97982700	27.28606400
O	1.03229500	6.61670100	19.40738000
C	1.76628500	5.46246000	19.00615200
H	1.32863900	4.55203700	19.42481000
H	1.70358100	5.43706500	17.91754100
H	2.81113200	5.52933100	19.32153500

1_Z.log

SCF (wB97xd) = -2616.32777917
E(SCF)+ZPE(0 K)= -2615.776194
H(298 K)= -2615.736390

G(298 K)= -2615.850307
Lowest Frequency = 17.1581cm-1

Co	2.473756	6.418500	23.020104
K	5.792014	7.558927	22.009983
O	1.587805	6.207450	21.335570
O	3.181676	8.109933	22.533163
O	4.001943	5.627662	22.195808
O	4.699655	10.124089	21.931437
O	3.269660	6.439898	24.785506
O	5.974807	5.111954	20.601812
O	8.148048	6.883229	20.576770
O	7.259157	9.653249	20.845061
O	5.348436	7.281485	24.586225
O	0.418207	8.117214	21.095771
N	1.733528	4.725913	23.518915
N	0.944936	7.163351	23.862532
C	4.114412	4.393751	21.836885
C	5.213035	4.046699	20.978970
C	2.886787	9.221958	23.119489
C	3.712352	10.363490	22.841293
C	0.829554	7.029212	20.695330
C	0.836566	8.372968	24.259402
H	-0.058625	8.652171	24.821070
C	3.501774	2.009282	21.825504
H	2.829492	1.223391	22.159494
C	4.394356	6.849556	25.241153
C	0.620886	4.870558	24.451981
H	1.035195	4.950464	25.462015
H	-0.053995	4.010909	24.407371
C	1.580452	10.679114	24.614059
H	0.735285	10.796571	25.287360
C	5.436217	2.741360	20.592051
H	6.277336	2.491195	19.955122
C	3.253821	3.340521	22.240128
C	2.420513	11.738590	24.364204
H	2.262669	12.699658	24.841107
C	2.126403	3.578791	23.108737
H	1.573503	2.696965	23.443783
C	5.570434	11.183807	21.578962
H	6.186798	11.469121	22.441550
H	4.997476	12.059233	21.247290
C	1.796164	9.419858	24.005068
C	3.494191	11.576198	23.462799
H	4.144243	12.421123	23.264652
C	7.113491	4.880367	19.791829
H	6.831972	4.353121	18.871087
H	7.848276	4.275766	20.339064
C	4.573178	1.708433	21.018534
H	4.764326	0.688237	20.704502
C	8.169036	9.238141	19.845809
H	8.885693	10.042935	19.624031
H	7.629463	9.008720	18.915013
C	-0.092477	6.167995	24.088896
H	-0.648406	6.040597	23.153531
H	-0.785839	6.480753	24.875075
C	8.943929	8.031284	20.343911
H	9.740178	7.802507	19.621109
H	9.408081	8.270294	21.304199
C	7.703903	6.217868	19.410819
H	8.544796	6.042575	18.724570
H	6.946445	6.812020	18.880933
C	0.461866	6.522397	19.305516
H	1.344871	6.577448	18.660601
H	-0.332874	7.130992	18.871626
H	0.150515	5.475634	19.347697
C	6.439645	10.727181	20.431559
H	5.809529	10.417672	19.584857
H	7.056781	11.578063	20.108305
C	4.480964	6.789352	26.760635
H	3.875897	7.600711	27.178682
H	5.512493	6.910968	27.094304
H	4.073648	5.847201	27.134919

Co_bowl_R.log

SCF (wB97xd) = -2809.39118987
E(SCF)+ZPE(0 K)= -2808.752172
H(298 K)= -2808.707358
G(298 K)= -2808.831301
Lowest Frequency = -526.5618cm-1

O	0.947437	-0.037454	0.563657
C	2.222034	0.056900	0.381572
C	3.000708	-1.151817	0.370886
C	-3.646824	-0.872904	-0.671607
C	-2.894837	0.263277	-0.222420
O	-1.776861	0.077981	0.414498
C	-3.458984	1.539440	-0.483830
C	-2.791713	2.747225	-0.063869
H	-3.366554	3.675520	-0.110500
N	-1.583320	2.804618	0.349620
C	-0.964684	4.048483	0.787471
H	-0.889021	4.020234	1.878072
H	-1.554554	4.918539	0.485270
C	0.424715	4.070288	0.167071
N	0.991620	2.727216	0.307533
C	2.255600	2.546135	0.208647
H	2.889554	3.430317	0.101457
C	2.929106	1.273562	0.199110
C	4.328203	1.276993	-0.019964
H	4.833937	2.228572	-0.160219
C	5.034357	0.100220	-0.071791
C	4.358909	-1.122635	0.125256
H	4.929523	-2.042941	0.080217
C	-4.846023	-0.712697	-1.343522
H	-5.395453	-1.578322	-1.694436
C	-5.382188	0.565903	-1.584986
C	-4.698357	1.675572	-1.147891
H	-5.096510	2.672005	-1.318794
H	-6.326247	0.661525	-2.109717
H	6.102565	0.097613	-0.257956
H	0.345282	4.294079	-0.902350
H	1.063807	4.821016	0.639752
O	2.285644	-2.285925	0.618077
O	-3.096632	-2.089744	-0.394002
C	-3.638631	-3.222986	-1.055814
H	-3.599051	-3.068758	-2.141829
H	-4.682479	-3.381325	-0.756695
C	-2.859851	-4.459095	-0.673851
H	-3.354499	-5.317734	-1.149497
H	-2.889172	-4.607798	0.415728
C	2.942491	-3.538320	0.516670
H	3.192335	-3.740461	-0.533379
H	3.871780	-3.543434	1.099905
C	2.043381	-4.608176	1.112421

H	2.566660	-5.572812	1.037536
H	1.878716	-4.386466	2.170036
O	0.756590	-4.694148	0.543761
O	-1.520342	-4.348562	-1.110157
C	0.669696	-5.251372	-0.753702
H	1.251038	-6.182433	-0.814270
H	1.053669	-4.553159	-1.511193
C	-0.787928	-5.559876	-1.016330
H	-1.170652	-6.171939	-0.189076
H	-0.888717	-6.131595	-1.948525
Co	-0.343120	1.360341	0.394538
O	-0.321833	1.357675	-1.517866
O	-0.250610	-0.838685	-2.024446
C	-0.323895	0.352153	-2.325415
C	-0.466946	0.769708	-3.782483
H	-0.190048	-0.051621	-4.445057
H	0.140837	1.650987	-3.999581
H	-1.513823	1.033689	-3.966919
K	-0.489134	-2.240153	0.383140
O	-1.035698	-2.642268	3.018298
C	-1.037287	-2.394784	4.241598
O	-0.658660	-1.302995	4.770144
C	-1.559334	-3.458040	5.204535
C	-0.136566	0.179282	3.397412
H	-1.470266	-4.455099	4.768352
H	-1.031704	-3.416315	6.160031
H	-2.619688	-3.261961	5.398080
C	-1.316978	0.991424	3.146050
H	0.127054	-0.593517	2.695835
H	0.597516	0.533776	4.107505
O	-0.323053	1.587161	2.318628
H	-2.099314	0.492329	2.573643
C	-1.850147	1.912195	4.214216
H	-2.453347	1.345026	4.929402
H	-1.031439	2.400180	4.751825
H	-2.483017	2.681638	3.761325

Co_bowl_S.log

SCF (wB97xd) = -2809.39064039
E(SCF)+ZPE(0 K)= -2808.750938
H(298 K)= -2808.706445
G(298 K)= -2808.827754
Lowest Frequency = -542.9304cm-1

O	1.010623	0.009251	0.526815
C	2.264139	0.104742	0.218565
C	3.050692	-1.095711	0.158149
C	-3.579655	-0.934221	-0.627461
C	-2.842860	0.225075	-0.209450
O	-1.722986	0.076288	0.432087
C	-3.425514	1.485146	-0.514815
C	-2.776145	2.717091	-0.138891
H	-3.368471	3.633183	-0.204958
N	-1.565764	2.807361	0.260783
C	-0.967149	4.066163	0.681055
H	-0.895403	4.051487	1.772088
H	-1.569510	4.923006	0.365900
C	0.424069	4.104064	0.065994
N	1.004972	2.763903	0.194925
C	2.262198	2.592607	0.019141
H	2.880792	3.481177	-0.132153
C	2.943540	1.324571	-0.031870
C	4.321614	1.339491	-0.357175
H	4.805426	2.294174	-0.544665
C	5.033684	0.168672	-0.448999
C	4.387246	-1.056978	-0.188292
H	4.960748	-1.973583	-0.263319
C	-4.786472	-0.810654	-1.293735
H	-5.326657	-1.693473	-1.614521
C	-5.343261	0.451684	-1.570723
C	-4.670255	1.583151	-1.175326
H	-5.081835	2.568547	-1.376335
O	-0.199717	-0.890328	-2.015853
C	-0.313597	0.288795	-2.348625
O	-0.317402	1.316835	-1.570245
C	-0.505996	0.660412	-3.812407
H	0.073331	1.549697	-4.070813
H	-0.226607	-0.172323	-4.459481
H	-1.563811	0.893615	-3.974730
H	-6.292536	0.517564	-2.090616
H	6.084972	0.173939	-0.714500
Co	-0.308393	1.381683	0.341314
K	-0.379540	-2.246718	0.417318
H	0.349097	4.337888	-1.001483
H	1.055192	4.854889	0.549136
O	2.370836	-2.232825	0.467212
O	-3.007804	-2.137836	-0.330098
C	-3.540096	-3.287828	-0.971597
H	-3.538724	-3.136299	-2.058654
H	-4.569910	-3.470696	-0.639350
C	-2.718686	-4.504444	-0.618418
H	-3.215504	-5.374786	-1.069809
H	-2.697231	-4.651470	0.471683
C	3.051211	-3.474656	0.400856
H	3.292141	-3.710744	-0.644345
H	3.988440	-3.436682	0.970838
C	2.176824	-4.537115	1.044536
H	2.720130	-5.493102	1.008429
H	1.998768	-4.268250	2.088625
O	0.891752	-4.672943	0.481590
O	-1.403202	-4.365925	-1.114785
C	0.811721	-5.223617	-0.817229
H	1.415194	-6.139791	-0.890820
H	1.170198	-4.510308	-1.573263
C	-0.641879	-5.562421	-1.065367
H	-0.995873	-6.203985	-0.247965
H	-0.746238	-6.111797	-2.010337
O	-0.019085	-2.713846	3.146821
C	0.616020	-2.318045	4.144867
O	0.577491	-1.137476	4.617657
C	1.554819	-3.291703	4.850836
C	-0.277256	0.232372	3.322563
H	1.241366	-4.324447	4.684327
H	1.610186	-3.082411	5.921343
H	2.560112	-3.166231	4.432586
C	0.684637	1.304878	3.115140
H	-1.098392	0.396336	4.006224
H	-0.340416	-0.563759	2.603136
O	-0.386732	1.664681	2.254086
C	0.945621	2.314108	4.204744
H	1.591463	1.011632	2.585111
H	1.647274	1.902432	4.936345
H	1.383797	3.219801	3.774211
H	0.016886	2.581488	4.717467

Co_Z_bottom_R.log

SCF (wB97xd) = -2809.37545570
E(SCF)+ZPE(0 K)= -2808.761021
H(298 K)= -2808.716646
G(298 K)= -2808.837199
Lowest Frequency = -518.2418cm-1

Co	5.120248	7.611259	21.322515
O	4.614248	5.914052	22.027806
O	3.654353	8.398184	22.244003
O	3.760001	4.023316	23.601245
O	6.353190	7.939245	22.752625
O	1.353041	8.852135	23.339132
O	0.498880	7.240994	25.496855
O	1.543658	4.458638	25.337174
O	5.251448	7.299363	24.612188
N	5.691811	9.276879	20.581429
N	6.587868	6.859730	20.374275
C	3.162261	9.572824	22.034563
C	1.899048	9.885567	22.644572
C	5.447454	4.949555	22.262777
C	5.030194	3.891839	23.137236
C	7.187553	5.771484	20.680819
H	8.105775	5.519300	20.144506
C	3.167116	11.865106	21.135107
H	3.667341	12.627478	20.543808
C	6.243183	7.721758	24.016111
C	6.995297	9.168052	19.933590
H	7.768326	9.290182	20.699206
H	7.125791	9.936506	19.166400
C	7.573764	3.736095	21.989862
H	8.551755	3.674259	21.520381
C	1.328500	11.134338	22.508149
H	0.381886	11.360953	22.986209
C	3.776120	10.592256	21.263570
C	7.165961	2.770019	22.878711
H	7.815482	1.940561	23.135733
C	5.042899	10.380580	20.609154
H	5.479332	11.243547	20.099410
C	3.213141	3.002760	24.420403
H	3.724653	2.983993	25.391579
H	3.329920	2.023934	23.937914
C	6.730786	4.828060	21.672294
C	5.878731	2.848129	23.450344
H	5.562791	2.072199	24.138707
C	0.088405	9.024594	23.956178
H	-0.647691	9.384705	23.225695
H	0.165711	9.753010	24.773887
C	1.966629	12.137376	21.745948
H	1.502076	13.112547	21.649900
C	0.173034	4.791192	25.471005
H	-0.347973	4.013265	26.050232
H	-0.285762	4.850559	24.474241
C	7.078645	7.764085	19.343838
H	6.413609	7.684297	18.477712
H	8.098548	7.512622	19.039258
C	0.041447	6.108537	26.215447
H	-1.011956	6.244833	26.500763
H	0.641992	6.070852	27.128472
C	-0.376609	7.681988	24.472564
H	-1.394937	7.802224	24.870467
H	-0.398134	6.960377	23.645018
C	1.736967	3.272403	24.593192
H	1.262183	3.374158	23.608076
H	1.288068	2.415850	25.118226
C	7.529172	8.012796	24.777307
H	8.223746	7.181286	24.617180
H	7.330251	8.102102	25.846276
H	8.007526	8.921444	24.405062
K	2.745876	6.542383	23.908895
O	0.850325	5.467447	22.110508
C	0.620809	4.927012	21.009676
O	1.120691	5.285624	19.896993
C	-0.300790	3.711127	20.971159
C	2.972858	6.054291	19.345332
H	-0.961350	3.698320	21.840803
H	-0.888434	3.689633	20.050560
H	0.316358	2.805838	20.994876
C	2.583004	7.279477	20.030206
H	3.038732	6.078078	18.272098
H	3.479117	5.275495	19.894679
O	3.961723	7.503309	19.756287
C	1.543972	8.206820	19.449005
H	1.677643	9.216691	19.848209
H	1.595419	8.241791	18.361747
H	0.552080	7.851702	19.744048
H	2.453047	7.175657	21.111761

Co_Z_bottom_S.log

SCF (wB97xd) = -2809.39874581
E(SCF)+ZPE(0 K)= -2808.759033
H(298 K)= -2808.714721
G(298 K)= -2808.834532
Lowest Frequency = -506.4068cm-1

Co	5.142753	7.644259	21.399454
O	4.703543	5.943533	22.134347
O	3.720380	8.451211	22.367460
O	3.903309	4.028308	23.693314
O	6.421385	8.009597	22.778344
O	1.405717	8.849456	23.451812
O	0.451174	7.111107	25.457007
O	1.573541	4.355530	25.270762
O	5.342596	7.365903	24.651277
N	5.652427	9.307306	20.596941
N	6.579731	6.897235	20.389616
C	3.190355	9.609111	22.139682
C	1.920949	9.890904	22.747055
C	5.540410	4.972319	22.310245
C	5.155293	3.899861	23.182667
C	7.199310	5.809961	20.659963
H	8.091222	5.566104	20.077110
C	3.107967	11.870295	21.173491
H	3.573848	12.629799	20.551453
C	6.323640	7.798259	24.045588
C	6.936059	9.205288	19.908156
H	7.730539	9.342915	20.648827
H	7.034071	9.968073	19.130561
C	7.649408	3.759122	21.931501
H	8.606731	3.702045	21.420416
C	1.309531	11.118190	22.588036
H	0.359223	11.323815	23.068362
C	3.755307	10.620210	21.325624
C	7.276440	2.780290	22.822153

H	7.934727	1.946543	23.040136
C	5.001472	10.409549	20.632209
H	5.413782	11.266756	20.093724
C	3.330014	2.957983	24.424269
H	3.780219	2.898115	25.423517
H	3.495415	2.006878	23.902498
C	6.796346	4.857100	21.664710
C	6.012529	2.850694	23.446541
H	5.721297	2.066267	24.136712
C	0.086675	8.944493	23.964388
H	-0.599222	9.303407	23.186229
H	0.062313	9.641829	24.811534
C	1.908801	12.121749	21.797176
H	1.413542	13.079753	21.683799
C	0.190496	4.657481	25.318389
H	-0.354208	3.847579	25.827791
H	-0.198572	4.748040	24.294355
C	7.013912	7.797951	19.330098
H	6.314327	7.700206	18.494136
H	8.023800	7.557716	18.985543
C	-0.023167	5.941567	26.100476
H	-1.096471	6.042340	26.318884
H	0.515896	5.881964	27.049917
C	-0.360714	7.563513	24.386759
H	-1.410825	7.627540	24.707870
H	-0.288575	6.878483	23.530336
C	1.840108	3.195364	24.509410
H	1.432648	3.317956	23.496275
H	1.372729	2.315667	24.977015
C	7.611465	8.114750	24.794162
H	8.328659	7.307161	24.613236
H	7.424311	8.184605	25.866683
H	8.057832	9.042316	24.428444
K	2.802562	6.510239	23.921132
O	1.008472	5.471219	22.116695
C	0.510793	4.915446	21.116814
O	1.034313	4.866884	19.960695
C	-0.829386	4.203855	21.295878
C	2.875033	5.910646	19.832078
H	-1.493889	4.809328	21.918515
H	-1.308223	3.986514	20.339354
H	-0.656003	3.258816	21.822842
C	2.563519	7.318098	19.992058
H	3.021269	5.506980	18.839053
H	3.201900	5.365643	20.701989
O	3.985050	7.317760	19.868697
C	1.858453	8.086940	18.904836
H	2.022729	9.160013	19.047614
H	2.229194	7.799078	17.916250
H	0.782579	7.892152	18.949808
H	2.213047	7.569996	20.990393

Co _Z_top_R.log

SCF (wB97xd) = -2809.39218667
E(SCF)+ZPE(0 K)= -2808.753136
H(298 K)= -2808.708267
G(298 K)= -2808.832643
Lowest Frequency = -521.2920cm-1

Co	-0.184536	6.086030	13.867034
O	-0.625132	4.402542	14.636248
O	-1.684393	6.871371	14.747812
O	-1.630394	2.378363	15.899359
O	-1.217274	5.754533	12.293982
O	-4.160265	7.172224	15.559821
O	-5.199622	5.415871	17.471996
O	-4.071708	2.698501	17.278446
O	-3.099631	4.822982	13.122987
N	0.344463	7.760197	13.115730
N	1.344137	5.372432	12.971146
C	-2.277590	7.966923	14.393732
C	-3.613564	8.205352	14.862949
C	0.134360	3.360790	14.709060
C	-0.353519	2.231401	15.450402
C	1.913767	4.251669	13.215611
H	2.846155	4.025234	12.691983
C	-2.372987	10.190535	13.334812
H	-1.883220	10.951205	12.733021
C	-2.401561	5.248823	12.204175
C	1.702194	7.706155	12.583344
H	2.397618	7.848953	13.416404
H	1.869506	8.485936	11.834948
C	2.189434	2.069760	14.304029
H	3.166072	2.005673	13.831815
C	-4.258716	9.398528	14.601395
H	-5.256578	9.577691	14.985488
C	-1.686802	8.981395	13.599057
C	1.725243	1.037609	15.084366
H	2.329261	0.153071	15.253571
C	-0.358626	8.828324	13.058689
H	0.072609	9.700715	12.560918
C	-2.270188	1.291154	16.546662
H	-1.891543	1.189414	17.571421
H	-2.084186	0.356198	16.003894
C	1.408884	3.234272	14.103769
C	0.436522	1.119083	15.655780
H	0.074820	0.290692	16.254890
C	-5.507029	7.268814	15.992692
H	-6.150147	7.618996	15.175712
H	-5.581017	7.971143	16.832435
C	-3.636400	10.403492	13.832540
H	-4.162851	11.331362	13.638131
C	-5.459885	2.961928	17.344707
H	-5.977513	2.147268	17.872909
H	-5.882395	3.020798	16.330096
C	1.882802	6.312812	11.995076
H	1.294279	6.219496	11.076505
H	2.932588	6.102394	11.771358
C	-5.692016	4.252371	18.112667
H	-6.769000	4.354082	18.308901
H	-5.169958	4.197955	19.071449
C	-5.984774	5.891936	16.398631
H	-7.040440	5.969243	16.696078
H	-5.924159	5.216691	15.533079
C	-3.762452	1.536009	16.538961
H	-4.112455	1.643298	15.501277
H	-4.256938	0.657379	16.978651
C	-2.922496	5.242093	10.772039
H	-2.147032	4.911085	10.077176
H	-3.202337	6.263596	10.494019
H	-3.800901	4.600736	10.686383
K	-2.804374	4.861096	16.035901
O	-1.610842	5.270619	18.441414
C	-0.713308	5.486282	19.280639
O	0.504667	5.722930	19.005685

C	-1.064078	5.440007	20.765524
C	0.885146	5.796681	16.950693
H	-2.143439	5.507664	20.914480
H	-0.712454	4.489778	21.181494
H	-0.559621	6.244191	21.306835
C	0.630655	7.155983	16.501794
H	0.115322	5.056070	16.814616
H	1.900557	5.493990	17.164149
O	1.040378	6.482042	15.317579
H	-0.422662	7.436755	16.487539
C	1.545892	8.282225	16.909004
H	1.286500	8.630622	17.913171
H	2.590116	7.955161	16.907356
H	1.437260	9.119105	16.212164

Co_Z_top_S.log

SCF (wB97xd) = -2809.39194122
E(SCF)+ZPE(0 K)= -2808.752929
H(298 K)= -2808.708059
G(298 K)= -2808.832655
Lowest Frequency = -511.8462cm-1

Co	-0.281214	6.057260	13.987148
O	-0.745735	4.311588	14.579932
O	-1.811759	6.778720	14.871759
O	-1.736133	2.231227	15.758593
O	-1.252395	5.860732	12.354793
O	-4.320463	6.998834	15.598614
O	-5.363562	5.164205	17.420990
O	-4.184655	2.477092	17.149354
O	-3.169914	4.895286	13.055145
N	0.269598	7.784221	13.383653
N	1.285894	5.417317	13.097014
C	-2.409149	7.881837	14.550301
C	-3.767547	8.073481	14.973574
C	0.038667	3.286579	14.649953
C	-0.444729	2.125085	15.341204
C	1.860029	4.288122	13.284836
H	2.812180	4.104981	12.780348
C	-2.501602	10.153439	13.599703
H	-2.001205	10.952161	13.059000
C	-2.441888	5.383029	12.193038
C	1.646411	7.772925	12.901810
H	2.310361	7.848945	13.768626
H	1.840681	8.609029	12.224047
C	2.142980	2.065279	14.287366
H	3.139511	2.043368	13.854724
C	-4.425009	9.266041	14.740919
H	-5.442201	9.408457	15.087981
C	-1.803687	8.945164	13.834714
C	1.676706	0.996477	15.015509
H	2.297448	0.122729	15.180082
C	-0.448960	8.842767	13.350316
H	-0.011497	9.747339	12.919923
C	-2.357995	1.120022	16.382360
H	-1.978573	1.003059	17.405265
H	-2.155601	0.200127	15.820054
C	1.339875	3.215292	14.095912
C	0.366861	1.027190	15.541574
H	0.007440	0.172545	16.103987
C	-5.678846	7.057644	15.999563
H	-6.308372	7.423199	15.178636
H	-5.786602	7.730175	16.859892
C	-3.790154	10.318229	14.049396
H	-4.326371	11.244728	13.876247
C	-5.578513	2.712049	17.214636
H	-6.082982	1.871656	17.714369
H	-5.995505	2.793350	16.199371
C	1.851804	6.429727	12.212757
H	1.293465	6.406970	11.271363
H	2.909065	6.238566	12.008171
C	-5.842525	3.973471	18.019120
H	-6.923554	4.049287	18.204909
H	-5.331107	3.898988	18.982325
C	-6.138801	5.659345	16.349295
H	-7.201076	5.706881	16.629023
H	-6.048985	5.014415	15.463372
C	-3.853639	1.340754	16.378388
H	-4.203603	1.471048	15.343402
H	-4.334199	0.442345	16.792679
C	-2.921661	5.501155	10.751304
H	-3.147879	6.551163	10.538608
H	-3.823555	4.906746	10.597583
H	-2.141257	5.184124	10.055210
K	-2.918935	4.706994	16.039991
O	-1.877686	5.316046	18.476294
C	-1.277837	5.978919	19.346818
O	-0.355984	6.823622	19.122058
C	-1.634994	5.757095	20.815153
C	0.206327	6.927625	17.094950
H	-1.592468	6.695339	21.373281
H	-2.624977	5.306934	20.912946
H	-0.899518	5.074687	21.255110
C	0.745026	5.627552	16.741140
H	0.883135	7.727989	17.359863
H	-0.819464	7.140653	16.841787
O	0.855225	6.372970	15.533021
C	2.063555	5.157327	17.298144
H	1.920166	4.771639	18.312043
H	2.461863	4.351977	16.672630
H	2.789068	5.975845	17.330433
H	-0.002169	4.837578	16.702142

Co_bowl_R.log

SCF (wB97xd) = -2884.61744522
E(SCF)+ZPE(0 K)= -2883.972485
H(298 K)= -2883.927068
G(298 K)= -2884.052225
Lowest Frequency = -543.7792cm-1

O	0.961337	-0.028834	0.554092
C	2.234785	0.066763	0.363659
C	3.014884	-1.140906	0.352862
C	-3.641574	-0.876249	-0.643605
C	-2.887218	0.263125	-0.206467
O	-1.763714	0.082165	0.421917
C	-3.455095	1.537261	-0.469466
C	-2.786191	2.748061	-0.060707
H	-3.362658	3.675329	-0.107869
N	-1.574968	2.809190	0.343661
C	-0.955197	4.055832	0.771649
H	-0.872257	4.032040	1.861796
H	-1.548285	4.923707	0.469458
C	0.430096	4.076818	0.142044

N	0.999970	2.735306	0.285125
C	2.263489	2.555514	0.179526
H	2.895631	3.439989	0.064188
C	2.938770	1.283643	0.171660
C	4.336385	1.287942	-0.056437
H	4.839967	2.239570	-0.203928
C	5.043827	0.111855	-0.108043
C	4.371430	-1.111107	0.098290
H	4.942869	-2.030895	0.053111
C	-4.846599	-0.720950	-1.306137
H	-5.397909	-1.589010	-1.647963
C	-5.386338	0.555801	-1.549489
C	-4.700166	1.668470	-1.123823
H	-5.101050	2.663550	-1.296273
H	-6.334956	0.647654	-2.066627
H	6.110798	0.109989	-0.301189
H	0.343373	4.295343	-0.927904
H	1.071125	4.830783	0.606909
O	2.302564	-2.274685	0.609310
O	-3.087382	-2.090942	-0.364516
C	-3.635016	-3.228882	-1.013632
H	-3.605769	-3.081964	-2.100987
H	-4.675888	-3.385575	-0.703527
C	-2.852062	-4.462011	-0.630542
H	-3.351392	-5.324320	-1.094485
H	-2.869939	-4.602273	0.460331
C	2.959329	-3.527254	0.508341
H	3.201102	-3.733865	-0.542721
H	3.893047	-3.529327	1.084492
C	2.064842	-4.594526	1.115620
H	2.587655	-5.559454	1.041822
H	1.907542	-4.367603	2.173261
O	0.774105	-4.683241	0.556342
O	-1.517087	-4.354741	-1.081747
C	0.676987	-5.252688	-0.735138
H	1.258264	-6.184022	-0.791562
H	1.054636	-4.561535	-1.502270
C	-0.782593	-5.564531	-0.983016
H	-1.157891	-6.169368	-0.147125
H	-0.890557	-6.144688	-1.909159
Co	-0.332318	1.367125	0.388063
O	-0.323844	1.356439	-1.526047
O	-0.255976	-0.840934	-2.028268
C	-0.332117	0.349484	-2.331209
C	-0.487012	0.763164	-3.788206
H	-0.216027	-0.059988	-4.450969
H	0.119369	1.643573	-4.012661
H	-1.535247	1.027233	-3.964662
K	-0.470766	-2.232018	0.376097
O	-1.020583	-2.625616	3.016983
C	-1.010573	-2.326239	4.217009
O	-0.591069	-1.262239	4.754450
C	-0.083125	0.182399	3.410066
C	-1.267643	0.988396	3.145018
H	0.201523	-0.573080	2.696253
H	0.652540	0.561477	4.106260
O	-0.298642	1.604385	2.308478
H	-2.047025	0.473531	2.581832
C	-1.813221	1.900241	4.215686
H	-2.402428	1.323180	4.934744
H	-1.001265	2.404889	4.748279
H	-2.462144	2.656759	3.763873
O	-1.519622	-3.278747	5.058916
C	-1.546347	-2.989824	6.451946
H	-2.152186	-2.103662	6.660263
H	-1.994185	-3.863472	6.927068
H	-0.537127	-2.833002	6.842595

Co_bowl_S.log

SCF (wB97xd) =	-2884.616559
E(SCF)+ZPE(0 K)=	-2883.971114
H(298 K)=	-2883.925805
G(298 K)=	-2884.049767

Lowest Frequency = -546.8035cm-1

O	1.004697	0.039070	0.495373
C	2.260272	0.147899	0.200328
C	3.061230	-1.043731	0.163183
C	-3.573507	-0.956680	-0.635800
C	-2.849591	0.211560	-0.219882
O	-1.724600	0.076720	0.416166
C	-3.448856	1.464598	-0.521981
C	-2.811181	2.705048	-0.152896
H	-3.414000	3.614236	-0.219219
N	-1.599425	2.810575	0.238765
C	-1.009902	4.077870	0.645979
H	-0.927756	4.070664	1.736354
H	-1.623551	4.926715	0.331015
C	0.375306	4.125287	0.017686
N	0.972429	2.793353	0.155770
C	2.231331	2.634173	-0.019435
H	2.840294	3.527955	-0.178847
C	2.927039	1.373152	-0.056637
C	4.307403	1.400003	-0.371545
H	4.781919	2.357974	-0.565857
C	5.033580	0.236437	-0.444148
C	4.399912	-0.993719	-0.173021
H	4.984854	-1.904329	-0.231068
C	-4.784324	-0.847782	-1.297269
H	-5.314246	-1.737028	-1.617446
C	-5.358189	0.407738	-1.570203
C	-4.697708	1.547319	-1.176723
H	-5.122456	2.527593	-1.375446
O	-0.205155	-0.870427	-2.052437
C	-0.339661	0.308579	-2.379245
O	-0.348542	1.333128	-1.597206
C	-0.554958	0.681831	-3.839362
H	-1.617828	0.900911	-3.987817
H	0.008790	1.579854	-4.101941
H	-0.272177	-0.144965	-4.492556
H	-6.310410	0.462255	-2.086009
H	6.086781	0.250996	-0.701606
Co	-0.326482	1.399676	0.316067
K	-0.355591	-2.231417	0.354077
H	0.287697	4.346310	-1.051550
H	1.002103	4.888580	0.486658
O	2.391260	-2.183802	0.482809
O	-2.985420	-2.153332	-0.341258
C	-3.503271	-3.311389	-0.980598
H	-3.499630	-3.163881	-2.068196
H	-4.532226	-3.503696	-0.651220
C	-2.672087	-4.518965	-0.617661
H	-3.161962	-5.396769	-1.062061
H	-2.650786	-4.656055	0.473677
C	3.083640	-3.420391	0.431266

H	3.332557	-3.662218	-0.610766
H	4.017100	-3.368358	1.006230
C	2.214395	-4.484102	1.079626
H	2.765553	-5.436029	1.053223
H	2.029607	-4.208862	2.121476
O	0.934551	-4.634835	0.506337
O	-1.356312	-4.376496	-1.113473
C	0.867025	-5.208610	-0.783003
H	1.477380	-6.121631	-0.836754
H	1.225956	-4.506866	-1.549838
C	-0.582328	-5.564138	-1.036065
H	-0.936717	-6.194049	-0.209891
H	-0.674207	-6.131421	-1.971625
O	-0.138838	-2.737924	3.105752
C	0.627332	-2.318327	3.980725
O	0.696951	-1.149435	4.460240
C	-0.226380	0.211617	3.279494
C	0.688105	1.327011	3.072513
H	-1.044438	0.339248	3.975426
H	-0.301891	-0.546082	2.520817
O	-0.394313	1.686657	2.229073
C	0.932071	2.313119	4.188112
H	1.601445	1.074992	2.532155
H	1.648941	1.899882	4.904052
H	1.344694	3.240309	3.778847
H	0.000632	2.544122	4.713537
C	2.452388	-2.804107	5.448685
H	3.050641	-3.681949	5.695535
H	3.096799	-2.018344	5.044589
H	1.953412	-2.431581	6.347374
O	1.507548	-3.240879	4.478365

Co_Z_bottom_R.log

SCF (wB97xd) = -2884.62666366
E(SCF)+ZPE(0 K)= -2883.981036
H(298 K)= -2883.936047
G(298 K)= -2884.057402
Lowest Frequency = -540.1861cm-1

Co	5.107095	7.648819	21.283895
O	4.549835	5.951128	21.950560
O	3.635433	8.439261	22.190950
O	3.630292	4.036744	23.459473
O	6.336834	7.941780	22.730417
O	1.347629	8.921530	23.301285
O	0.490738	7.310237	25.454191
O	1.452387	4.501455	25.232499
O	5.224999	7.287645	24.577636
N	5.701801	9.312820	20.564367
N	6.564320	6.884434	20.326344
C	3.161863	9.624933	21.994946
C	1.905304	9.950933	22.610442
C	5.340688	4.940649	22.133385
C	4.887051	3.867745	22.971416
C	7.109196	5.753577	20.576377
H	8.013443	5.483646	20.025126
C	3.199795	11.923723	21.113970
H	3.711234	12.683814	20.529433
C	6.222343	7.710351	23.989816
C	7.015921	9.197032	19.939782
H	7.773606	9.295461	20.723881
H	7.172409	9.977333	19.189618
C	7.408778	3.646197	21.787980
H	8.378730	3.561379	21.305532
C	1.352075	11.208700	22.482941
H	0.409996	11.446020	22.964604
C	3.791500	10.642094	21.234235
C	6.967117	2.662661	22.640474
H	7.581247	1.795171	22.855471
C	5.062085	10.421900	20.589318
H	5.512382	11.283875	20.090097
C	3.053710	3.010916	24.251566
H	3.577374	2.939620	25.213870
H	3.126011	2.045125	23.735213
C	6.612824	4.787342	21.526170
C	5.691344	2.775658	23.231631
H	5.348814	1.987840	23.893146
C	0.094192	9.115006	23.934961
H	-0.643865	9.493793	23.216061
H	0.196386	9.836957	24.755665
C	2.002765	12.207598	21.726356
H	1.551399	13.189579	21.637162
C	0.096472	4.871412	25.405699
H	-0.432984	4.100489	25.986617
H	-0.386757	4.957695	24.422826
C	7.092394	7.803643	19.325978
H	6.447443	7.750941	18.443183
H	8.115731	7.543047	19.041126
C	0.020156	6.181499	26.170154
H	-1.021730	6.342923	26.483360
H	0.641923	6.115615	27.067196
C	-0.391497	7.778983	24.448405
H	-1.400334	7.917891	24.863974
H	-0.442375	7.066374	23.614844
C	1.591297	3.329037	24.453380
H	1.104086	3.471496	23.479543
H	1.121441	2.475368	24.964977
C	7.506404	7.984640	24.760922
H	7.304318	8.056244	25.830639
H	7.987550	8.898805	24.405972
H	8.200551	7.155203	24.588979
K	2.719009	6.582939	23.862284
O	0.680721	5.610791	22.149908
C	0.510941	5.008390	21.083175
O	0.925268	5.323595	19.931458
C	2.373595	6.757183	19.954165
C	3.539201	6.221445	19.264247
H	2.348050	6.737427	21.030637
H	1.739003	7.454677	19.424291
O	4.020969	7.487594	19.693224
H	4.019888	5.382472	19.769268
C	3.515767	6.065220	17.764039
H	2.967708	5.158269	17.491506
H	3.032840	6.925437	17.290887
H	4.537649	5.980452	17.382162
O	-0.219772	3.853606	21.178319
C	-0.414923	3.090312	19.993452
H	0.540445	2.765553	19.571924
H	-1.000600	2.220762	20.294231
H	-0.962560	3.662458	19.239594

Co_Z_bottom_S.log

SCF (wB97xd) = -2884.62462135
E(SCF)+ZPE(0 K)= -2883.979168

H(298 K)= -2883.934076
G(298 K)= -2884.056173
Lowest Frequency = -524.8654cm-1

Co	5.137870	7.679803	21.409056
O	4.702292	5.960893	22.105443
O	3.713320	8.460370	22.395309
O	3.910814	4.009196	23.621593
O	6.417537	8.017063	22.796354
O	1.391938	8.820355	23.476627
O	0.475039	7.056553	25.472427
O	1.602619	4.309576	25.231487
O	5.342199	7.334135	24.657025
N	5.643319	9.362317	20.645488
N	6.578035	6.960839	20.383068
C	3.174512	9.617867	22.185202
C	1.901043	9.878697	22.792812
C	5.543910	4.989377	22.260041
C	5.163383	3.896877	23.108750
C	7.200829	5.869876	20.629199
H	8.094182	5.642105	20.042031
C	3.078059	11.895177	21.259777
H	3.540282	12.669454	20.653433
C	6.321820	7.780833	24.058950
C	6.928957	9.280403	19.957990
H	7.721134	9.401271	20.703968
H	7.027343	10.062301	19.199669
C	7.657679	3.793440	21.855315
H	8.615248	3.751414	21.343256
C	1.280383	11.103804	22.653790
H	0.326701	11.293368	23.133976
C	3.734621	10.647717	21.391751
C	7.288862	2.793962	22.724578
H	7.950677	1.958489	22.924494
C	4.985392	10.459467	20.699862
H	5.394252	11.329964	20.180430
C	3.349438	2.926762	24.344142
H	3.812742	2.853964	25.336547
H	3.510638	1.983722	23.806618
C	6.800197	4.893363	21.612267
C	6.024821	2.845566	23.350098
H	5.736828	2.045059	24.022876
C	0.079585	8.905208	24.007616
H	-0.618341	9.269729	23.242890
H	0.064426	9.593150	24.862603
C	1.874631	12.125952	21.883365
H	1.372282	13.081954	21.785349
C	0.219171	4.604266	25.309639
H	-0.311560	3.786145	25.820505
H	-0.190726	4.703868	24.294816
C	7.011032	7.887644	19.345928
H	6.312386	7.808491	18.507213
H	8.021799	7.658218	18.996587
C	0.015579	5.878092	26.110816
H	-1.053870	5.973012	26.349470
H	0.571961	5.809367	27.049565
C	-0.355826	7.517857	24.420784
H	-1.400966	7.573355	24.759004
H	-0.295643	6.843697	23.555185
C	1.860320	3.158834	24.452455
H	1.436416	3.291309	23.447389
H	1.402834	2.272564	24.917374
C	7.609249	8.085276	24.813152
H	8.326513	7.280725	24.619349
H	7.421745	8.137328	25.886634
H	8.055707	9.018749	24.462935
K	2.813465	6.487687	23.919040
O	0.972548	5.443084	22.086545
C	0.535628	4.953800	21.039109
O	1.077513	4.916513	19.898553
C	2.870449	5.944019	19.802741
C	2.565375	7.349431	20.009833
H	3.026077	5.581114	18.795322
H	3.231666	5.381456	20.648744
O	3.981806	7.386838	19.874657
C	1.831768	8.138884	18.955612
H	1.980832	9.209541	19.129346
H	2.193996	7.887708	17.953913
H	0.759850	7.924852	19.008634
H	2.218530	7.568396	21.017788
O	-0.705954	4.376154	21.146198
C	-1.276573	3.802652	19.976595
H	-0.652702	2.993277	19.587323
H	-2.245861	3.406576	20.282578
H	-1.415070	4.554161	19.194110

Co_Z_top_R.log

SCF (wB97xd) = -2884.61836543
E(SCF)+ZPE(0 K)= -2883.973458
H(298 K)= -2883.927904
G(298 K)= -2884.054266
Lowest Frequency = -543.8637cm-1

Co	-0.181486	6.081982	13.857960
O	-0.619517	4.397853	14.629023
O	-1.674882	6.868227	14.749655
O	-1.618982	2.372814	15.894892
O	-1.226766	5.754373	12.290633
O	-4.145340	7.172986	15.577134
O	-5.180445	5.408876	17.483561
O	-4.056274	2.692042	17.280075
O	-3.105033	4.823768	13.129388
N	0.344732	7.756047	13.104282
N	1.338740	5.366501	12.949109
C	-2.267198	7.966682	14.402963
C	-3.599531	8.207328	14.881327
C	0.138680	3.354448	14.693774
C	-0.345527	2.224592	15.436693
C	1.908286	4.244602	13.187913
H	2.836189	4.017033	12.656821
C	-2.363095	10.194127	13.352195
H	-1.875115	10.955277	12.749559
C	-2.412386	5.251568	12.207117
C	1.698109	7.699907	12.561314
H	2.400121	7.840085	13.389248
H	1.861267	8.480334	11.812669
C	2.188559	2.060931	14.271555
H	3.161521	1.995990	13.791931
C	-4.242630	9.403540	14.628669
H	-5.237353	9.584305	15.020075
C	-1.678893	8.981956	13.607465
C	1.728703	1.028355	15.054003
H	2.332721	0.142745	15.217522
C	-0.355507	8.826381	13.056027
H	0.074235	9.698866	12.557110

C -2.257091 1.284714 16.542624
H -1.875422 1.181617 17.566079
H -2.072842 0.350517 15.998021
C 1.408373 3.226865 14.078704
C 0.444406 1.110853 15.634977
H 0.085878 0.282259 16.235712
C -5.489895 7.271100 16.016671
H -6.135673 7.627663 15.204577
H -5.557884 7.968931 16.860631
C -3.622315 10.409427 13.859471
H -4.147067 11.339699 13.672074
C -5.444269 2.956155 17.349431
H -5.961507 2.140164 17.875914
H -5.868287 3.018618 16.335630
C 1.871373 6.306993 11.969911
H 1.275195 6.215921 11.056067
H 2.918916 6.094847 11.737426
C -5.673858 4.244386 18.121743
H -6.750429 4.347067 18.319547
H -5.150770 4.186354 19.079696
C -5.969904 5.893417 16.417224
H -7.023657 5.972526 16.720875
H -5.916347 5.222825 15.547539
C -3.749347 1.529739 16.539376
H -4.102304 1.637390 15.502711
H -4.242702 0.651053 16.980216
C -2.943598 5.250408 10.778745
H -2.174781 4.916343 10.077950
H -3.219508 6.273958 10.504375
H -3.826104 4.614066 10.697959
K -2.798325 4.852858 16.028242
O -1.598123 5.265903 18.441803
C -0.679045 5.526781 19.226940
O 0.541317 5.720850 18.962743
C 0.908139 5.773300 16.956240
C 0.650671 7.128668 16.489673
H 0.146304 5.026418 16.802565
H 1.929024 5.472483 17.146489
O 1.053999 6.472364 15.297380
H -0.403723 7.407774 16.485834
C 1.561304 8.258240 16.900908
H 1.305005 8.598774 17.908637
H 2.607578 7.937737 16.891376
H 1.443638 9.098445 16.209643
O -1.042936 5.611257 20.544171
C -0.029849 5.919494 21.494781
H -0.530058 5.942730 22.463707
H 0.752552 5.155625 21.500512
H 0.422634 6.893366 21.288281

Co_Z_top_S.log

SCF (wB97xd) = -2884.61820526
E(SCF)+ZPE(0 K)= -2883.973206
H(298 K)= -2883.927754
G(298 K)= -2884.052663
Lowest Frequency = -536.9342cm-1

Co -0.272705 6.049764 13.975088
O -0.740256 4.304386 14.567617
O -1.788955 6.779275 14.880442
O -1.723871 2.228376 15.759402
O -1.266741 5.862882 12.353425
O -4.293294 7.008878 15.624021
O -5.337658 5.168007 17.438564
O -4.166697 2.479432 17.157774
O -3.180943 4.907079 13.074774
N 0.280484 7.774618 13.367265
N 1.278904 5.402435 13.063128
C -2.385143 7.884882 14.563754
C -3.739569 8.081907 14.997012
C 0.042967 3.278020 14.633125
C -0.436160 2.119181 15.331556
C 1.852107 4.272152 13.246597
H 2.797773 4.085122 12.731421
C -2.475387 10.157669 13.615630
H -1.975912 10.954420 13.071191
C -2.460082 5.391999 12.204586
C 1.650838 7.755785 12.867847
H 2.326243 7.824887 13.726354
H 1.841999 8.592430 12.189851
C 2.141387 2.052029 14.253772
H 3.133609 2.026956 13.811496
C -4.393409 9.277906 14.771295
H -5.407177 9.424298 15.126617
C -1.781219 8.945993 13.843584
C 1.680443 0.986393 14.990040
H 2.301459 0.112293 15.151711
C -0.432211 8.837209 13.344322
H 0.005167 9.740144 12.910387
C -2.343574 1.118961 16.388733
H -1.960186 1.004909 17.410444
H -2.143602 0.197480 15.828210
C 1.338462 3.202775 14.066510
C 0.375640 1.020723 15.528157
H 0.020161 0.168536 16.096776
C -5.650432 7.070666 16.029021
H -6.280790 7.442796 15.211722
H -5.753274 7.738995 16.893172
C -3.759399 10.328082 14.076256
H -4.292695 11.257268 13.908572
C -5.560024 2.717280 17.225032
H -6.065883 1.876661 17.722947
H -5.977716 2.802383 16.210310
C 1.838337 6.412913 12.172785
H 1.266465 6.395713 11.239439
H 2.891422 6.215815 11.952779
C -5.820234 3.976995 18.033384
H -6.900990 4.055535 18.219470
H -5.308968 3.898027 18.996234
C -6.114718 5.672201 16.372541
H -7.175833 5.722331 16.656143
H -6.030547 5.031941 15.482631
C -3.839201 1.340160 16.389676
H -4.192970 1.466863 15.355514
H -4.318646 0.443500 16.809007
C -2.957222 5.515062 10.769230
H -3.195164 6.564579 10.566876
H -3.856137 4.914496 10.621942
H -2.182462 5.209712 10.061812
K -2.913057 4.700625 16.023339
O -1.854138 5.305968 18.469580
C -1.214435 5.968804 19.294442
O -0.293200 6.806545 19.081900
C 0.239657 6.921617 17.100283
C 0.764522 5.621512 16.715047

H	0.932259	7.714648	17.347018
H	-0.779275	7.153731	16.832658
O	0.883559	6.353916	15.504243
C	2.077856	5.131410	17.269990
H	1.932051	4.745866	18.283689
H	2.462579	4.321724	16.641686
H	2.815250	5.939363	17.301190

H	0.009191	4.838284	16.680720
O	-1.559343	5.756241	20.604470
C	-0.850594	6.478597	21.604273
H	-0.984983	7.557224	21.484123
H	-1.273563	6.158387	22.557358
H	0.217735	6.246452	21.577291

Reaction Profile

CO2.log

SCF (wB97xd) = -188.574025127
E(SCF)+ZPE(0 K)= -188.562314
H(298 K)= -188.558737
G(298 K)= -188.583009
Lowest Frequency = 654.2553cm-1

C	-4.08164900	0.32622400	-0.04916100
O	-2.92343100	0.32622400	-0.04916100
O	-5.23986600	0.32622400	-0.04916100

RPO.log

SCF (wB97xd) = -193.085539750
E(SCF)+ZPE(0 K)= -192.999177
H(298 K)= -192.993859
G(298 K)= -193.025528
Lowest Frequency = 208.2426cm-1

C	-0.64250000	-0.26930500	0.02021100
C	0.82141500	-0.26458500	-0.00111800
O	0.08549400	0.95843600	-0.02669100
H	-1.15377400	-0.49957200	-0.91463600
H	1.35376900	-0.50174100	-0.91984900
H	1.36509300	-0.45360600	0.92313100
C	-1.42290800	-0.54412500	1.27629700
H	-2.34429000	0.04588900	1.29424200
H	-1.69620900	-1.60282400	1.32341100
H	-0.83098800	-0.29668800	2.16211000

SPO.log

SCF (wB97xd) = -193.085539838
E(SCF)+ZPE(0 K)= -192.999169
H(298 K)= -192.993853
G(298 K)= -193.025516
Lowest Frequency = 209.7061cm-1

C	-0.64085600	-0.25811300	-0.02518400
C	0.82287700	-0.27469600	0.00092500
O	0.10518800	0.95930600	0.00464100
H	1.36665600	-0.48660300	-0.91829100
H	1.34864900	-0.50516400	0.92514500
C	-1.42171800	-0.54102400	-1.27919000
H	-1.71185500	-1.59582300	-1.31014800
H	-2.33354100	0.06323500	-1.30944300
H	-0.82346100	-0.31731500	-2.16705600
H	-1.15836000	-0.46578600	0.91149500

ICo.log

SCF (wB97xd) = -2884.005056
E(SCF)+ZPE(0 K)= -2883.960302
H(298 K)= -2883.959358
G(298 K)= -2884.082375
Lowest Frequency = 24.2992cm-1

Co	4.52935700	6.84434200	20.98589500
O	3.53465200	5.36038700	21.64637100
O	3.40487600	8.00803100	21.96656700
O	2.30454700	3.77725900	23.34606900
O	5.88124500	6.71891800	22.29432700
O	1.53052500	9.13122200	23.35458000
O	0.55601600	7.80612400	25.63901000
O	0.63808100	4.85929600	25.37206800
O	4.73568500	6.40656600	24.21013700
N	5.52718400	8.28042700	20.21869000
N	5.59971500	5.71830200	19.88148100
C	3.34188900	9.29884400	21.87894700
C	2.32232600	9.96754300	22.63733800
C	4.02631400	4.16493100	21.79094800
C	3.39078800	3.26578500	22.70945800
C	5.80342900	4.46802700	20.07248300
H	6.53463700	3.96311500	19.43689000
C	4.05379400	11.51683700	21.08220200
H	4.73455800	12.10920100	20.47704800
C	5.77557000	6.50871500	23.56507300
C	6.69488000	7.79927800	19.48431100
H	7.50334800	7.63539800	20.20400500
H	7.02175700	8.52519000	18.73508400
C	5.60184900	2.34257100	21.26438100
H	6.45303100	1.99242400	20.68720300
C	2.19695200	11.34153600	22.60531600
H	1.42604600	11.83452500	23.18707700
C	4.20369500	10.10745500	21.09898400
C	4.99322700	1.51849700	22.18019100
H	5.35476800	0.51028300	22.34881300
C	5.27635300	9.53278000	20.33009400
H	5.93499400	10.23263200	19.80999600
C	1.60654000	2.96016600	24.27390600
H	2.24165000	2.76087900	25.14656100
H	1.32931900	2.00597700	23.80844000
C	5.13639800	3.66387300	21.06529700
C	3.87933700	1.98743100	22.90472900
H	3.40908300	1.32589400	23.62303300
C	0.49113400	9.67333500	24.15378700
H	-0.18968400	10.27425600	23.53703400
H	0.91749200	10.30932700	24.94044000
C	3.06864900	12.12668300	21.81998100
H	2.95318400	13.20479300	21.80949600

C	-0.52104100	5.58428500	25.75144500
H	-1.12585300	4.98539600	26.44957600
H	-1.13712200	5.80009500	24.86646400
C	6.29861000	6.47410800	18.84745700
H	5.60391400	6.65240900	18.01991700
H	7.16754500	5.92817600	18.47039300
C	-0.10625300	6.86149300	26.46048600
H	-1.00131800	7.31453300	26.91142500
H	0.59688000	6.61930200	27.26219500
C	-0.28935000	8.52701900	24.75653100
H	-1.14372500	8.94643500	25.30753000
H	-0.66407300	7.87623700	23.95473400
C	0.33440800	3.66490800	24.68193200
H	-0.26913100	3.87883300	23.78972400
H	-0.24004700	2.98441900	25.32812900
C	7.12961500	6.35558100	24.23948700
H	7.03014900	6.46132000	25.32043900
H	7.84403500	7.08505400	23.85215000
H	7.51862900	5.35664800	24.01660000
K	2.08544800	6.47120000	23.64949800
O	-2.29792000	5.84465800	22.69545400
C	-1.20365000	5.62661600	22.14937900
O	-0.17636000	6.33453900	22.13541300
C	1.82461100	6.94782400	19.61727600
C	2.53831100	5.91140300	18.87177200
H	1.47893700	6.73608900	20.62553400
H	1.31068300	7.73646800	19.07469600
O	3.26254700	7.02468400	19.46066700
H	2.71954500	4.98398400	19.40921000
C	2.57458300	5.84820900	17.37544600
H	1.76905400	5.19594600	17.02544400
H	2.43840900	6.84017400	16.93761600
H	3.52371800	5.42915700	17.02991300
O	-1.14546600	4.39995900	21.49332600
C	0.03717600	4.09882200	20.76731300
H	0.08640700	4.68766500	19.84450100
H	0.93423100	4.29112000	21.36206800
H	-0.02314100	3.03863700	20.51297500

TS1Co.log

SCF (wB97xd) = -2884.62666366
E(SCF)+ZPE(0 K)= -2883.981036
H(298 K)= -2883.936047
G(298 K)= -2884.057402
Lowest Frequency = -540.1861cm-1

Co	5.107095	7.648819	21.283895
O	4.549835	5.951128	21.950560
O	3.635433	8.439261	22.190950
O	3.630292	4.036744	23.459473
O	6.336834	7.941780	22.730417
O	1.347629	8.921530	23.301285
O	0.490738	7.310237	25.454191
O	1.452387	4.501455	25.232499
O	5.224999	7.287645	24.577636
N	5.701801	9.312820	20.564367
N	6.564320	6.884434	20.326344
C	3.161863	9.624933	21.994946
C	1.905304	9.950933	22.610442
C	5.340688	4.940649	22.133385
C	4.887051	3.867745	22.971416
C	7.109196	5.753577	20.576377
H	8.013443	5.483646	20.025126
C	3.199795	11.923723	21.113970
H	3.711234	12.683814	20.529433
C	6.222343	7.710351	23.989816
C	7.015921	9.197032	19.939782
H	7.773606	9.295461	20.723881
H	7.172409	9.977333	19.189618
C	7.408778	3.646197	21.787980
H	8.378730	3.561379	21.305532
C	1.352075	11.208700	22.482941
H	0.409996	11.446020	22.964604
C	3.791500	10.642094	21.234235
C	6.967117	2.662661	22.640474
H	7.581247	1.795171	22.855471
C	5.062085	10.421900	20.589318
H	5.512382	11.283875	20.090097
C	3.053710	3.010916	24.251566
H	3.577374	2.939620	25.213870
H	3.126011	2.045125	23.735213
C	6.612824	4.787342	21.526170
C	5.691344	2.775658	23.231631
H	5.348814	1.987840	23.893146
C	0.094192	9.115006	23.934961
H	-0.643865	9.493793	23.216061
H	0.196386	9.836957	24.755665
C	2.002765	12.207598	21.726356
H	1.551399	13.189579	21.637162
C	0.096472	4.871412	25.405699
H	-0.432984	4.100489	25.986617
H	-0.386757	4.957695	24.422826
C	7.092394	7.803643	19.325978
H	6.447443	7.750941	18.443183
H	8.115731	7.543047	19.041126
C	0.020156	6.181499	26.170154
H	-1.021730	6.342923	26.483360
H	0.641923	6.115615	27.067196
C	-0.391497	7.778983	24.448405
H	-1.400334	7.917891	24.863974
H	-0.442375	7.066374	23.614844
C	1.591297	3.329037	24.453380
H	1.104086	3.471496	23.479543
H	1.121441	2.475368	24.964977
C	7.506404	7.984640	24.760922
H	7.304318	8.056244	25.830639
H	7.987550	8.898805	24.405972
H	8.200551	7.155203	24.588979
K	2.719009	6.582939	23.862284
O	0.680721	5.610791	22.149908
C	0.510941	5.008390	21.083175
O	0.925268	5.323595	19.931458
C	2.373595	6.757183	19.954165
C	3.539201	6.221445	19.264247
H	2.348050	6.737427	21.030637
H	1.739003	7.454677	19.424291
O	4.020969	7.487594	19.693224
H	4.019888	5.382472	19.769268
C	3.515767	6.065220	17.764039
H	2.967708	5.158269	17.491506
H	3.032840	6.925437	17.290887
H	4.537649	5.980452	17.382162
O	-0.219772	3.853606	21.178319
C	-0.414923	3.090312	19.993452
H	0.540445	2.765553	19.571924

H	-1.000600	2.220762	20.294231
H	-0.962560	3.662458	19.239594

TS1Co’.log
SCF (wB97xd) = -2884.62384888
E(SCF)+ZPE(0 K)= -2883.978357
H(298 K)= -2883.933132
G(298 K)= -2884.056148
Lowest Frequency = -538.6671cm-1

Co	5.021703	7.624193	21.360221
O	4.400384	5.967411	22.050959
O	3.654041	8.472463	22.367768
O	3.372580	4.050316	23.458502
O	6.381785	7.825068	22.710571
O	1.300702	8.923563	23.344255
O	0.388335	7.367520	25.478827
O	1.219378	4.603399	25.174111
O	5.442588	7.185011	24.654060
N	5.659164	9.267391	20.634286
N	6.367062	6.799989	20.293202
C	3.149085	9.631034	22.091713
C	1.866187	9.947375	22.647898
C	5.079056	4.866121	22.076995
C	4.570466	3.786115	22.870597
C	6.811204	5.608089	20.440134
H	7.654983	5.293139	19.820960
C	3.162922	11.890941	21.118044
H	3.672206	12.637530	20.514625
C	6.393943	7.567469	23.967248
C	6.953279	9.099226	19.983764
H	7.726282	9.119052	20.758066
H	7.145182	9.896075	19.259745
C	6.951441	3.388655	21.466173
H	7.865705	3.237326	20.898751
C	1.291295	11.188621	22.460907
H	0.328130	11.421924	22.900728
C	3.772472	10.625989	21.297331
C	6.469157	2.396255	22.285422
H	6.996145	1.454169	22.388061
C	5.043741	10.389408	20.656775
H	5.504977	11.239876	20.148049
C	2.737041	3.029521	24.208180
H	3.248374	2.896225	25.170139
H	2.758438	2.077290	23.664190
C	6.274189	4.627238	21.352168
C	5.264869	2.600056	22.991868
H	4.889133	1.804838	23.625783
C	0.010784	9.111902	23.898919
H	-0.694068	9.449568	23.128278
H	0.050043	9.859972	24.701093
C	1.945957	12.174159	21.691965
H	1.479434	13.143657	21.556616
C	-0.106499	4.955051	25.512433
H	-0.547208	4.194946	26.174834
H	-0.727936	5.004362	24.606301
C	6.928962	7.727368	19.317412
H	6.263633	7.750851	18.448053
H	7.927003	7.415840	18.996616
C	-0.097194	6.282454	26.247516
H	-1.115828	6.493897	26.603853
H	0.565095	6.214261	27.114282
C	-0.467508	7.783424	24.433217
H	-1.495697	7.905837	24.802789
H	-0.480517	7.047851	23.616926
C	1.292954	3.418055	24.408191
H	0.808112	3.561487	23.430720
H	0.784828	2.593293	24.929154
C	7.766314	7.749944	24.600626
H	8.381370	6.877454	24.355962
H	7.682533	7.825074	25.685786
H	8.267681	8.632988	24.198399
K	2.880495	6.707952	24.221554
O	0.691568	5.344133	20.090540
C	2.234258	6.692878	20.155483
C	3.372719	6.324255	19.322579
H	2.310886	6.512207	21.214595
H	1.534669	7.427498	19.783728
O	3.810033	7.561288	19.866499
H	3.933201	5.464815	19.687422
C	3.237466	6.336581	17.820985
H	2.727132	5.427754	17.488986
H	2.664485	7.207082	17.487778
H	4.227777	6.367416	17.356463
O	0.089043	3.304837	20.797985
C	0.903274	4.126851	20.367104
O	2.197809	3.739065	20.136692
C	2.540102	2.395164	20.430955
H	3.623177	2.329345	20.315227
H	2.264104	2.131972	21.455107
H	2.046573	1.701967	19.742512

IICo.log

SCF (wB97xd) = -2884.018926
E(SCF)+ZPE(0 K)= -2883.974684
H(298 K)= -2883.973739
G(298 K)= -2884.096972
Lowest Frequency = 19.7337cm-1

Co	5.49941700	7.85456500	21.08455800
O	4.75412800	6.14178200	21.44838200
O	3.89507900	8.60367800	21.78185300
O	3.40202600	4.10343100	22.27685800
O	6.45445500	7.91442400	22.81089300
O	1.37414200	8.95598800	22.19295400
O	0.08480100	7.26230800	24.01327900
O	0.96006600	4.52300800	23.58946600
O	5.08593600	7.10552300	24.40209600
N	6.26920100	9.56429800	20.73810900
N	7.11506900	7.13464800	20.38089100
C	3.44638600	9.77486800	21.47929500
C	2.05738700	10.03640700	21.72195400
C	5.41335700	5.03492500	21.51660800
C	4.71657700	3.87595100	21.99813000
C	7.51904100	5.92804200	20.50774500
H	8.50513000	5.66816100	20.11299600
C	3.63861700	12.10365800	20.71491500
H	4.26071500	12.90304800	20.32072500
C	6.17347800	7.53992600	23.99782000
C	7.69585300	9.45967200	20.46093300
H	8.22199700	9.40921500	21.41938200
H	8.06117700	10.32055400	19.89288700
C	7.39379800	3.59718800	21.26122100
H	8.43048900	3.49252400	20.95198100
C	1.50740200	11.27911700	21.48123700

H	0.45769000	11.46623100	21.67947800
C	4.22413500	10.83770100	20.95319500
C	6.71401200	2.52091600	21.78128800
H	7.20220100	1.56012800	21.90209100
C	5.63234900	10.67119000	20.67001800
H	6.18408400	11.56850500	20.37603900
C	2.60093600	3.01767900	22.70530100
H	2.90043700	2.70307800	23.71357300
H	2.70772300	2.16617800	22.02119600
C	6.76387500	4.85711800	21.11927400
C	5.35799600	2.66350600	22.14715500
H	4.82814700	1.80400500	22.54265200
C	-0.00423500	9.09244900	22.48093800
H	-0.54835300	9.46177200	21.60154800
H	-0.15152000	9.79436600	23.31198900
C	2.30534300	12.32672800	20.97266600
H	1.85896600	13.29796200	20.78937300
C	-0.40227100	4.85877600	23.75162500
H	-0.93872300	4.04806700	24.26659100
H	-0.87798600	4.99414000	22.76902800
C	7.89965500	8.15415400	19.69559900
H	7.49919700	8.25696900	18.68247100
H	8.95762600	7.88030400	19.64266500
C	-0.51669900	6.11831000	24.59128300
H	-1.58191700	6.30763100	24.78921500
H	-0.00829400	5.96927700	25.54709600
C	-0.53947500	7.72499000	22.83267900
H	-1.62777300	7.79634600	22.97234300
H	-0.34042100	7.04234300	21.99589300
C	1.15504700	3.45202700	22.68827700
H	0.86497400	3.75621000	21.67187400
H	0.53727000	2.58945400	22.97752500
C	7.34770500	7.63707700	24.96565100
H	7.00184000	7.61515300	26.00048100
H	7.93037300	8.54279200	24.78215200
H	8.00759100	6.77859400	24.79989500
K	2.77184600	6.67790000	23.22868200
O	0.42229800	4.19891900	18.88765000
C	0.88353500	5.30140600	19.07783500
O	2.16673500	5.55728400	18.92593900
C	2.72240700	6.86206300	19.22919300
C	4.18090900	6.87394100	18.76279600
H	2.67938300	7.02512900	20.30525400
H	2.14096300	7.63414700	18.71601300
O	4.82947100	7.96233600	19.32603300
H	4.62880300	5.91669900	19.07607200
C	4.27366800	6.95910200	17.23937300
H	3.71748700	6.14369800	16.76369700
H	3.86223600	7.91316800	16.88940100
H	5.31924700	6.89671700	16.92256400
O	0.17038600	6.36500300	19.44734700
C	-1.24448300	6.16867300	19.55232900
H	-1.47016500	5.41720500	20.31220900
H	-1.65120000	7.13683900	19.83896900
H	-1.65582300	5.85441800	18.59139000

TS4Co.log

SCF (wB97xd) = -2884.632287
E(SCF)+ZPE(0 K)= -2883.984878
H(298 K)= -2883.940898
G(298 K)= -2884.060373
Lowest Frequency = -147.0039cm-1

K	5.176666	7.777764	21.879327
O	2.891304	8.661219	23.031747
O	3.183259	6.097903	22.396686
O	4.577800	10.454539	22.228662
O	3.133789	6.794124	25.089909
O	4.892761	5.294110	20.630953
O	7.153917	6.867534	20.041872
O	6.682194	9.719115	20.512079
O	5.239416	7.293320	24.462009
N	1.114939	5.472873	24.145734
N	0.851414	7.968128	24.739509
C	3.235316	4.821199	22.220593
C	4.178121	4.320286	21.259825
C	2.954703	9.729768	23.757333
C	3.891981	10.744714	23.370360
C	1.064497	9.115747	25.254900
H	0.358286	9.482320	26.003669
C	2.576299	2.482864	22.622575
H	1.944238	1.776782	23.154500
C	4.388502	6.968874	25.294224
C	0.150989	5.739182	25.216557
H	0.670366	5.629968	26.174471
H	-0.681789	5.031158	25.180702
C	2.313531	11.195196	25.626250
H	1.675408	11.372684	26.487949
C	4.309107	2.967865	21.022543
H	5.032819	2.603033	20.302202
C	2.423156	3.866905	22.884322
C	3.260871	12.123576	25.260802
H	3.397024	13.034955	25.832587
C	1.417325	4.266214	23.835696
H	0.866603	3.459762	24.327099
C	5.492610	11.406129	21.716044
H	6.343593	11.521708	22.399888
H	5.003527	12.380494	21.588951
C	2.146372	9.999195	24.889821
C	4.051279	11.896291	24.114272
H	4.783473	12.641765	23.824014
C	5.844208	4.914352	19.651895
H	5.362304	4.331743	18.856151
H	6.636450	4.308478	20.109950
C	3.503847	2.035720	21.712930
H	3.623258	0.976359	21.514778
C	7.180828	9.212691	19.289575
H	7.863659	9.939909	18.825462
H	6.352479	9.035813	18.587113
C	-0.325391	7.173675	25.048118
H	-1.004509	7.256730	24.193679
H	-0.831168	7.538192	25.947225
C	7.953159	7.934682	19.562741
H	8.479325	7.634473	18.645812
H	8.697512	8.125219	20.340337
C	6.429945	6.169060	19.047517
H	7.096201	5.876807	18.223524
H	5.623589	6.790231	18.632883
C	5.960637	10.924575	20.362772
H	5.094516	10.769995	19.702608
H	6.599355	11.701139	19.917521
C	4.786069	6.762449	26.749206
H	4.498160	7.653338	27.317420
H	5.864708	6.624625	26.837062
H	4.259510	5.908676	27.181222

Co	2.012070	7.062852	23.549245
O	0.778586	7.352669	22.047184
C	0.382208	6.318597	21.176706
C	-0.452451	8.611440	21.850969
H	0.437275	5.361128	21.702749
C	-1.109127	6.627149	20.850145
C	1.217827	6.242815	19.902104
O	-1.495942	7.701728	21.680450
O	-0.537996	9.399632	22.781867
O	0.036117	9.054533	20.608195
H	-1.770700	5.789040	21.077976
H	-1.225019	6.899667	19.793748
H	2.267922	6.057810	20.131952
H	0.849023	5.427871	19.267377
H	1.134274	7.183133	19.350952
C	-0.782479	10.059501	20.032765
H	-0.312938	10.339681	19.088472
H	-0.846371	10.938842	20.683406
H	-1.793663	9.681719	19.833578

XCo.log

SCF (wB97xd) = -2884.016320
E(SCF)+ZPE(0 K)= -2883.971733
H(298 K)= -2883.970788
G(298 K)= -2884.096295
Lowest Frequency = 14.9555cm-1

K	5.30943800	7.57006400	21.97551700
O	2.81738300	8.40748800	22.65492300
O	3.41083100	5.81350800	22.44418300
O	4.46571700	10.23133800	21.83303200
O	2.99100700	6.88297400	25.03234100
O	5.16281300	5.03134900	20.71583900
O	7.45369900	6.59944900	20.36321900
O	6.84853500	9.45441600	20.54678300
O	5.09636500	7.54937600	24.60644900
N	1.19543200	5.21701100	23.99688500
N	0.64174300	7.74069700	24.21149000
C	3.36994400	4.55862500	22.14817300
C	4.34378300	4.06422500	21.21639200
C	2.70198000	9.55934700	23.22655500
C	3.60454900	10.60130900	22.82370700
C	0.69621200	8.96264700	24.58540200
H	-0.10998100	9.35083700	25.21362000
C	2.53096000	2.25309200	22.32266200
H	1.82023100	1.55493200	22.75718300
C	4.16397500	7.25595500	25.36828200
C	0.17897800	5.53220200	24.99453800
H	0.67975300	5.65773000	25.95995800
H	-0.56618700	4.73532500	25.07614000
C	1.70308100	11.18779200	24.77946000
H	0.94265900	11.41220200	25.52293700
C	4.40407000	2.72641900	20.88316100
H	5.15372300	2.36372700	20.18850400
C	2.45092400	3.61981700	22.68226800
C	2.61448700	12.14772600	24.40628900
H	2.59964800	13.13466100	24.85552700
C	1.43477300	4.01529100	23.63135600
H	0.83365800	3.21222700	24.06712000
C	5.39994800	11.18046500	21.35307300
H	6.12200300	11.43261700	22.14082800
H	4.88859700	12.09787500	21.03400300
C	1.73204200	9.89480700	24.20622300
C	3.57129200	11.84893000	23.41236700
H	4.27838000	12.61633300	23.11719900
C	6.17985900	4.65931700	19.80409600
H	5.75387900	4.11791900	18.94940600
H	6.91694500	4.01631100	20.30231400
C	3.48909200	1.80794200	21.44175700
H	3.55031000	0.75991700	21.16977100
C	7.60681800	8.90088500	19.48999700
H	8.36577700	9.61993000	19.14702900
H	6.95369900	8.67461000	18.63410100
C	-0.45438000	6.85530100	24.57448900
H	-1.06931300	6.69994500	23.68169400
H	-1.07404500	7.28047600	25.37000400
C	8.31892800	7.65421400	19.98366200
H	9.01337300	7.31391200	19.20209900
H	8.89845300	7.89914400	20.87743900
C	6.83451300	5.91996500	19.28886400
H	7.57989600	5.63989300	18.53077200
H	6.07566400	6.55224000	18.80703700
C	6.10483000	10.58977700	20.15509400
H	5.36820100	10.31289600	19.38654700
H	6.77014700	11.35722600	19.73336800
C	4.36732000	7.35901500	26.87563900
H	3.91979100	8.29714300	27.22181600
H	5.42932000	7.36704700	27.12668300
H	3.86336500	6.54047200	27.39473500
Co	2.02908800	6.79450400	23.30678700
O	0.90520100	6.66582200	21.81036200
C	-2.28234600	9.49256300	23.22553800
C	-2.26191600	8.53364900	21.16146600
O	-2.82462700	8.51005900	22.50205800
O	-2.64727200	9.80437300	24.32678200
O	-1.27397700	10.07801200	22.57497700
H	-2.95353800	9.12078600	20.54853200
C	-0.95583300	9.29043700	21.40667600
H	-0.68432200	9.96869300	20.59989000
H	-0.13507100	8.60693400	21.65999700
C	1.48021400	6.79766300	20.55171100
H	2.00583000	5.88620000	20.21707400
H	0.68317500	6.99996100	19.81695600
H	2.19828300	7.63129000	20.49699200
C	-2.11462400	7.12743600	20.63363400
H	-3.07826200	6.61263000	20.65093900
H	-1.77278600	7.17167400	19.59532600
H	-1.37191500	6.57702000	21.21902000

VIIICo.log

SCF (wB97xd) = -2502.952251
E(SCF)+ZPE(0 K)= -2502.410639
H(298 K)= -2502.373030
G(298 K)= -2502.479331
Lowest Frequency = 21.2944cm-1

Co	2.442096	6.344059	23.058946
K	5.713810	7.524402	21.987066
O	1.439444	6.130359	21.487682
O	3.109829	8.057529	22.550080
O	3.958439	5.570854	22.194189
O	4.593448	10.086047	21.911253
O	3.322306	6.407161	24.832223
O	5.918727	5.064504	20.584320

O	8.060045	6.874051	20.512321
O	7.134486	9.636346	20.776493
O	5.376540	7.289614	24.586442
N	1.756166	4.646847	23.607653
N	0.927786	7.082343	23.941862
C	4.096120	4.332905	21.866375
C	5.187910	3.989859	20.996794
C	2.815541	9.167805	23.137020
C	3.619196	10.320280	22.835617
C	0.803676	8.294127	24.331972
H	-0.082900	8.563038	24.912740
C	3.541957	1.935107	21.923302
H	2.895863	1.141203	22.289032
C	4.437396	6.849568	25.265036
C	0.661157	4.787428	24.560812
H	1.096014	4.887323	25.560665
H	-0.001756	3.917117	24.544151
C	1.514119	10.618990	24.644046
H	0.680498	10.729510	25.332804
C	5.435207	2.681960	20.634816
H	6.270190	2.436764	19.987753
C	3.270115	3.268830	22.313531
C	2.333096	11.688513	24.369540
H	2.169178	12.651231	24.841067
C	2.156319	3.500884	23.203734
H	1.625135	2.614025	23.560966
C	5.443069	11.153528	21.532295
H	6.072861	11.454333	22.379772
H	4.852537	12.018756	21.204517
C	1.738016	9.356932	24.042679
C	3.394008	11.534519	23.450803
H	4.027855	12.387362	23.234611
C	7.052938	4.842366	19.766779
H	6.773081	4.295386	18.856941
H	7.805531	4.260790	20.315048
C	4.604195	1.639786	21.101535
H	4.812679	0.617021	20.807212
C	8.026190	9.222097	19.760537
H	8.724858	10.034803	19.511676
H	7.467795	8.972904	18.846042
C	-0.084544	6.068395	24.200722
H	-0.651237	5.919675	23.275887
H	-0.767616	6.374186	24.998949
C	8.831180	8.033265	20.254018
H	9.617152	7.811590	19.517855
H	9.309816	8.289496	21.202729
C	7.613286	6.185505	19.360705
H	8.448657	6.017392	18.665949
H	6.838065	6.758839	18.833548
C	6.294294	10.697150	20.371316
H	5.650507	10.373403	19.540303
H	6.894625	11.552379	20.028278
C	4.556277	6.828854	26.785278
H	3.941241	7.636556	27.196335
H	5.590429	6.980634	27.098580
H	4.176452	5.887434	27.189741
C	0.886826	7.252948	20.884644
H	1.639251	7.994897	20.570199
H	0.336656	6.934503	19.984960
H	0.163533	7.784366	21.532032

R-propylene carbonate.log

SCF (wB97xd) = -381.695422
E(SCF)+ZPE(0 K)= -381.591328
H(298 K)= -381.584209
G(298 K)= -381.621660
Lowest Frequency = 95.5474cm-1

C	0.07010100	0.62658600	-0.12389400
O	0.72105600	1.62537100	-0.24635600
O	-1.23059000	0.60348000	0.17912000
O	0.55784500	-0.61147700	-0.27544300
C	-1.73767900	-0.74983300	0.08129400
H	-2.23533700	-0.82796400	-0.88962400
C	-0.44200000	-1.56962900	0.10368100
H	-0.43334100	-2.38596300	-0.61633600
H	-0.20241900	-1.94213700	1.10363400
C	-2.69817500	-1.03375800	1.21120100
H	-3.56736700	-0.37528800	1.15219400
H	-3.04591200	-2.06817300	1.13742800
H	-2.20746500	-0.89625200	2.17862700

IIICo.log

SCF (wB97xd) = -2884.65290204
E(SCF)+ZPE(0 K)= -2884.005598
H(298 K)= -2883.960032
G(298 K)= -2884.086810
Lowest Frequency = 11.4567cm-1

Co	5.473090	8.263367	21.262831
O	4.886063	6.449231	21.389312
O	3.932158	8.770760	22.270229
O	3.767470	4.192118	21.979650
O	6.599140	8.114898	22.871037
O	1.432577	8.844435	22.945681
O	0.507430	6.781733	24.580430
O	1.502809	4.198770	23.658081
O	5.474469	6.907346	24.400799
N	6.096355	10.056529	21.152730
N	7.082133	7.811027	20.329325
C	3.364984	9.924035	22.175881
C	1.991523	10.032284	22.580176
C	5.614712	5.407678	21.195352
C	5.053404	4.126352	21.531906
C	7.574017	6.630446	20.236609
H	8.568214	6.523520	19.794263
C	3.309769	12.346995	21.738406
H	3.831641	13.242037	21.409910
C	6.472825	7.513653	23.990766
C	7.520462	10.122058	20.842204
H	8.068663	9.971390	21.776597
H	7.793503	11.091749	20.415054
C	7.654965	4.201746	20.519543
H	8.662106	4.239581	20.112698
C	1.335466	11.246109	22.572826
H	0.301677	11.316529	22.892764
C	4.003590	11.113176	21.737374
C	7.102941	2.999916	20.890329
H	7.661811	2.075938	20.791235
C	5.388512	11.108784	21.335648
H	5.862870	12.082243	21.185087
C	3.117161	2.992041	22.356114

H	3.581102	2.580872	23.262279
H	3.186050	2.246690	21.553422
C	6.931759	5.412722	20.662413
C	5.785362	2.965749	21.400464
H	5.360163	2.010188	21.686609
C	0.103327	8.836623	23.432566
H	-0.578159	9.305012	22.710626
H	0.047569	9.383989	24.382492
C	1.998817	12.418215	22.147261
H	1.466994	13.363347	22.148729
C	0.148870	4.409270	24.006702
H	-0.279907	3.494912	24.443262
H	-0.438398	4.653008	23.109288
C	7.834059	8.979294	19.878636
H	7.510178	9.240127	18.865637
H	8.907866	8.773561	19.850008
C	0.057335	5.519716	25.038035
H	-0.984642	5.589108	25.382863
H	0.689096	5.274447	25.895546
C	-0.321671	7.398100	23.615247
H	-1.371064	7.383604	23.942989
H	-0.255706	6.877402	22.649849
C	1.654734	3.288398	22.588369
H	1.208455	3.705358	21.673579
H	1.147398	2.339746	22.816653
C	7.728754	7.566631	24.853606
H	7.506053	7.282921	25.883482
H	8.175901	8.563252	24.826615
H	8.463272	6.863684	24.446235
K	3.066730	6.537746	23.383357
O	6.379530	6.995014	15.362414
C	6.228508	8.193353	15.393162
O	5.681028	8.879362	16.383129
C	5.220364	8.137010	17.533183
C	4.699293	9.128167	18.576903
H	6.052540	7.555981	17.937084
H	4.426392	7.451252	17.219781
O	4.392837	8.426079	19.727464
H	5.486892	9.884570	18.733037
C	3.459789	9.872176	18.075870
H	3.660492	10.407299	17.141369
H	2.641785	9.162326	17.905635
H	3.140128	10.593157	18.834491
O	6.619727	8.965543	14.384232
C	6.424248	10.386148	14.451234
H	6.974791	10.809859	15.293104
H	6.817549	10.770247	13.511845
H	5.362889	10.623918	14.542391

IVCo.log

SCF (wB97xd) = -3073.24143897
E(SCF)+ZPE(0 K)= -3072.580942
H(298 K)= -3072.531563
G(298 K)= -3072.665935
Lowest Frequency = 13.3691cm-1

Co	5.413397	7.959583	21.058208
O	4.869096	6.154063	21.331135
O	3.735179	8.482488	21.809280
O	3.804392	3.963216	22.211099
O	6.404113	8.036875	22.764424
O	1.274061	8.675608	22.666908
O	0.550428	6.778809	24.629137
O	1.539812	4.117004	23.877662
O	5.249572	6.943870	24.354140
N	5.998440	9.753879	20.797458
N	7.078709	7.450894	20.288408
C	3.211217	9.659330	21.741625
C	1.864543	9.825823	22.211905
C	5.659214	5.134860	21.380565
C	5.122925	3.896458	21.871675
C	7.622366	6.296410	20.362539
H	8.619595	6.165316	19.933592
C	3.214360	12.073043	21.253950
H	3.751863	12.944565	20.889451
C	6.247783	7.542764	23.930021
C	7.411602	9.816831	20.443894
H	7.990841	9.789639	21.372354
H	7.650125	10.734380	19.897679
C	7.791834	3.949582	21.055452
H	8.825535	3.976165	20.720676
C	1.244760	11.058900	22.190206
H	0.229534	11.172148	22.552848
C	3.876053	10.823297	21.270071
C	7.257895	2.789873	21.566410
H	7.858606	1.891038	21.651797
C	5.262876	10.797115	20.861444
H	5.712852	11.761457	20.609385
C	3.172861	2.804636	22.725689
H	3.627223	2.520683	23.683950
H	3.272596	1.967171	22.023136
C	7.013272	5.127578	20.956612
C	5.906731	2.765539	21.974351
H	5.494014	1.842988	22.367294
C	0.085392	8.804207	23.433029
H	-0.707180	9.283154	22.844435
H	0.286431	9.415038	24.322115
C	1.919163	12.195786	21.700408
H	1.414190	13.155298	21.689994
C	0.185518	4.383838	24.184860
H	-0.280799	3.501873	24.648484
H	-0.372620	4.611935	23.264718
C	7.715723	8.573490	19.613384
H	7.256040	8.669901	18.624973
H	8.792693	8.417637	19.499894
C	0.120435	5.539238	25.168403
H	-0.906523	5.626070	25.549422
H	0.784351	5.331360	26.011538
C	-0.411172	7.432638	23.828595
H	-1.348951	7.565347	24.387119
H	-0.641040	6.841138	22.932346
C	1.703002	3.104102	22.905730
H	1.264340	3.424014	21.949509
H	1.199606	2.180449	23.225837
C	7.458750	7.718000	24.839109
H	7.183066	7.580843	25.886065
H	7.916243	8.699434	24.694711
H	8.204886	6.961438	24.572774
K	2.947191	6.416733	23.216438
O	0.685222	8.848049	16.535150
C	0.742911	7.690635	16.873866
O	1.673819	7.155733	17.649320
C	2.745626	8.018782	18.092084
C	3.723125	7.192130	18.925374
H	2.324350	8.830153	18.692072

H	3.243828	8.440223	17.213165
O	4.706896	8.083295	19.323877
H	3.173764	6.789991	19.789334
C	4.292275	6.014523	18.130341
H	3.507705	5.314314	17.822879
H	4.806094	6.379517	17.232479
H	5.016727	5.476474	18.747514
O	-0.170700	6.807771	16.482014
C	-0.035192	5.425228	16.842585
H	-0.084893	5.299756	17.925529
H	-0.878425	4.925512	16.369579
H	0.905565	5.020505	16.465272
C	0.429394	7.090009	20.444382
O	-0.052182	8.046948	20.006571
O	0.887716	6.114805	20.873832

TS2Co.log

SCF (wB97xd) = -3073.22996851
E(SCF)+ZPE(0 K)= -3072.568999
H(298 K)= -3072.520856
G(298 K)= -3072.652366
Lowest Frequency = -219.3148cm-1

Co	5.378902	7.360410	21.246756
O	4.760552	5.779841	22.096664
O	3.944270	8.346398	22.024222
O	3.582400	3.988875	23.539387
O	6.576425	7.805142	22.688671
O	1.499043	8.853481	22.768837
O	0.523334	7.552449	25.025248
O	1.367802	4.747970	25.105490
O	5.401108	7.377791	24.563476
N	6.036717	8.912380	20.359386
N	6.902205	6.480339	20.477551
C	3.382450	9.362594	21.464037
C	2.047586	9.710406	21.866683
C	5.433078	4.696746	22.287852
C	4.836288	3.680776	23.108596
C	7.354918	5.334253	20.833818
H	8.310871	5.009972	20.414656
C	3.335790	11.345283	19.996786
H	3.848129	11.974745	19.273975
C	6.419586	7.724910	23.961594
C	7.442661	8.774890	19.995826
H	8.038579	8.977219	20.890268
H	7.722837	9.479571	19.207306
C	7.373649	3.203032	22.039624
H	8.353085	3.019908	21.605930
C	1.416951	10.826764	21.356265
H	0.415907	11.088575	21.680574
C	4.005551	10.207516	20.508974
C	6.795889	2.272140	22.868336
H	7.309164	1.348072	23.110126
C	5.363804	9.972192	20.096684
H	5.853886	10.763124	19.523357
C	2.883904	3.052406	24.340038
H	3.351252	2.981685	25.330738
H	2.893884	2.060435	23.870821
C	6.708311	4.417148	21.736014
C	5.511405	2.515028	23.404064
H	5.064251	1.768358	24.050833
C	0.173338	9.075361	23.213172
H	-0.496273	9.259070	22.363421
H	0.142472	9.942635	23.885206
C	2.060881	11.651257	20.407099
H	1.544036	12.521435	20.017658
C	0.035931	5.153444	25.353580
H	-0.434044	4.487322	26.092433
H	-0.556481	5.090307	24.428874
C	7.652822	7.330043	19.554178
H	7.261761	7.187129	18.541577
H	8.714342	7.067934	19.549751
C	0.029599	6.565474	25.912850
H	-0.996874	6.811815	26.221229
H	0.675163	6.611108	26.793536
C	-0.307821	7.825398	23.914808
H	-1.345610	7.987726	24.239935
H	-0.298887	6.988675	23.202624
C	1.444636	3.496951	24.453044
H	0.997117	3.567505	23.450716
H	0.895290	2.732738	25.021931
C	7.679391	8.092140	24.734772
H	7.456537	8.220619	25.794886
H	8.126779	9.004208	24.332310
H	8.411980	7.286489	24.620713
K	2.920182	6.691451	23.779982
O	6.410370	4.184007	16.169640
C	6.395401	5.370126	15.944737
O	5.748644	6.283583	16.654564
C	5.000450	5.824539	17.801960
C	4.399919	7.083577	18.443581
H	5.677151	5.320311	18.493816
H	4.241514	5.115582	17.467603
O	4.154607	6.915011	19.812754
H	5.148587	7.875116	18.321154
C	3.130920	7.540189	17.728679
H	3.327832	7.703309	16.664128
H	2.340572	6.786517	17.817870
H	2.775422	8.475924	18.169355
O	7.060079	5.891736	14.920025
C	7.028514	7.308818	14.689970
H	7.449074	7.844759	15.542869
H	7.643580	7.465180	13.805821
H	6.006821	7.644757	14.504571
C	2.873275	5.628489	20.246989
O	2.072195	6.192715	20.915966
O	3.210498	4.629511	19.693941

VCo.log

SCF (wB97xd) = -3073.23223368
E(SCF)+ZPE(0 K)= -3072.569338
H(298 K)= -3072.521240
G(298 K)= -3072.652354
Lowest Frequency = 12.0094cm-1

Co	5.359347	7.348455	21.239836
O	4.757415	5.766302	22.080436
O	3.945499	8.350595	22.021198
O	3.598084	3.981591	23.555842
O	6.559477	7.797974	22.630315
O	1.513388	8.879603	22.816121
O	0.565559	7.565279	25.077028
O	1.402738	4.750100	25.148487
O	5.355127	7.372185	24.489622

N	5.996085	8.887243	20.311429
N	6.861107	6.457587	20.434863
C	3.372025	9.363120	21.462971
C	2.048170	9.721096	21.893140
C	5.431883	4.682949	22.274123
C	4.846112	3.674260	23.110669
C	7.323850	5.313433	20.789288
H	8.272955	4.991438	20.353970
C	3.300438	11.330286	19.973222
H	3.799274	11.948008	19.231344
C	6.377546	7.725470	23.907512
C	7.399467	8.749270	19.935954
H	8.003569	8.958557	20.823167
H	7.670220	9.449719	19.140714
C	7.370829	3.189838	22.008832
H	8.343896	3.004776	21.562176
C	1.413939	10.836473	21.385031
H	0.422979	11.108729	21.730589
C	3.975216	10.193979	20.483836
C	6.805415	2.266222	22.853472
H	7.322639	1.344612	23.095955
C	5.322700	9.948952	20.050728
H	5.807674	10.731157	19.461884
C	2.912053	3.051487	24.375920
H	3.393454	2.992334	25.360482
H	2.917849	2.055134	23.916307
C	6.698101	4.400285	21.706538
C	5.528296	2.512081	23.404969
H	5.091295	1.770005	24.063758
C	0.198674	9.114192	23.288124
H	-0.485390	9.310823	22.453218
H	0.192556	9.977538	23.965595
C	2.038382	11.647055	20.411110
H	1.516116	12.515392	20.025005
C	0.073485	5.164660	25.397457
H	-0.401619	4.498710	26.133144
H	-0.518690	5.110066	24.471996
C	7.604182	7.303584	19.499576
H	7.204200	7.155524	18.491914
H	8.664981	7.039904	19.487550
C	0.077484	6.574228	25.963838
H	-0.944823	6.822426	26.284064
H	0.732011	6.612198	26.838219
C	-0.286926	7.865653	23.990270
H	-1.313290	8.044317	24.341964
H	-0.312810	7.036010	23.270140
C	1.473138	3.494421	24.504006
H	1.013272	3.556745	23.506683
H	0.932264	2.733444	25.085132
C	7.623624	8.117499	24.687228
H	7.389487	8.230446	25.746396
H	8.046752	9.045492	24.295359
H	8.378219	7.333487	24.569148
K	2.860225	6.649261	23.685698
O	6.390828	4.193110	16.088450
C	6.459792	5.390497	15.957208
O	5.810339	6.286124	16.691031
C	4.953746	5.781842	17.740422
C	4.323794	7.029598	18.368296
H	5.557133	5.236720	18.467600
H	4.217958	5.105908	17.306501
O	4.052883	6.869989	19.761649
H	5.073427	7.820697	18.318753
C	3.080461	7.508477	17.633618
H	3.324747	7.697239	16.584069
H	2.286382	6.756279	17.670135
H	2.716410	8.437029	18.081485
O	7.228330	5.948218	15.030163
C	7.293764	7.378266	14.912951
H	7.686600	7.820486	15.830512
H	7.975733	7.562388	14.085138
H	6.308244	7.790831	14.690920
C	2.962828	5.854710	20.095196
O	2.134336	6.298623	20.870322
O	3.107438	4.795297	19.498745

VICo-VIK.log

SCF (wB97xd) = -3072.601948
E(SCF)+ZPE(0 K)= -3072.555104
H(298 K)= -3072.554159
G(298 K)= -3072.683798
Lowest Frequency = 20.1376cm-1

Co	2.48259600	6.27956500	23.32784000
K	5.42837500	7.57360600	21.62608500
O	2.91309400	7.95123300	22.52816700
O	3.96652200	5.52878800	22.39918700
O	4.08447900	9.98856800	21.43508900
O	3.46660600	6.64355600	24.92802900
O	5.75286100	4.98657100	20.60442700
O	7.70596800	6.92521400	20.08932600
O	6.54552000	9.61522000	20.10277000
O	5.38594200	7.62027400	24.26743300
N	2.02111100	4.62032900	24.13748800
N	0.97403800	6.98976300	24.23777800
C	4.17832900	4.26958400	22.18785800
C	5.17431800	3.91618700	21.21839100
C	2.55098000	9.10654200	22.97586700
C	3.18756600	10.26860900	22.42188300
C	0.75957100	8.22996600	24.47176400
H	-0.09708800	8.49320100	25.09746400
C	3.83777700	1.86474600	22.56203000
H	3.31130600	1.07344400	23.08867200
C	4.59952000	7.21687900	25.12630200
C	1.01402600	4.79174800	25.18037900
H	1.52986600	5.06823100	26.10545500
H	0.45009300	3.86911600	25.34273600
C	1.23680100	10.63515300	24.38884000
H	0.46211800	10.76896400	25.13910800
C	5.47918700	2.59532300	20.95716000
H	6.23928100	2.33910800	20.22756500
C	3.50757300	3.21073500	22.84589600
C	1.90368100	11.72278600	23.87725100
H	1.67762300	12.72604400	24.22120100
C	2.48756100	3.46866700	23.83506500
H	2.08402000	2.59898100	24.35898600
C	4.76920200	11.05966800	20.80846500
H	5.43835800	11.55009900	21.52742200
H	4.05522100	11.79987300	20.42507800
C	1.54475800	9.32375200	23.95193300
C	2.88402500	11.53460500	22.87828000
H	3.39228200	12.40254000	22.47311800
C	6.79504300	4.75576500	19.67259100
H	6.44923200	4.10309500	18.86063700
H	7.64889000	4.28272500	20.17407900

C	4.80675600	1.55716300	21.63535600
H	5.05979100	0.52547200	21.41772600
C	7.39170900	9.15019600	19.06885800
H	7.98980200	9.98008600	18.66368700
H	6.78749000	8.74069400	18.24804700
C	0.10396300	5.93082200	24.73548200
H	-0.52112700	5.58901500	23.90478000
H	-0.53066700	6.28449500	25.55312400
C	8.34125700	8.10442600	19.62579700
H	9.08215400	7.85697200	18.85207900
H	8.86873100	8.51477100	20.49058600
C	7.20102900	6.08247700	19.07388800
H	7.96712900	5.89762400	18.30757300
H	6.33367000	6.54310000	18.58337800
C	5.55270100	10.51032500	19.63927200
H	4.88063700	9.99408000	18.93961300
H	6.01780000	11.35688000	19.11323600
C	4.91644700	7.39907600	26.60365900
H	4.29058500	8.20500100	27.00061300
H	5.96527300	7.66547400	26.74101400
H	4.68204100	6.49150600	27.16474400
O	1.40470900	5.98469200	21.75058500
C	0.73281200	5.01685800	21.27493100
O	0.12657700	4.12292800	21.85069500
O	0.66398400	5.00806800	19.90062400
C	1.31995400	6.04783700	19.16432600
H	1.15871700	7.00290100	19.67252700
C	0.69488700	6.06977000	17.78143900
C	2.82332100	5.76345600	19.15093500
H	-0.37582200	6.27600300	17.85543000
H	1.15000300	6.85534200	17.17159900
H	0.83768200	5.11065800	17.27466200
H	3.06071300	4.89048700	18.53888700
H	3.18759000	5.63250600	20.16739500
O	3.52298900	6.91834900	18.65094700
C	4.01448700	6.87727500	17.42090200
O	3.96089700	5.94851700	16.65135900
O	4.60078100	8.04514100	17.17733900
C	5.20604400	8.19258700	15.88657300
H	5.60909600	9.20359600	15.87372600
H	6.00743200	7.46188200	15.75690500
H	4.45928700	8.07035900	15.10008300

TS3Co.log

SCF (wB97xd) = -3073.214560
E(SCF)+ZPE(0 K)= -3072.552290
H(298 K)= -3072.505172
G(298 K)= -3072.631852
Lowest Frequency = -601.5359cm-1

Co	2.258477	6.742741	23.647852
K	4.938878	7.815133	21.443496
O	2.853871	8.435508	23.013626
O	3.387497	5.911222	22.359737
O	4.408707	10.376487	22.167369
O	3.526068	6.706509	25.050756
O	5.052280	5.254609	20.452614
O	7.407175	6.834648	20.428001
O	6.851845	9.715441	20.851017
O	5.451018	7.342942	24.061691
N	1.609914	5.080021	24.325498
N	1.042367	7.540909	24.873181
C	3.467142	4.647616	22.089734
C	4.363763	4.237453	21.047588
C	2.834077	9.530186	23.703882
C	3.668621	10.621980	23.283794
C	1.097235	8.744229	25.306467
H	0.386367	9.043386	26.080807
C	2.893456	2.269315	22.375956
H	2.323037	1.511843	22.906664
C	4.780657	7.012594	25.036909
C	0.780441	5.258461	25.514654
H	1.443161	5.308382	26.384769
H	0.082967	4.426098	25.642664
C	2.049679	10.985878	25.535160
H	1.408131	11.117651	26.402183
C	4.491999	2.908053	20.697999
H	5.170027	2.614041	19.904710
C	2.742949	3.625251	22.753024
C	2.880144	11.999710	25.122595
H	2.915215	12.943610	25.655188
C	1.864937	3.915833	23.858123
H	1.394663	3.057397	24.344239
C	5.357181	11.349428	21.754108
H	6.078228	11.533408	22.561072
H	4.852618	12.291760	21.505185
C	2.018738	9.751042	24.844241
C	3.691969	11.811848	23.984695
H	4.337213	12.622298	23.665417
C	6.191719	4.917873	19.671811
H	5.888917	4.391969	18.757372
H	6.854466	4.266596	20.255563
C	3.749291	1.910686	21.362239
H	3.866128	0.872539	21.072017
C	7.579652	9.202707	19.747783
H	8.342678	9.929540	19.430006
H	6.896181	9.023760	18.906615
C	0.050636	6.584383	25.348979
H	-0.725198	6.490310	24.582620
H	-0.407202	6.914214	26.285786
C	8.280144	7.921745	20.167929
H	9.005643	7.645126	19.389492
H	8.825549	8.096466	21.099347
C	6.916439	6.182042	19.271462
H	7.749100	5.895774	18.612534
H	6.241617	6.840825	18.712732
C	6.067994	10.845358	20.519540
H	5.340013	10.577459	19.741301
H	6.707309	11.656114	20.139761
C	5.403963	6.955686	26.423560
H	5.079903	7.835222	26.989428
H	6.492278	6.963397	26.351762
H	5.067999	6.069425	26.966381
O	0.760373	6.700999	22.317448
C	1.029418	6.785379	21.096308
O	1.169167	5.833475	20.288390
O	1.175877	8.011745	20.580047
C	1.425031	7.976428	19.162878
H	2.113102	8.800621	18.990299
C	0.131904	8.159020	18.389605
C	2.125558	6.662591	18.853236
H	-0.560579	7.335264	18.586549
H	-0.344431	9.100227	18.672863
H	0.351439	8.186028	17.319181
H	1.711126	5.967329	18.139996

H	3.058218	6.412663	19.341425
O	3.155678	7.373617	17.333127
C	4.162560	8.076355	17.658315
O	4.485058	8.439414	18.793522
O	4.982001	8.471597	16.649830
C	4.659509	8.062676	15.322152
H	3.684690	8.451907	15.017860
H	5.440940	8.482010	14.688370
H	4.656344	6.972993	15.237960

IXCo.log

SCF (wB97xd) = -3072.593243
E(SCF)+ZPE(0 K)= -3072.546377
H(298 K)= -3072.545432
G(298 K)= -3072.673336
Lowest Frequency = 25.4349cm-1

Co	2.15022600	6.52772500	23.87928400
K	4.75224000	7.50302400	21.49983200
O	2.66464500	8.16055100	23.06790100
O	3.27816100	5.61949300	22.65540300
O	4.02190000	10.07893800	21.93581500
O	3.44050400	6.67164800	25.24204000
O	4.77471400	4.88458500	20.67988200
O	6.78519700	6.60473900	19.74288100
O	6.13426200	9.48908100	20.11001300
O	5.31890100	7.27850900	24.14906200
N	1.57824500	4.90833100	24.71880100
N	0.92429500	7.38621000	25.05809200
C	3.32416700	4.34558000	22.43779600
C	4.14597900	3.88693400	21.35256100
C	2.57000000	9.32694600	23.62067300
C	3.30908600	10.41412200	23.04131900
C	0.92518700	8.63275300	25.35542700
H	0.22979100	8.97549600	26.12548100
C	2.76622400	1.98590800	22.86581800
H	2.22981500	1.25507800	23.46484500
C	4.68292700	7.02124400	25.16722300
C	0.81969300	5.16816000	25.93927500
H	1.53422800	5.33449400	26.75176100
H	0.17413000	4.32355900	26.19533000
C	1.72289800	10.94118400	25.28300900
H	1.09078800	11.13578300	26.14512200
C	4.25391500	2.54362000	21.05660000
H	4.88258700	2.21233200	20.23750400
C	2.63742500	3.35975100	23.18838700
C	2.47286000	11.94874900	24.72790400
H	2.45547900	12.95034300	25.14305400
C	1.82514600	3.71458500	24.32290900
H	1.39950100	2.88840900	24.89780300
C	4.74736000	11.08746200	21.24918600
H	5.60086900	11.41343500	21.85813200
H	4.10108800	11.95195800	21.05105600
C	1.76405800	9.63027300	24.74749600
C	3.27120600	11.67793000	23.59637300
H	3.85816100	12.48184300	23.16691100
C	5.55196100	4.56975100	19.53651700
H	4.96045600	3.97891600	18.82496600
H	6.43781500	3.99301200	19.83238500
C	3.55633800	1.58047600	21.81755300
H	3.65545900	0.52947100	21.56985100
C	6.54113600	8.91081200	18.88260200
H	7.11304400	9.64396900	18.29265600
H	5.65405900	8.61827100	18.30482200
C	0.01566400	6.43764100	25.69450700
H	-0.80816800	6.22944600	25.00360200
H	-0.39563800	6.84146200	26.62367900
C	7.43751700	7.71435600	19.15028200
H	7.90583700	7.41218800	18.20194300
H	8.22938700	8.00100500	19.84779800
C	5.95051200	5.86525000	18.86652200
H	6.49232000	5.62085600	17.94109700
H	5.04702700	6.43672000	18.61406000
C	5.19892300	10.53553300	19.91823100
H	4.32953200	10.15184100	19.36852600
H	5.66093700	11.35573300	19.34784300
C	5.33317500	7.12214800	26.53850900
H	4.99228200	8.04422000	27.02044300
H	6.41909900	7.15494000	26.44237700
H	5.03529600	6.28525200	27.17388700
O	0.65504600	6.23520000	22.57588400
C	-0.03422400	7.03702500	21.96120500
O	-0.97923100	6.62658800	21.14187900
O	0.04651800	8.33498900	22.06727300
C	-0.83173400	8.97074100	21.09319500
H	-0.16674200	9.38694400	20.33181900
C	-1.65960100	10.02677100	21.78580100
C	-1.61951900	7.76889800	20.53873300
H	-2.27217100	9.58794600	22.57892400
H	-1.01320600	10.79628300	22.21455100
H	-2.32030900	10.50195200	21.05506200
H	-2.66756200	7.76254400	20.84460600
H	-1.52690700	7.67089300	19.45859500
O	1.65509800	9.43185200	19.03893600
C	2.17399400	8.30648300	19.14571300
O	3.37488800	7.97901100	19.10064100
O	1.24465200	7.28626300	19.34441200
C	1.75372900	5.96160200	19.43139400
H	2.41153900	5.84712900	20.29581100
H	0.88286700	5.31279500	19.54317500
H	2.30651800	5.68935200	18.52688800

VIICo.log

SCF (wB97xd) = -3265.613889
E(SCF)+ZPE(0 K)= -3265.561354
H(298 K)= -3265.560410
G(298 K)= -3265.701869
Lowest Frequency = 13.6213cm-1

Co	4.56196400	6.82226800	20.99089300
O	3.58400700	5.32444900	21.64870200
O	3.41499600	7.97335500	21.95916600
O	2.36362400	3.73534300	23.35602200
O	5.90587600	6.73108100	22.31162200
O	1.50743600	9.07372100	23.31819500
O	0.63646900	7.77223400	25.65632800
O	0.74805600	4.81620500	25.43922700
O	4.75634200	6.38700800	24.21923300
N	5.54086500	8.26691400	20.21572600
N	5.66004400	5.70290200	19.91035800
C	3.32596500	9.26154600	21.85374600
C	2.28691700	9.91775100	22.59565800
C	4.11846100	4.15461900	21.84519200
C	3.49811400	3.25903400	22.77519100

C	5.91123700	4.46942400	20.14664600
H	6.66537100	3.97021600	19.53368800
C	3.99708500	11.48257500	21.03276900
H	4.66953700	12.08149600	20.42477200
C	5.79766300	6.51422300	23.58070200
C	6.71437200	7.79746900	19.48268000
H	7.52853600	7.65681100	20.20090200
H	7.02532600	8.51991000	18.72338100
C	5.79917100	2.39766500	21.44001200
H	6.68089200	2.06655100	20.89843900
C	2.13238000	11.28824700	22.54432500
H	1.34568800	11.77219400	23.11240700
C	4.17648100	10.07717000	21.06869700
C	5.20930200	1.58321400	22.37714300
H	5.61762300	0.60367800	22.59969300
C	5.26469600	9.51507400	20.31117000
H	5.91003200	10.22200600	19.78399800
C	1.69491400	2.92528900	24.31245000
H	2.34940400	2.74622700	25.17486300
H	1.41953700	1.96166300	23.86485000
C	5.26882800	3.67896900	21.16656400
C	4.04561200	2.01779700	23.03983200
H	3.57834600	1.35654100	23.76012700
C	0.48102300	9.60802400	24.13955700
H	-0.23209900	10.18263500	23.53436000
H	0.91808200	10.26736900	24.90065900
C	2.99359200	12.08104400	21.75534500
H	2.85548700	13.15622900	21.72946900
C	-0.40516600	5.53901000	25.84526700
H	-0.97975800	4.94590100	26.57336300
H	-1.04943600	5.73651300	24.97628300
C	6.34048200	6.45638000	18.86334800
H	5.63939300	6.60991600	18.03646300
H	7.21879600	5.92215400	18.49113000
C	0.02194800	6.83113000	26.51872200
H	-0.86002700	7.27968800	26.99914400
H	0.76100700	6.61196100	27.29435300
C	-0.25359500	8.45710100	24.78922300
H	-1.09750000	8.86984500	25.36117500
H	-0.64708800	7.78065700	24.01829900
C	0.42960200	3.62891000	24.74593100
H	-0.19174900	3.85926600	23.86940300
H	-0.13633700	2.94411500	25.39481100
C	7.15009300	6.38623600	24.26318500
H	7.04462400	6.50738100	25.34199600
H	7.85975700	7.11576300	23.86756800
H	7.54842400	5.38702600	24.05817900
K	2.11270100	6.42678300	23.65145900
O	-2.24815400	5.93186300	22.96107200
C	-1.31391300	5.68403500	22.18491900
O	-0.14234100	6.10882400	22.19524800
C	1.87620800	6.96254900	19.61132600
C	2.54724000	5.86987400	18.90694200
H	1.53764500	6.80800100	20.63184200
H	1.38457500	7.74743700	19.04325000
O	3.31338000	6.98159000	19.44889300
H	2.69070000	4.95811600	19.47753900
C	2.56859200	5.74640200	17.41471700
H	1.74487100	5.09758500	17.10252700
H	2.45130300	6.72098600	16.93406900
H	3.50415600	5.28936100	17.08026800
C	-0.68713400	4.43255800	20.20076200
H	-0.04955700	5.29656600	20.00079200
O	-1.66653900	4.78528500	21.16923400
C	-1.41303800	4.01036300	18.93549700
C	0.17759500	3.31907900	20.78339900
H	-2.00365400	4.84213400	18.54283800
H	-0.69134400	3.70673100	18.17200800
H	-2.08307100	3.16808700	19.13686800
H	-0.40308800	2.40195800	20.91663800
H	0.61211100	3.63345700	21.73444600
O	1.25264600	3.08695700	19.85913400
C	1.90019200	1.93433000	19.94936100
O	1.62951600	1.02128900	20.69224700
O	2.89401900	1.95313700	19.07049800
C	3.70205300	0.77200200	19.00721200
H	4.48407200	0.99765700	18.28463800
H	4.13904000	0.56108700	19.98463100
H	3.10492200	-0.07722500	18.66788900

TS1Co*.log

SCF (wB97xd) = -3265.583489
E(SCF)+ZPE(0 K)= -3265.531809
H(298 K)= -3265.530865
G(298 K)= -3265.670705
Lowest Frequency = -550.9013cm-1

Co	4.63241500	7.04714000	21.11782600
O	3.59510800	5.56972800	21.74803400
O	3.58092800	8.18793600	22.21416900
O	2.31926300	3.94934200	23.34440300
O	6.00631700	6.76980100	22.43156900
O	1.60157000	9.27462200	23.47929400
O	0.43632200	7.89736300	25.64473300
O	0.49676300	4.96338700	25.26711200
O	4.90796600	6.39256500	24.36174000
N	5.69299100	8.48300600	20.44727400
N	5.65653300	5.94231700	19.95159900
C	3.48579600	9.47254500	22.09985500
C	2.42254000	10.12647100	22.80813800
C	4.02622000	4.34530700	21.78487600
C	3.36419400	3.41785200	22.65447800
C	5.77983900	4.67184400	20.03957100
H	6.46932700	4.17164600	19.35494700
C	4.19359600	11.69960300	21.32750900
H	4.88617100	12.30223500	20.74601700
C	5.93235400	6.47974400	23.68164100
C	6.84332900	7.99653400	19.69295000
H	7.63577000	7.74766000	20.40602300
H	7.21202400	8.75178000	18.99334000
C	5.45007200	2.47186000	21.05737600
H	6.25244400	2.11176200	20.41929400
C	2.28253600	11.49929400	22.77895600
H	1.48298900	11.98224300	23.32954300
C	4.35991900	10.29323200	21.34429400
C	4.81570600	1.61672600	21.92685100
H	5.10768300	0.57468900	21.99580400
C	5.45266400	9.73263200	20.58814000
H	6.12508800	10.44229500	20.09933000
C	1.51948500	3.10287100	24.15605700
H	2.06423200	2.83772300	25.07099300
H	1.26636100	2.17989900	23.61882400
C	5.07739200	3.83454100	20.98261700
C	3.76524200	2.09893500	22.73245900
H	3.26765900	1.41363800	23.40882500

C	0.51294000	9.80951900	24.21448500
H	-0.11537900	10.43422300	23.56654000
H	0.88716400	10.42040600	25.04619700
C	3.17631800	12.29710000	22.03276400
H	3.05007400	13.37405500	22.02213300
C	-0.67757600	5.69617900	25.56795000
H	-1.35964200	5.08185000	26.17551000
H	-1.19192600	5.96443300	24.63483600
C	6.38924200	6.73306100	18.96994200
H	5.69962200	6.99627800	18.16171400
H	7.23511300	6.17664000	18.55661500
C	-0.30717000	6.93217500	26.36804200
H	-1.22889700	7.38403200	26.76251200
H	0.32236400	6.64179900	27.21358800
C	-0.32614600	8.66183300	24.72578100
H	-1.21191800	9.08124500	25.22481300
H	-0.65303800	8.04317300	23.88016000
C	0.23136500	3.82582400	24.46957700
H	-0.25821700	4.13020900	23.53387700
H	-0.43115800	3.13067000	25.00706700
C	7.29152000	6.19928200	24.30707600
H	7.23380900	6.26108200	25.39476300
H	8.04690700	6.89141100	23.92870600
H	7.59925400	5.18559200	24.02910900
K	2.27545200	6.61734400	23.90551200
O	-0.43387600	6.42669200	22.22277600
C	-0.47878900	5.84497400	21.13739200
O	-0.11035700	6.26921400	20.00531800
C	1.66033900	7.21080200	20.01755300
C	2.54634800	6.41074000	19.18182600
H	1.71913200	7.10872100	21.08949800
H	1.24943000	8.12145400	19.60378700
O	3.43972900	7.41057500	19.64452100
H	2.77324400	5.41828500	19.57367500
C	2.35653600	6.40734600	17.68577700
H	1.52643800	5.74589700	17.41990900
H	2.13941000	7.41453400	17.31788200
H	3.26357900	6.04000000	17.19653300
C	-0.54128800	3.65205300	20.16249300
H	-0.50508900	4.16083000	19.19550600
O	-0.99716400	4.56315600	21.16847300
C	-1.50127600	2.48024800	20.12082900
C	0.87461000	3.26073100	20.58322200
H	-2.49679300	2.81205800	19.81543700
H	-1.15083600	1.73694600	19.39860700
H	-1.57129200	2.00263700	21.10312100
H	0.84279900	2.60595300	21.45494000
H	1.45077300	4.15537500	20.82541000
O	1.58393200	2.61366600	19.52108600
C	1.92782800	1.33972900	19.68693300
O	1.57048300	0.61181000	20.58109200
O	2.73093200	1.00012800	18.68853400
C	3.22954500	-0.34258100	18.71480900
H	3.85346300	-0.43514900	17.82820200
H	3.82351500	-0.50460600	19.61688700
H	2.40512700	-1.05725500	18.67690900

IK.log

SCF (wB97xd) = -2884.65871762
E(SCF)+ZPE(0 K)= -2884.012769
H(298 K)= -2883.966741
G(298 K)= -2884.091051

Lowest Frequency = 27.1392cm-1

Co	0.16394800	-0.29817200	-0.24264700
O	0.11457800	-0.14542000	1.66011000
O	2.05635100	-0.29930800	-0.12927900
O	0.60816800	-0.32196900	4.19929000
O	-0.00377500	-2.21105700	-0.21175500
O	4.55390400	-0.16231800	0.53346100
O	5.39143100	-0.92460900	3.12122000
O	3.27060200	-0.52981800	5.14605400
O	1.35990700	-2.96391100	1.41512300
N	0.14920500	-0.48586100	-2.14044700
N	-1.73652500	-0.25415200	-0.38312000
C	2.89503400	-0.41498300	-1.10550000
C	4.29333100	-0.35575800	-0.78931600
C	-0.88293100	-0.50296200	2.39950100
C	-0.65868200	-0.63343500	3.81259000
C	-2.56360600	-0.51113100	0.55821000
H	-3.62924400	-0.53729700	0.31557600
C	3.55326600	-0.72469700	-3.45522000
H	3.25365700	-0.86159700	-4.49095000
C	0.51631000	-3.12069200	0.52966700
C	-1.20163400	-0.66309900	-2.67412900
H	-1.42936400	-1.73428600	-2.67156700
H	-1.27637300	-0.28519600	-3.69772100
C	-3.22416800	-1.13843800	2.83367100
H	-4.22409200	-1.31176800	2.44483300
C	5.25556900	-0.48546100	-1.77033200
H	6.30900400	-0.44425900	-1.51653100
C	2.54894400	-0.59389900	-2.46679400
C	-2.96161000	-1.30322200	4.17273000
H	-3.74009000	-1.61954100	4.85820100
C	1.17524400	-0.61949900	-2.89567200
H	1.00284600	-0.77721000	-3.96369000
C	0.92558800	-0.32291900	5.57994600
H	0.90762700	-1.34805300	5.97248800
H	0.20181700	0.28407800	6.13880900
C	-2.20291800	-0.74243900	1.93614400
C	-1.66471300	-1.04063800	4.66468700
H	-1.47095200	-1.16209000	5.72472800
C	5.90294700	-0.07179300	0.95475100
H	6.40907500	0.75478600	0.43859000
H	6.43270500	-1.00767200	0.73398200
C	4.88558600	-0.67500800	-3.11908500
H	5.65538700	-0.77647900	-3.87610900
C	4.57408500	0.01078600	5.25080700
H	4.87322000	0.07425300	6.30819600
H	4.58936800	1.02551700	4.82854600
C	-2.15948600	0.06304800	-1.73977400
H	-2.06640300	1.14561700	-1.87433700
H	-3.19768500	-0.23165300	-1.91852200
C	5.55352900	-0.89587600	4.52788000
H	6.57660900	-0.58534100	4.78427800
H	5.41176000	-1.92532100	4.86803400
C	5.91861900	0.19995800	2.44051400
H	6.95925200	0.37698000	2.74840200
H	5.32875900	1.10014300	2.65690900
C	2.29423400	0.29209500	5.75050100
H	2.30997600	1.29123200	5.29309300
H	2.49595300	0.39719200	6.82691400
C	-0.03923100	-4.50842800	0.24047700
H	-1.03845700	-4.58404300	0.68211700
H	0.59827300	-5.27691200	0.67995300

H	-0.13925900	-4.67102000	-0.83506600
K	2.63922600	-0.88373000	2.42150300
O	0.06072200	1.62819600	-0.29971000
C	0.73255900	2.49137900	-0.95104600
O	1.59521800	2.33193500	-1.80414800
C	1.82384900	2.56932100	2.71782400
C	2.77933700	2.45456000	1.61643400
H	0.94765900	1.92434000	2.72204300
H	1.75061800	3.50784800	3.26560500
O	3.06349000	1.87236700	2.89909000
H	2.56149400	1.70396300	0.85840900
C	3.66323700	3.59014800	1.18789200
H	3.19004200	4.12902400	0.36126900
H	3.83549100	4.28845100	2.01243300
H	4.62884600	3.21009000	0.83882100
O	0.42473600	3.78757000	-0.63593600
C	-0.57458600	4.02551500	0.34862200
H	-0.29675200	3.58095700	1.30747700
H	-0.64032200	5.10983200	0.44678600
H	-1.54146700	3.62175600	0.03537500

TS1K.log

SCF (wB97xd) = -2884.60513446
E(SCF)+ZPE(0 K)= -2883.959750
H(298 K)= -2883.914698
G(298 K)= -2884.036085
Lowest Frequency = -536.5062cm-1

Co	0.01702300	-0.26157100	-0.04336400
O	0.07133500	-0.08876500	1.84509800
O	1.90976300	-0.32302100	-0.03353000
O	0.72829900	-0.38449800	4.35016200
O	-0.19912300	-2.13919800	-0.01208400
O	4.43928900	-0.18305900	0.51136500
O	5.41469200	-1.26465100	2.95756000
O	3.43506600	-0.87650400	5.17099800
O	1.20905000	-2.90245100	1.57448700
N	-0.09094400	-0.41180200	-1.94371700
N	-1.88680800	-0.18488600	-0.08814900
C	2.69524100	-0.20080200	-1.05667900
C	4.10553100	-0.11969200	-0.80425500
C	-0.88347800	-0.44316100	2.64589700
C	-0.57156900	-0.62095900	4.03654600
C	-2.66218100	-0.42407300	0.90271100
H	-3.73769900	-0.46428100	0.71349700
C	3.21760000	-0.02612100	-3.45496000
H	2.86251800	-0.00105900	-4.48150400
C	0.33784400	-3.05394100	0.72165700
C	-1.45431500	-0.68662900	-2.38984100
H	-1.61623900	-1.76679100	-2.32026900
H	-1.60740400	-0.36795800	-3.42501900
C	-3.21313200	-1.01640800	3.21293600
H	-4.23889900	-1.15606600	2.88261900
C	5.00599000	0.00879100	-1.84268300
H	6.07001900	0.06327300	-1.64244800
C	2.27249000	-0.16118900	-2.40808500
C	-2.87473700	-1.20563600	4.53108800
H	-3.62022700	-1.50318800	5.26016300
C	0.88597000	-0.32403000	-2.76788700
H	0.66274700	-0.39315400	-3.83568700
C	1.13813300	-0.44180400	5.70751800
H	1.05713100	-1.46975300	6.08443700
H	0.50554100	0.21286200	6.32068000
C	-2.23373000	-0.64079600	2.26107800
C	-1.54055100	-1.00235200	4.94295400
H	-1.28527500	-1.15168900	5.98616900
C	5.81233200	-0.15244600	0.87118500
H	6.29522800	0.73939300	0.45086100
H	6.31767400	-1.04754000	0.48562900
C	4.56070900	0.06360500	-3.18087700
H	5.28554000	0.16649600	-3.98074000
C	4.78519100	-0.44759100	5.20620400
H	5.16224900	-0.48158800	6.23992500
H	4.84665000	0.58824500	4.84580000
C	-2.39813200	0.03598600	-1.43548000
H	-2.38390700	1.11048800	-1.63236700
H	-3.42368200	-0.32953400	-1.53751600
C	5.64056100	-1.36954300	4.35325300
H	6.69836900	-1.16662100	4.57696700
H	5.42871100	-2.41030800	4.61324400
C	5.91824800	-0.07208000	2.37632200
H	6.97987800	0.05475900	2.63543800
H	5.34648800	0.79385500	2.73603800
C	2.56372000	0.05128400	5.79267600
H	2.65162700	1.02500600	5.29036600
H	2.82675600	0.16418300	6.85512700
C	-0.25231000	-4.42929600	0.45057400
H	-1.23969600	-4.48442100	0.92044800
H	0.38416000	-5.20681400	0.87478300
H	-0.38474300	-4.59222600	-0.62118600
K	2.66589800	-0.89760100	2.51314800
O	-0.01589600	1.73711200	-0.32745800
C	0.73995900	2.71004200	-0.55646800
O	1.75922700	3.08580000	0.07023700
C	2.22476400	2.27846400	1.71388900
C	3.57701300	2.68815600	2.10456300
H	2.10014000	1.28796600	1.31217400
H	1.40144800	2.70542200	2.27402700
O	3.40840400	1.83925400	3.18884100
H	4.34366100	2.34749900	1.38520100
C	3.79881600	4.15322100	2.44099000
H	3.81407300	4.76942100	1.53507400
H	3.00406500	4.51717800	3.10204100
H	4.75783700	4.27203200	2.95706700
O	0.43578100	3.52611900	-1.58634300
C	-0.69775400	3.20809400	-2.38898100
H	-1.61523300	3.25365900	-1.79682200
H	-0.72386000	3.96675500	-3.17020200
H	-0.59305800	2.21762100	-2.83694600

IIK.log

SCF (wB97xd) = -2884.62764833
E(SCF)+ZPE(0 K)= -2883.979874
H(298 K)= -2883.934925
G(298 K)= -2884.056033
Lowest Frequency = 24.1560cm-1

Co	-0.00794300	-0.22443900	-0.00791100
O	0.02772400	-0.09351300	1.88108800
O	1.88573100	-0.17984200	-0.01538300
O	0.69307700	-0.45589400	4.37546000
O	-0.16168800	-2.08945900	-0.01498700
O	4.43346900	-0.24029600	0.49552500
O	5.42641000	-1.20196100	2.96815800

O	3.42512700	-0.88869700	5.19578700
O	1.36699600	-2.77198000	1.49774700
N	-0.12979600	-0.32243600	-1.91191700
N	-1.91207600	-0.16004900	-0.04395500
C	2.66879000	-0.18429700	-1.04704900
C	4.08645800	-0.18989600	-0.81336400
C	-0.91234600	-0.51811800	2.66901700
C	-0.59286100	-0.73738800	4.05049900
C	-2.68633500	-0.46655500	0.93006400
H	-3.76118500	-0.50742900	0.73795600
C	3.18015700	-0.15793500	-3.45859400
H	2.81529200	-0.16081300	-4.48196000
C	0.46837000	-2.97496700	0.68939800
C	-1.50175400	-0.55013300	-2.36422500
H	-1.68822700	-1.62868900	-2.34424300
H	-1.65094800	-0.18087200	-3.38277900
C	-3.21827600	-1.20153900	3.20568000
H	-4.23816300	-1.36388800	2.86806300
C	4.98140900	-0.15510900	-1.86335600
H	6.04842300	-0.15876700	-1.67086400
C	2.23956000	-0.19194600	-2.39796600
C	-2.87109400	-1.43535300	4.51446900
H	-3.60397700	-1.79357600	5.22889200
C	0.84722200	-0.29242500	-2.74261800
H	0.61225400	-0.36402600	-3.80773700
C	1.11004200	-0.55114900	5.72941100
H	1.07012200	-1.59565900	6.06494900
H	0.45339100	0.05351300	6.36801700
C	-2.25310300	-0.74787500	2.27388900
C	-1.54678300	-1.19597000	4.93774500
H	-1.28610000	-1.37748600	5.97447900
C	5.80256100	-0.14430500	0.85709600
H	6.25164800	0.74739500	0.40051100
H	6.34379700	-1.03534100	0.51329500
C	4.52803400	-0.12962900	-3.19997900
H	5.24960100	-0.10227500	-4.00887600
C	4.74622400	-0.37007800	5.19492100
H	5.13505500	-0.34069500	6.22479600
H	4.72887300	0.65094600	4.78836800
C	-2.42305900	0.14870200	-1.37419800
H	-2.37514000	1.23245100	-1.51385900
H	-3.45994000	-0.17816300	-1.49054200
C	5.65034400	-1.26608800	4.36618100
H	6.69583000	-1.00450600	4.58830600
H	5.49170400	-2.31089200	4.64781400
C	5.88697100	-0.00425500	2.35928500
H	6.93936300	0.17397900	2.62685300
H	5.27505800	0.85048900	2.68809900
C	2.51558800	-0.00561000	5.82772400
H	2.56729100	0.97690000	5.33667800
H	2.77154200	0.10225900	6.89271900
C	-0.05560800	-4.37910800	0.43509200
H	-1.00564800	-4.49792200	0.96605200
H	0.65247300	-5.11946300	0.80905800
H	-0.24545000	-4.53979800	-0.62815400
K	2.65950100	-0.62867100	2.52122500
O	-0.04546800	1.83651700	-0.20841600
C	0.69198700	2.80522100	-0.37291400
O	1.59095000	3.26692100	0.43997500
C	1.76602000	2.69606500	1.77654300
C	3.25935900	2.56006900	2.10896400
H	1.26446600	1.73111000	1.81348200
H	1.27905300	3.39584700	2.46476700
O	3.37766900	1.91658300	3.30746000
H	3.69272900	1.97537800	1.26202000
C	3.94709100	3.93569700	2.07929400
H	3.84455800	4.43674400	1.10839200
H	3.51964800	4.58413800	2.85576100
H	5.01489600	3.81063400	2.29113800
O	0.63688500	3.57196500	-1.44639900
C	-0.33492000	3.24577100	-2.44784300
H	-1.34195700	3.34623300	-2.03861100
H	-0.17790300	3.97055400	-3.24350600
H	-0.17657900	2.23373900	-2.82009300

IIIK.log

SCF (wB97xd) =	-2884.60632550
E(SCF)+ZPE(0 K)=	-2883.960180
H(298 K)=	-2883.914229
G(298 K)=	-2884.042542

Lowest Frequency = 7.4794cm-1

Co	-0.42983300	-0.86665400	-0.33524200
O	-0.50706900	-0.24759500	1.44085100
O	1.32629800	-0.21908400	-0.48023100
O	0.18947300	0.04958800	3.96764200
O	-0.12627700	-2.64019000	0.06194500
O	3.83282400	0.40386000	-0.02179500
O	4.92865300	-0.18150500	2.54380100
O	2.94839800	0.13164900	4.74833600
O	1.56284400	-2.41393700	1.54186700
N	-0.52086300	-1.28986200	-2.19017000
N	-2.31262800	-1.16146000	-0.34808200
C	2.15394800	-0.36328100	-1.47469300
C	3.52961400	-0.01203500	-1.27459900
C	-1.33294300	-0.70360800	2.34208900
C	-0.99378100	-0.56508600	3.72771200
C	-3.03945200	-1.39266000	0.68450100
H	-4.08605100	-1.66289400	0.52758400
C	2.73923800	-0.91715200	-3.80449400
H	2.42059600	-1.27687200	-4.77850100
C	0.76021200	-3.08345200	0.91063600
C	-1.81002000	-1.87376900	-2.56738600
H	-1.76832500	-2.94646300	-2.35066900
H	-2.01401400	-1.73167500	-3.63159800
C	-3.43992600	-1.76177100	3.06958900
H	-4.39138400	-2.21057500	2.79941600
C	4.44166600	-0.10891100	-2.30829800
H	5.47893100	0.16135900	-2.14765900
C	1.78599900	-0.82825100	-2.76020200
C	-3.06918300	-1.64465600	4.38704000
H	-3.71425200	-2.00484700	5.18063500
C	0.44272900	-1.24495600	-3.04105800
H	0.23383700	-1.56936500	-4.06297400
C	0.62602900	0.20079200	5.31233000
H	0.74601200	-0.78498700	5.77960800
H	-0.10941200	0.78128800	5.88354000
C	-2.58121900	-1.30270200	2.04283600
C	-1.84004900	-1.03843300	4.71388500
H	-1.56558100	-0.94620900	5.75826900
C	5.19502000	0.63189000	0.31766300
H	5.59270300	1.47418600	-0.26260900
H	5.78718600	-0.26637100	0.10152500
C	4.04693700	-0.56179400	-3.58373500
H	4.78288900	-0.62970400	-4.37707000

C	4.20130500	0.79288200	4.69205500
H	4.58653500	0.95497900	5.71075800
H	4.07240100	1.76629700	4.19877400
C	-2.86607400	-1.21159100	-1.70051700
H	-3.04236700	-0.18519500	-2.03883900
H	-3.81226300	-1.75887800	-1.72427000
C	5.19158500	-0.07029800	3.93124300
H	6.20247300	0.33358600	4.09073400
H	5.16439300	-1.09052100	4.32426400
C	5.26623300	0.97122200	1.78752600
H	6.29470100	1.28766000	2.01657100
H	4.57282500	1.79633300	2.01186300
C	1.93671700	0.94938500	5.30527300
H	1.84129100	1.86705700	4.70717000
H	2.18806300	1.21453300	6.34318800
C	0.67359100	-4.59068100	1.06941200
H	1.58167700	-4.96684400	1.54132300
H	0.51571100	-5.07907800	0.10602200
H	-0.18289100	-4.82461400	1.70946400
K	2.09208000	0.28691000	2.05737600
O	6.27397000	6.08406000	2.46495300
C	5.42741600	6.42155700	3.26205300
O	4.29435300	5.79600500	3.51134900
C	4.03224700	4.58045500	2.76589300
C	2.70988400	3.95068900	3.22720600
H	4.85829200	3.88473900	2.93938700
H	3.98647500	4.82431900	1.69823400
H	2.81185800	3.83226000	4.33826500
C	1.54168700	4.92750400	3.00807900
H	1.67990000	5.87705500	3.53940700
H	1.43053400	5.13757600	1.93607600
H	0.61408700	4.46352900	3.36100800
O	5.49663700	7.50142800	4.03829000
C	6.67373900	8.30324300	3.89901800
H	6.75680100	8.68699700	2.87999700
H	6.54871900	9.12403900	4.60271700
H	7.56395800	7.72212300	4.14891400
O	2.54462900	2.76038000	2.58914400

IVK.log

SCF (wB97xd) = -3073.18985366
E(SCF)+ZPE(0 K)= -3072.530660
H(298 K)= -3072.480760
G(298 K)= -3072.618289
Lowest Frequency = 9.0014cm-1

Co	-0.24180700	-0.56758600	-0.11224300
O	-0.23012800	-0.11771600	1.71613800
O	1.57978400	-0.14082100	-0.21863300
O	0.51939400	-0.20555500	4.24888500
O	-0.16974900	-2.39413900	0.10562200
O	4.14538600	0.19396900	0.23444700
O	5.12113700	-1.00137300	2.60836400
O	3.25239700	-0.73366600	4.90761900
O	1.54402400	-2.51557700	1.57036100
N	-0.39519100	-0.76410900	-1.99804300
N	-2.14623000	-0.60354600	-0.12515000
C	2.36403900	-0.18742600	-1.25384600
C	3.76981000	0.01342200	-1.05605300
C	-1.10634900	-0.54659300	2.58243800
C	-0.74384400	-0.60856700	3.96724000
C	-2.89628500	-0.82557100	0.89299300
H	-3.97034300	-0.93417400	0.72721100
C	2.84097600	-0.40610300	-3.66371100
H	2.46760000	-0.57976400	-4.66874000
C	0.66025000	-3.01984300	0.89606300
C	-1.74189600	-1.16903500	-2.40391700
H	-1.81894900	-2.25390900	-2.27548700
H	-1.93521700	-0.91757500	-3.44977800
C	-3.34086700	-1.36474200	3.23626400
H	-4.34774800	-1.64317000	2.93896000
C	4.64278100	0.01437100	-2.12670500
H	5.70268400	0.17259000	-1.96408600
C	1.92617000	-0.41105700	-2.58198100
C	-2.95228700	-1.43974800	4.55164000
H	-3.63963400	-1.78425600	5.31618400
C	0.54775600	-0.67844100	-2.86920800
H	0.28653200	-0.83918200	-3.91750500
C	0.99482900	-0.33503200	5.58260000
H	0.93635400	-1.38430000	5.89879900
H	0.38867600	0.27848000	6.26093600
C	-2.42837600	-0.92911900	2.24676400
C	-1.64660200	-1.05591500	4.91512500
H	-1.35830600	-1.11559200	5.95805200
C	5.53133400	0.13774600	0.55627600
H	6.05432200	0.99947100	0.12389300
H	5.96310600	-0.78866800	0.15721300
C	4.17896800	-0.19315200	-3.44191300
H	4.88546100	-0.18881500	-4.26433500
C	4.61044900	-0.32768300	4.92526300
H	5.02209600	-0.43567200	5.94067100
H	4.67275300	0.72648000	4.62419800
C	-2.70887600	-0.46609100	-1.46824400
H	-2.76213900	0.59976400	-1.71342000
H	-3.71369900	-0.89303600	-1.52499500
C	5.41132500	-1.20304400	3.97891700
H	6.48182400	-1.03144400	4.16682500
H	5.19251800	-2.25548600	4.18088100
C	5.66705200	0.18897000	2.05840800
H	6.73723400	0.26486500	2.30305600
H	5.14063500	1.07458400	2.44743700
C	2.42160200	0.15342900	5.63090200
H	2.49244700	1.15966200	5.19344900
H	2.73416100	0.19787800	6.68492700
C	0.38485700	-4.51264500	0.92128200
H	1.24843700	-5.04238300	1.32435100
H	0.14103400	-4.88570300	-0.07511400
H	-0.47775000	-4.69549000	1.56979100
K	2.41301100	0.09926700	2.32768800
O	7.91918000	4.28983400	4.27480100
C	7.06933500	4.72199800	5.02110200
O	5.78235900	4.43995700	4.98763400
C	5.33577100	3.51771700	3.96208500
C	3.82459000	3.27728800	4.10281900
H	5.88060400	2.57711700	4.08586900
H	5.57034700	3.93843700	2.97985800
H	3.67497300	2.97092200	5.17026800
C	3.05433000	4.59810000	3.92704600
H	3.35376500	5.35925800	4.65740800
H	3.22339400	4.99922900	2.91905500
H	1.98144400	4.40932900	4.04137600
O	7.29306200	5.57050600	6.02298600
C	8.65084800	5.98317200	6.20743300
H	9.01659000	6.50325900	5.31952600
H	8.63382600	6.65878600	7.06052500

H 9.28713500 5.12125400 6.41926100
O 3.43923700 2.30754700 3.22607500
C 3.47319100 3.06189600 0.73923200
O 2.33742200 2.86261700 0.60392500
O 4.60578200 3.32067800 0.69895100

TS2K.log

SCF (wB97xd) = -3073.18976408
E(SCF)+ZPE(0 K)= -3072.530789
H(298 K)= -3072.481691
G(298 K)= -3072.616858
Lowest Frequency = -108.9061cm-1

Co -0.24646800 -0.57804300 -0.11869100
O -0.23141200 -0.12277000 1.70828600
O 1.57519500 -0.15340600 -0.22922800
O 0.52227100 -0.19409300 4.23984200
O -0.17117100 -2.40402400 0.10300900
O 4.14211200 0.17865100 0.21769400
O 5.13056400 -0.98626700 2.59678600
O 3.25894200 -0.69688700 4.88939100
O 1.53936400 -2.51937400 1.57209200
N -0.40409200 -0.77795400 -2.00363800
N -2.15084100 -0.61675700 -0.12689100
C 2.35671100 -0.19876600 -1.26665900
C 3.76295400 0.00241400 -1.07196900
C -1.10500400 -0.54982700 2.57799100
C -0.73994400 -0.60416500 3.96231400
C -2.89791600 -0.83860100 0.89345700
H -3.97211000 -0.95011900 0.73053200
C 2.82697900 -0.41468800 -3.67812500
H 2.45094000 -0.58759600 -4.68228200
C 0.65847700 -3.02665800 0.89611200
C -1.75109300 -1.18553000 -2.40575200
H -1.82619200 -2.27033900 -2.27558800
H -1.94726300 -0.93590200 -3.45153100
C -3.33539600 -1.37258300 3.23946600
H -4.34198200 -1.65540500 2.94534100
C 4.63267700 0.00507600 -2.14535300
H 5.69306700 0.16306400 -1.98608200
C 1.91527600 -0.42169900 -2.59367900
C -2.94380200 -1.44156600 4.55430800
H -3.62831200 -1.78583300 5.32148900
C 0.53655400 -0.69096100 -2.87722000
H 0.27287400 -0.85240800 -3.92479200
C 1.00097400 -0.31626800 5.57279700
H 0.95138600 -1.36491200 5.89237700
H 0.39235000 0.29495400 6.25092200
C -2.42654300 -0.93749500 2.24645400
C -1.63889100 -1.05134000 4.91377800
H -1.34840000 -1.10592600 5.95638000
C 5.52990900 0.13509800 0.53147100
H 6.04374200 0.99859400 0.09157200
H 5.96658400 -0.79013800 0.13478300
C 4.16524600 -0.20074700 -3.45962700
H 4.86955100 -0.19472500 -4.28392600
C 4.61525500 -0.28475000 4.90415800
H 5.02571200 -0.37807700 5.92132500
H 4.67491200 0.76593100 4.59076700
C -2.71698200 -0.48266800 -1.46887200
H -2.77256000 0.58269100 -1.71567800
H -3.72125600 -0.91131200 -1.52244400
C 5.42087000 -1.16891000 3.96990000
H 6.49035200 -0.99014300 4.15654100
H 5.20623100 -2.21935400 4.18600600
C 5.67580500 0.19740100 2.03207000
H 6.74788800 0.27264900 2.26847700
H 5.15452100 1.08763800 2.41600600
C 2.42425300 0.18294600 5.61595100
H 2.48329400 1.19098600 5.18128800
H 2.74086500 0.23118000 6.66846000
C 0.38704200 -4.52014100 0.92219600
H 1.24958200 -5.04685000 1.33139100
H 0.15056800 -4.89533000 -0.07518600
H -0.47918700 -4.70446600 1.56541300
K 2.41184200 0.09156400 2.31182900
O 7.94001400 4.30971900 4.29197500
C 7.08120400 4.71566900 5.04245100
O 5.79476700 4.43294300 4.98412300
C 5.36313500 3.54616700 3.92300600
C 3.85097500 3.30230500 4.04428600
H 5.90693500 2.60195800 4.02105000
H 5.60888800 3.99978000 2.95859300
H 3.69240600 2.96692200 5.09907500
C 3.07904400 4.62464400 3.89543700
H 3.37044100 5.36424800 4.65032300
H 3.25723200 5.05724700 2.90228100
H 2.00566900 4.43101500 3.99435600
O 7.29094600 5.52997600 6.07479200
C 8.64537800 5.93974400 6.28876100
H 9.01940700 6.49067900 5.42322900
H 8.61649800 6.58599500 7.16397700
H 9.28176600 5.07280500 6.47855400
O 3.46783700 2.34796300 3.14412500
C 3.43407000 3.05668900 0.87127700
O 2.29746600 2.85668100 0.70888000
O 4.55896800 3.34222800 0.74949700

VK.log

SCF (wB97xd) = -3073.22363703
E(SCF)+ZPE(0 K)= -3072.561140
H(298 K)= -3072.513200
G(298 K)= -3072.645914
Lowest Frequency = -8.2277cm-1

Co -0.30239700 -0.79679100 -0.30620300
O -0.35319500 -0.24596500 1.49229700
O 1.50381100 -0.30043500 -0.39703600
O 0.32788500 -0.08387500 4.04667200
O -0.14907300 -2.60615600 0.00728700
O 4.03879100 0.14702800 0.10955100
O 5.01393200 -0.64418800 2.61442500
O 3.06857700 -0.30039200 4.77630300
O 1.50577300 -2.58489100 1.54115500
N -0.39570900 -1.11730900 -2.18101700
N -2.19792600 -0.94529900 -0.35104600
C 2.32583800 -0.41250900 -1.39938300
C 3.71782400 -0.15398800 -1.17294500
C -1.22582400 -0.65957000 2.36933000
C -0.89820800 -0.59471400 3.76296300
C -2.95493700 -1.15362600 0.66463300
H -4.01675200 -1.33620400 0.48576500
C 2.88920300 -0.81906200 -3.76392500
H 2.55539700 -1.08521400 -4.76261700

C	0.67371200	-3.15859400	0.85498700
C	-1.71494400	-1.60125100	-2.59321100
H	-1.74469800	-2.68181400	-2.41756900
H	-1.89698000	-1.40555800	-3.65285600
C	-3.42186400	-1.56918800	3.02953400
H	-4.40179400	-1.93037600	2.73118300
C	4.62735000	-0.22135500	-2.21050800
H	5.67711000	-0.02041500	-2.03001500
C	1.93778600	-0.75708800	-2.71613900
C	-3.06335300	-1.52878100	4.35499600
H	-3.74700800	-1.86397500	5.12689300
C	0.57437000	-1.07759600	-3.02503300
H	0.35282600	-1.32693200	-4.06514900
C	0.77013200	-0.09262300	5.39837100
H	0.79245700	-1.12334200	5.77418900
H	0.09260300	0.50258000	6.02305400
C	-2.51313900	-1.14515000	2.03195700
C	-1.79555700	-1.03462900	4.71923400
H	-1.53391800	-1.00221600	5.77028700
C	5.40641200	0.29052100	0.46151200
H	5.84576600	1.15392400	-0.05364700
H	5.96055900	-0.61555700	0.18661200
C	4.21336700	-0.55324300	-3.51686900
H	4.94763300	-0.60003000	-4.31322500
C	4.40160500	0.17006800	4.85722900
H	4.75867400	0.12788000	5.89713900
H	4.44613600	1.21441800	4.52228400
C	-2.73573100	-0.90507400	-1.71063000
H	-2.83860300	0.14353200	-2.00858500
H	-3.71592100	-1.38597100	-1.76601400
C	5.29354500	-0.71055800	4.00198100
H	6.34192600	-0.43946100	4.19193800
H	5.15062900	-1.75707100	4.28334800
C	5.48811300	0.51288400	1.95459400
H	6.53762400	0.70731300	2.21783600
H	4.89670600	1.39809200	2.23151600
C	2.14720200	0.52319900	5.46694300
H	2.14156300	1.52981400	5.02636300
H	2.42846900	0.60552500	6.52711400
C	0.46668700	-4.65980800	0.93687700
H	1.34036300	-5.13074900	1.38816900
H	0.27139800	-5.08548600	-0.04895000
H	-0.40642900	-4.85505800	1.56745600
K	2.24406100	0.00483700	2.13599100
O	8.11068600	4.48733500	3.86557400
C	7.54300900	4.57082300	4.92926100
O	6.24502600	4.40294300	5.13256100
C	5.44494300	4.11983500	3.96871500
C	4.01587700	3.93486100	4.48077400
H	5.80715600	3.19992100	3.50079200
H	5.52943600	4.94196500	3.25843600
H	4.09236000	3.34115700	5.39947200
C	3.31505100	5.24615900	4.80976400
H	3.89486100	5.79498700	5.55800000
H	3.21401700	5.86473100	3.91545300
H	2.32275600	5.04640900	5.22363800
O	8.11845200	4.84252800	6.09394200
C	9.53602800	5.04798000	6.06332700
H	9.78379900	5.89656100	5.42262300
H	9.81864500	5.25570400	7.09333500
H	10.04282300	4.15021200	5.70366900
O	3.25074400	3.09587000	3.62053800
C	2.98566100	3.47250200	2.29112100
O	2.19094600	2.69203400	1.73886600
O	3.56438900	4.47314600	1.84500000

VIIK.log

SCF (wB97xd) = -3266.36468376
E(SCF)+ZPE(0 K)= -3265.612499
H(298 K)= -3265.558632
G(298 K)= -3265.702792
Lowest Frequency = 9.7636cm-1

Co	0.25470900	-0.27274000	-0.38007300
O	0.13967800	-0.01873800	1.50855400
O	2.13950100	-0.28035600	-0.16718600
O	0.50645000	0.02781300	4.08626000
O	0.05713900	-2.17907800	-0.28252400
O	4.60883200	-0.19646600	0.62799000
O	5.24661100	-1.13615200	3.21006300
O	3.08386000	-0.56233100	5.15617700
O	1.28998100	-2.91421900	1.45287100
N	0.33755100	-0.53888900	-2.26351600
N	-1.63753100	-0.21972500	-0.61971500
C	3.02834300	-0.38165500	-1.09955700
C	4.40958500	-0.33965800	-0.71163200
C	-0.92128400	-0.17538200	2.22737600
C	-0.77231500	-0.16979200	3.65732600
C	-2.51782600	-0.28834300	0.30703500
H	-3.57022700	-0.27781800	0.01597800
C	3.80390400	-0.64582100	-3.42059400
H	3.55598800	-0.76891900	-4.47159000
C	0.50388200	-3.07768700	0.51726700
C	-0.97658600	-0.81160000	-2.84401400
H	-1.16259600	-1.88731600	-2.76228700
H	-1.01291700	-0.52186200	-3.89802200
C	-3.33726400	-0.49953300	2.59865300
H	-4.33161300	-0.61143300	2.17466400
C	5.41870200	-0.43948200	-1.64866400
H	6.45820500	-0.40723700	-1.34213800
C	2.75203100	-0.54418800	-2.47944900
C	-3.15537600	-0.51992200	3.96014100
H	-3.99475000	-0.65609300	4.63302900
C	1.40208400	-0.62993900	-2.97046400
H	1.28528200	-0.81362000	-4.04165900
C	0.76372600	-0.02617700	5.47984600
H	0.56660800	-1.03821800	5.85715900
H	0.12045300	0.68320000	6.01608800
C	-2.23811500	-0.33637700	1.72076900
C	-1.85776800	-0.35368400	4.48986700
H	-1.72846800	-0.37147000	5.56599100
C	5.93841200	-0.22056400	1.11765000
H	6.52356000	0.59756900	0.67694200
H	6.41472700	-1.17656000	0.86338900
C	5.11738800	-0.59158500	-3.01880300
H	5.92439100	-0.66957400	-3.73896000
C	4.43954800	-0.20356900	5.34768200
H	4.68960100	-0.22955100	6.41920100
H	4.60618600	0.82034200	4.98389700
C	-2.00356700	-0.05006600	-2.02040200
H	-1.95049700	1.01809400	-2.25689700
H	-3.02069500	-0.40176500	-2.21445500
C	5.33673000	-1.19038600	4.62208200
H	6.37483800	-1.01869700	4.94168500
H	5.05565200	-2.20937300	4.90162800

C 5.90242000 -0.02937400 2.61526900
H 6.93837500 0.03742700 2.97797400
H 5.38051900 0.90636400 2.85106400
C 2.19778500 0.37227000 5.73436800
H 2.38583700 1.37103400 5.31917600
H 2.34809000 0.41789100 6.82341800
C -0.06373200 -4.46068400 0.22875400
H -1.08776200 -4.50607700 0.61403300
H 0.53127800 -5.23046400 0.72222500
H -0.10561000 -4.64721900 -0.84668700
K 2.56443700 -0.83027300 2.42597700
O 0.15122100 1.64987000 -0.51905200
C 0.72135900 2.53318500 -1.23259700
O 1.62909200 2.42115800 -2.04182000
C 2.29704400 2.89551000 3.06444900
C 2.70259500 2.41699000 1.74384600
H 1.34181300 2.57511600 3.47676600
H 2.68856800 3.84100300 3.43662500
O 3.26786900 1.85458700 2.93932600
H 2.01998400 1.73850000 1.23392500
C 3.64105200 3.18364700 0.85785200
H 3.06827700 3.79585600 0.15429300
H 4.29368800 3.83519100 1.44673700
H 4.25677100 2.48873000 0.27876100
O 0.22398000 3.80601100 -1.05832600
C -0.82951200 4.09186800 -0.12603700
H -1.00848800 5.15865400 -0.29171900
C -2.10644800 3.34624900 -0.50929500
H -2.04733800 2.29825900 -0.22560800
H -2.29559100 3.43387000 -1.58383800
C -0.42862200 3.87473200 1.32511200
H 0.49270000 4.42201500 1.54209800
H -1.21683000 4.25829000 1.97922600
H -0.27267400 2.81387100 1.53105500
O -3.18155700 3.97341500 0.20876700
C -4.30501600 3.27570700 0.30399500
O -4.51532200 2.19799700 -0.20179100
O -5.16186500 3.95949300 1.04931400
C -6.43768900 3.34358800 1.26571100
H -6.31515400 2.38876900 1.78104000
H -6.99208800 4.04246000 1.88861700
H -6.95223700 3.19015700 0.31515600

Functional Screening

1_prop_Z_b3lyp.log

SCF (B3LYP) = -2692.37070339
E(SCF)+ZPE(0 K)= -2691.819971
H(298 K)= -2691.779130
G(298 K)= -2691.893649
Lowest Frequency = 18.4880cm-1

Co 2.465382 6.369457 23.124233
K 5.793452 7.544303 22.007584
O 1.555813 6.146078 21.433872
O 3.149596 8.070537 22.613047
O 3.994588 5.585525 22.276993
O 4.618429 10.109003 21.945662
O 3.279972 6.416462 24.875614
O 5.924161 5.081197 20.606731
O 8.082598 6.885564 20.498484
O 7.164346 9.663763 20.781051
O 5.373928 7.228923 24.590069
O 0.436771 8.109044 21.176343
N 1.747427 4.669798 23.639503
N 0.929141 7.107088 23.971983
C 4.096947 4.349161 21.901042
C 5.171140 4.007456 21.007479
C 2.827260 9.195232 23.172859
C 3.623337 10.350846 22.858760
C 0.861046 7.019429 20.803982
C 0.803505 8.326536 24.351045
H -0.088550 8.596436 24.922118
C 3.484723 1.955713 21.895781
H 2.821892 1.168841 22.245025
C 4.434238 6.816991 25.284213
C 0.652818 4.811024 24.598831
H 1.087807 4.911633 25.598813
H -0.007647 3.938716 24.580434
C 1.487670 10.661752 24.641061
H 0.643269 10.774143 25.315685
C 5.387968 2.699031 20.608459
H 6.211000 2.456309 19.946562
C 3.243489 3.287487 22.323711
C 2.301596 11.738555 24.354353
H 2.121939 12.708368 24.805732
C 2.138219 3.521020 23.216195
H 1.595346 2.636893 23.560980
C 5.469155 11.185280 21.555211
H 6.103635 11.485651 22.399062
H 4.872507 12.048413 21.235073
C 1.731611 9.388479 24.064541
C 3.376984 11.578954 23.450171
H 4.006165 12.432151 23.225015
C 7.052356 4.849545 19.764515
H 6.751626 4.302926 18.861913
H 7.805421 4.261457 20.304232
C 4.538473 1.660581 21.055810
H 4.723895 0.641985 20.732429
C 8.038556 9.241225 19.739509
H 8.729578 10.054455 19.471301
H 7.456502 8.988273 18.841626
C -0.089466 6.095013 24.233325
H -0.664371 5.943274 23.312706
H -0.770638 6.411175 25.029335
C 8.854823 8.052554 20.221051
H 9.625701 7.825726 19.470923
H 9.349968 8.307990 21.161816
C 7.615746 6.189891 19.346825
H 8.444117 6.016257 18.645262
H 6.839925 6.768138 18.826902
C 6.309432 10.729102 20.383603
H 5.658092 10.400076 19.560762
H 6.904878 11.584079 20.031534
C 4.569637 6.775579 26.804835
H 4.044108 7.639764 27.227153
H 5.620011 6.827432 27.097238

H	4.107382	5.874259	27.215115
O	0.590313	6.560666	19.529918
C	-0.208392	7.419213	18.708263
H	0.284116	8.383494	18.552904
H	-0.316451	6.897746	17.756122
H	-1.192231	7.588104	19.155727

RPO_b3lyp.log

SCF (B3LYP) = -193.159176625
E(SCF)+ZPE(0 K)= -193.073804
H(298 K)= -193.068440
G(298 K)= -193.100182
Lowest Frequency = 212.7886cm-1

C	-0.642510	-0.271618	0.021008
C	0.825112	-0.266786	-0.002851
O	0.087759	0.972054	-0.029333
H	-1.155111	-0.500866	-0.914273
H	1.357649	-0.504628	-0.922102
H	1.371620	-0.453031	0.920703
C	-1.425589	-0.545272	1.278122
H	-2.347867	0.045319	1.295208
H	-1.700902	-1.604726	1.325038
H	-0.835061	-0.298567	2.165586

TS1Co _b3lyp.log

SCF (B3LYP) = -2885.52525199
E(SCF)+ZPE(0 K)= -2884.887622
H(298 K)= -2884.841857
G(298 K)= -2884.965592
Lowest Frequency = -459.2518cm-1

Co	5.119594	7.657517	21.276762
O	4.562469	5.951873	21.945703
O	3.628291	8.444583	22.174486
O	3.631280	4.020234	23.440474
O	6.340554	7.951076	22.737163
O	1.337742	8.939825	23.300689
O	0.487249	7.316559	25.462092
O	1.449855	4.491727	25.239308
O	5.178837	7.333148	24.580887
N	5.713916	9.328222	20.563575
N	6.587635	6.895668	20.325072
C	3.159573	9.639397	21.985038
C	1.903665	9.972128	22.604682
C	5.349092	4.927946	22.110022
C	4.889386	3.843889	22.935183
C	7.129028	5.755518	20.567015
H	8.038408	5.492494	20.020894
C	3.209720	11.949035	21.108646
H	3.726456	12.707110	20.526628
C	6.194142	7.738933	24.002871
C	7.039132	9.217563	19.953846
H	7.788055	9.314644	20.747335
H	7.201228	10.003740	19.210265
C	7.411980	3.618798	21.734382
H	8.379709	3.536112	21.247676
C	1.353745	11.237260	22.480256
H	0.413492	11.474563	22.964238
C	3.798943	10.661939	21.225090
C	6.960432	2.619884	22.571743
H	7.566828	1.742309	22.768385
C	5.068851	10.440198	20.586372
H	5.520983	11.302691	20.089762
C	3.051394	2.991223	24.245133
H	3.582313	2.925124	25.203567
H	3.123150	2.024489	23.731633
C	6.625406	4.775496	21.493984
C	5.686313	2.734714	23.172539
H	5.337335	1.940906	23.822496
C	0.086377	9.145020	23.957502
H	-0.653055	9.542729	23.251004
H	0.212615	9.858357	24.782004
C	2.009306	12.236551	21.723644
H	1.561794	13.220816	21.637111
C	0.084336	4.868289	25.410153
H	-0.447040	4.095678	25.987216
H	-0.393045	4.958047	24.426216
C	7.128025	7.824242	19.334630
H	6.496759	7.775227	18.441231
H	8.155808	7.564250	19.063748
C	0.010617	6.178251	26.179685
H	-1.030400	6.343971	26.492652
H	0.634600	6.110991	27.075508
C	-0.409977	7.808048	24.465651
H	-1.409563	7.954352	24.900139
H	-0.481763	7.101276	23.630123
C	1.587627	3.312308	24.451246
H	1.095790	3.457791	23.481573
H	1.117925	2.459680	24.964852
C	7.470137	8.013161	24.796097
H	7.239765	8.147469	25.854690
H	7.994276	8.890315	24.408673
H	8.139477	7.151587	24.690430
K	2.687074	6.579385	23.875051
O	0.666706	5.630575	22.147946
C	0.498415	5.000841	21.091743
O	0.900725	5.289367	19.925214
C	2.384869	6.767938	19.931683
C	3.548538	6.212192	19.251916
H	2.340792	6.749541	21.005012
H	1.744281	7.449318	19.389948
O	4.041250	7.493341	19.668171
H	4.025755	5.382917	19.772929
C	3.532748	6.036910	17.751295
H	2.980896	5.128513	17.487870
H	3.055296	6.892897	17.264285
H	4.555887	5.942215	17.373728
O	-0.223743	3.827568	21.224307
C	-0.414594	3.028345	20.050597
H	0.544520	2.705506	19.635338
H	-0.990454	2.160021	20.375387
H	-0.969164	3.578035	19.284716

TS1K_b3lyp.log

SCF (B3LYP) = -2885.50316208
E(SCF)+ZPE(0 K)= -2884.865514
H(298 K)= -2884.819735
G(298 K)= -2884.943403
Lowest Frequency = -435.0757cm-1

Co	0.00742400	-0.25091800	-0.05020800
O	0.05668000	-0.09285600	1.84950700

O	1.91072900	-0.27622400	-0.03203700
O	0.70643200	-0.38601700	4.36799400
O	-0.18110500	-2.13719400	-0.02520200
O	4.45857100	-0.19342100	0.50976600
O	5.42651300	-1.27188200	2.98021800
O	3.42878300	-0.88421800	5.19990900
O	1.29584600	-2.87450500	1.52828400
N	-0.09266800	-0.39317500	-1.95417700
N	-1.90240300	-0.19595300	-0.09789300
C	2.70151800	-0.18063400	-1.06023100
C	4.11726000	-0.12798400	-0.81238200
C	-0.90749100	-0.43818000	2.65150600
C	-0.60236600	-0.61289800	4.04688200
C	-2.68204400	-0.43304100	0.89653400
H	-3.75625400	-0.48353200	0.70212400
C	3.22480100	-0.03205400	-3.46981500
H	2.86651300	-0.00907100	-4.49503700
C	0.40583300	-3.04285200	0.69238500
C	-1.45367300	-0.69117700	-2.40412900
H	-1.59845100	-1.77435700	-2.33475400
H	-1.60646100	-0.37697700	-3.44108800
C	-3.25053800	-0.99850700	3.21094200
H	-4.27440500	-1.13601200	2.87473500
C	5.02150200	-0.02185800	-1.85678900
H	6.08567500	0.01205600	-1.65587800
C	2.27535300	-0.14623300	-2.41943700
C	-2.91754500	-1.18094600	4.53666300
H	-3.66820700	-1.46976100	5.26436600
C	0.89081500	-0.30147900	-2.77791700
H	0.66956800	-0.36882200	-3.84617300
C	1.11652400	-0.46582900	5.73537000
H	1.03597600	-1.50120400	6.09059700
H	0.47718400	0.17404300	6.35619300
C	-2.26362500	-0.63351800	2.25769300
C	-1.58155500	-0.98441900	4.95454300
H	-1.32935500	-1.13102000	5.99825700
C	5.84287600	-0.19219100	0.86951600
H	6.34600600	0.67759500	0.42931300
H	6.32060400	-1.10755400	0.49778300
C	4.57434600	0.03581700	-3.19692000
H	5.30001300	0.12098600	-3.99848100
C	4.78260300	-0.43074200	5.22757200
H	5.15789200	-0.44353800	6.26249500
H	4.82533700	0.59881300	4.85007400
C	-2.41442400	0.01557800	-1.45075300
H	-2.42015400	1.08976300	-1.65049700
H	-3.43371500	-0.36878800	-1.55174000
C	5.65065300	-1.35717700	4.38828500
H	6.70639900	-1.14247200	4.60927200
H	5.44593100	-2.39725100	4.65788600
C	5.95375500	-0.08895400	2.37565500
H	7.01785600	0.01895000	2.63232300
H	5.40214900	0.79101700	2.72847500
C	2.54119900	0.03445300	5.83471900
H	2.62708700	1.01554800	5.34951900
H	2.79983400	0.13166300	6.89979600
C	-0.15581800	-4.43628000	0.42598600
H	-1.08116700	-4.55419200	1.00122300
H	0.55617800	-5.19733600	0.75026000
H	-0.39973900	-4.56953800	-0.63040200
K	2.69990700	-0.86146300	2.54164700
O	-0.06098500	1.76092400	-0.32340500
C	0.69166200	2.74293000	-0.54209500
O	1.72025800	3.12062800	0.08028200
C	2.17255500	2.32080900	1.75402000
C	3.56363300	2.65567200	2.08855100
H	1.96381900	1.33098000	1.39382400
H	1.39780700	2.81806400	2.32480800
O	3.41634800	1.84017000	3.21235700
H	4.27696100	2.25995100	1.34481300
C	3.87762700	4.11848600	2.37465400
H	3.87510700	4.71193500	1.45252400
H	3.13864900	4.53928400	3.06666400
H	4.86805500	4.20138700	2.83696500
O	0.37086600	3.58137100	-1.56352400
C	-0.76670500	3.25256000	-2.37542400
H	-1.67964800	3.24881700	-1.77509700
H	-0.81903000	4.03724100	-3.12990100
H	-0.63207400	2.28120500	-2.85417800

1_prop_Z_m06.log

SCF (M06) =	-2690.95248941
E(SCF)+ZPE(0 K)=	-2690.403122
H(298 K)=	-2690.362769
G(298 K)=	-2690.474913

Lowest Frequency = 22.8394cm-1

Co	2.479317	6.354948	23.114870
K	5.794711	7.514012	22.040774
O	1.610203	6.108028	21.416865
O	3.153286	8.048489	22.579303
O	4.026251	5.573602	22.311015
O	4.657836	10.068239	21.963905
O	3.274890	6.430023	24.864461
O	5.924007	5.092149	20.625857
O	8.068677	6.870477	20.479383
O	7.166561	9.611295	20.795276
O	5.289726	7.411990	24.607103
O	0.408130	8.011149	21.173128
N	1.764305	4.665898	23.635878
N	0.941600	7.089282	23.941143
C	4.112622	4.353323	21.911156
C	5.173707	4.021224	21.005285
C	2.864374	9.155832	23.169212
C	3.679697	10.300933	22.884147
C	0.879371	6.949989	20.797691
C	0.836765	8.302013	24.336541
H	-0.054454	8.578803	24.914242
C	3.483680	1.972079	21.860824
H	2.813403	1.183422	22.200321
C	4.368347	6.954437	25.284607
C	0.664984	4.811047	24.578930
H	1.088105	4.920254	25.586398
H	0.010641	3.930846	24.563663
C	1.568013	10.607867	24.682603
H	0.727045	10.716632	25.366487
C	5.375662	2.724662	20.577566
H	6.193519	2.490612	19.900611
C	3.256182	3.293140	22.313780
C	2.400866	11.671076	24.424204
H	2.245671	12.632905	24.905036
C	2.155061	3.522897	23.206158
H	1.606933	2.633774	23.544202
C	5.512104	11.134267	21.598097
H	6.153318	11.414615	22.448866

H	4.925941	12.016281	21.298321
C	1.781649	9.350661	24.070974
C	3.463595	11.512098	23.511148
H	4.112775	12.359460	23.305796
C	6.990319	4.892316	19.718614
H	6.635932	4.380085	18.810987
H	7.776189	4.278056	20.185400
C	4.525181	1.685552	21.009901
H	4.699886	0.670637	20.663726
C	8.040272	9.218714	19.758222
H	8.737150	10.037931	19.511823
H	7.464041	8.996981	18.842618
C	-0.073090	6.081598	24.195056
H	-0.639378	5.920852	23.266541
H	-0.769535	6.407124	24.977580
C	8.843872	8.021972	20.208649
H	9.605663	7.797748	19.443642
H	9.360854	8.262325	21.144779
C	7.527850	6.243564	19.335702
H	8.303369	6.114760	18.562547
H	6.712734	6.847655	18.900901
C	6.342368	10.694856	20.425033
H	5.685770	10.401171	19.587941
H	6.955744	11.548241	20.090495
C	4.438802	7.008610	26.798506
H	3.817219	7.845214	27.141719
H	5.465247	7.175862	27.133961
H	4.036160	6.096410	27.248007
O	0.629729	6.489632	19.529793
C	-0.196724	7.310391	18.721074
H	0.253363	8.297135	18.564707
H	-0.292266	6.794332	17.763335
H	-1.188012	7.442768	19.168925

RPO_m06.log

SCF (M06) = -193.014664366
E(SCF)+ZPE(0 K)= -192.929473
H(298 K)= -192.924126
G(298 K)= -192.955836
Lowest Frequency = 203.5281cm-1

C	-0.644677	-0.270011	0.017470
C	0.814364	-0.262907	0.001549
O	0.081010	0.953102	-0.022588
H	-1.153706	-0.500027	-0.922493
H	1.355159	-0.498174	-0.915625
H	1.353230	-0.458011	0.930980
C	-1.418064	-0.543481	1.270050
H	-2.340506	0.047248	1.298672
H	-1.694238	-1.602348	1.326828
H	-0.817471	-0.293512	2.152264

TS1Co_m06.log

SCF (M06) = -2883.96132962
E(SCF)+ZPE(0 K)= -2883.325530
H(298 K)= -2883.280522
G(298 K)= -2883.401830
Lowest Frequency = -479.4881cm-1

Co	5.172040	7.655785	21.325300
O	4.650047	5.962612	22.030275
O	3.692649	8.437347	22.237572
O	3.786932	4.069927	23.591123
O	6.413162	7.960923	22.757327
O	1.312242	8.815646	23.181178
O	0.420725	7.235637	25.330381
O	1.514205	4.471547	25.242658
O	5.304042	7.304091	24.610355
N	5.733065	9.315673	20.584787
N	6.626674	6.902420	20.362045
C	3.157144	9.574264	21.953014
C	1.852896	9.849662	22.484003
C	5.457668	4.973332	22.219673
C	5.037582	3.911291	23.088026
C	7.191286	5.782805	20.629909
H	8.102683	5.520960	20.077418
C	3.106784	11.830299	20.963770
H	3.605603	12.589963	20.363408
C	6.311631	7.668419	24.006330
C	7.050855	9.212153	19.975164
H	7.805490	9.314707	20.766573
H	7.207362	10.003845	19.232398
C	7.543842	3.704415	21.869339
H	8.508968	3.628969	21.370238
C	1.238853	11.069375	22.276393
H	0.258374	11.268524	22.701270
C	3.759508	10.590725	21.164275
C	7.133117	2.733411	22.751517
H	7.766804	1.880298	22.977465
C	5.049826	10.400489	20.564523
H	5.478330	11.265870	20.042571
C	3.214225	3.032854	24.367907
H	3.697680	2.990408	25.356769
H	3.352209	2.061098	23.870103
C	6.726017	4.825965	21.594770
C	5.866023	2.839640	23.360170
H	5.544891	2.063109	24.049883
C	0.025688	8.977082	23.750722
H	-0.693447	9.314498	22.988485
H	0.063799	9.729506	24.554480
C	1.869474	12.072727	21.511758
H	1.369035	13.025121	21.360318
C	0.137606	4.791585	25.307969
H	-0.407649	4.002700	25.855570
H	-0.271939	4.845321	24.284156
C	7.139994	7.825354	19.361734
H	6.494349	7.767973	18.474790
H	8.165771	7.566817	19.071873
C	-0.042787	6.101239	26.038978
H	-1.109857	6.224864	26.289136
H	0.524229	6.075457	26.977398
C	-0.432567	7.645793	24.278645
H	-1.465979	7.755287	24.649100
H	-0.424396	6.906528	23.461021
C	1.739552	3.297526	24.491762
H	1.299447	3.415831	23.487377
H	1.265836	2.431262	24.985389
C	7.639969	7.772410	24.731003
H	8.213677	6.860900	24.518793
H	7.488978	7.844417	25.810932
H	8.228118	8.621379	24.371324
K	2.798516	6.595854	23.945595
O	0.977319	5.534616	22.234825
C	0.510091	5.152074	21.158954

O 0.774005 5.558354 19.995291
C 2.359573 6.895456 19.992856
C 3.500307 6.213294 19.406523
H 2.279506 6.960033 21.070756
H 1.800983 7.589548 19.372206
O 4.080589 7.463008 19.740398
H 3.884958 5.379435 20.005472
C 3.518509 5.933557 17.930754
H 2.903730 5.056026 17.699853
H 3.131108 6.793402 17.371116
H 4.544731 5.733362 17.600080
O -0.425357 4.150078 21.262124
C -0.994743 3.653682 20.061192
H -0.228887 3.229637 19.402259
H -1.696591 2.870763 20.357994
H -1.530311 4.440155 19.517966

TS1K_m06.log

SCF (M06) = -2883.93928183
E(SCF)+ZPE(0 K)= -2883.303644
H(298 K)= -2883.258562
G(298 K)= -2883.378609
Lowest Frequency = -477.3080cm-1

Co 0.01908800 -0.26064700 -0.05235800
O 0.06953200 -0.08213200 1.84215500
O 1.91880300 -0.32925700 -0.02812100
O 0.72676100 -0.40578600 4.34525100
O -0.19227100 -2.13273800 -0.01587600
O 4.45399700 -0.20554500 0.51609100
O 5.42815700 -1.23577100 2.97086100
O 3.43308800 -0.86883800 5.16493000
O 1.17871700 -2.86049300 1.62632600
N -0.08360400 -0.40758100 -1.94625400
N -1.87965100 -0.18385700 -0.09949700
C 2.70272800 -0.19532900 -1.04593900
C 4.11352000 -0.11794800 -0.79646400
C -0.87803400 -0.45725500 2.63460100
C -0.56927300 -0.65093700 4.02259300
C -2.65104000 -0.44195800 0.89222100
H -3.73033900 -0.49201500 0.70057600
C 3.22489800 0.00987800 -3.44556500
H 2.86369300 0.04549400 -4.47228000
C 0.31629800 -3.02191200 0.77102200
C -1.44329100 -0.68035500 -2.39401900
H -1.61040500 -1.76373500 -2.32967900
H -1.58671300 -0.36434500 -3.43506000
C -3.20554700 -1.06329100 3.18859000
H -4.22996100 -1.20873900 2.84860300
C 5.01230300 0.02630600 -1.83541300
H 6.07872700 0.07587600 -1.63259700
C 2.28052400 -0.13998900 -2.40121100
C -2.86843400 -1.26534900 4.50537700
H -3.61336600 -1.58028200 5.23074800
C 0.90118000 -0.30391300 -2.76187000
H 0.68172500 -0.35726100 -3.83632100
C 1.13151100 -0.49652200 5.70114300
H 1.06566000 -1.54028700 6.04699900
H 0.47893900 0.12341000 6.33455100
C -2.22867100 -0.66715200 2.24428800
C -1.53788800 -1.05428900 4.92110000
H -1.27990500 -1.21575800 5.96460200
C 5.82761800 -0.16913700 0.86776600
H 6.31175500 0.71625800 0.42626000
H 6.33210100 -1.07168100 0.48823100
C 4.56817800 0.09942800 -3.17170900
H 5.29416400 0.21399600 -3.97167600
C 4.76321600 -0.38181800 5.18289800
H 5.13908000 -0.35430300 6.22057600
H 4.77727300 0.64574700 4.77971800
C -2.38424200 0.04111500 -1.44534500
H -2.36828300 1.12000400 -1.64368000
H -3.41615900 -0.31652400 -1.54423000
C 5.64928400 -1.29682700 4.36887900
H 6.70321000 -1.05960400 4.59144500
H 5.46776600 -2.33817700 4.66093600
C 5.94449400 -0.06481100 2.36396400
H 7.01163900 0.05381700 2.61792600
H 5.39036400 0.82159700 2.71593000
C 2.54011500 0.01885100 5.80712300
H 2.60819600 1.01232800 5.33300500
H 2.80165000 0.11594300 6.87481200
C -0.32033600 -4.38035600 0.55821400
H -1.28252700 -4.39298200 1.08501400
H 0.31422500 -5.16806100 0.97110200
H -0.52138100 -4.56727500 -0.50005400
K 2.68707400 -0.88862000 2.51857900
O -0.01546300 1.74837600 -0.34494200
C 0.74401700 2.71663000 -0.56483400
O 1.76762900 3.08509400 0.05571100
C 2.19720300 2.26886000 1.72479100
C 3.56840700 2.62339700 2.08594000
H 2.01389900 1.27569500 1.34149500
H 1.39795300 2.74738400 2.28735700
O 3.41035500 1.80369400 3.18701800
H 4.30348400 2.24619300 1.33963100
C 3.84359100 4.08010500 2.38692400
H 3.84879700 4.68472100 1.47075900
H 3.07598800 4.47532400 3.06612400
H 4.82018700 4.18340400 2.87597900
O 0.43824500 3.54644300 -1.58870300
C -0.69019800 3.23834400 -2.39549900
H -1.60705200 3.21795700 -1.79659400
H -0.75356400 4.03602700 -3.13710100
H -0.56153800 2.27624100 -2.90368000

1_prop_Z_mn15.log

SCF (MN15) = -2689.401685
E(SCF)+ZPE(0 K)= -2689.361932
H(298 K)= -2689.360988
G(298 K)= -2689.474129
Lowest Frequency = 19.5462cm-1

Co 2.48415600 6.33204600 23.13475700
K 5.76222400 7.51203200 22.00931900
O 1.63878500 6.01907800 21.43839900
O 3.13010800 8.02400500 22.54848500
O 4.06294900 5.54517600 22.38365800
O 4.67495100 10.05192400 21.97518300
O 3.22550300 6.49402800 24.90084500
O 5.91380500 5.09375300 20.60955300
O 8.04941200 6.86712300 20.46772900
O 7.17809800 9.60234200 20.79937700
O 5.22970000 7.48893300 24.58434700

O	0.41816200	7.91956700	21.19487300
N	1.79741600	4.65176000	23.68118100
N	0.93498400	7.06109700	23.91131800
C	4.11598800	4.33751700	21.92049600
C	5.14970800	4.02192000	20.97464700
C	2.87092800	9.12716900	23.17533600
C	3.70489000	10.26808300	22.91366800
C	0.90490100	6.86131100	20.80893000
C	0.83042000	8.27005700	24.32068800
H	-0.07112800	8.54349900	24.87946200
C	3.43931800	1.96723700	21.80011200
H	2.76181800	1.18108000	22.12799600
C	4.30911700	7.06898700	25.29411100
C	0.69734300	4.81549100	24.62142300
H	1.11936700	5.02132700	25.61205300
H	0.07368500	3.91593300	24.66529500
C	1.60352800	10.56271500	24.73667100
H	0.76683200	10.66547700	25.42526800
C	5.32176900	2.73396900	20.49912800
H	6.12153000	2.51088000	19.79861400
C	3.24786600	3.28016200	22.29999200
C	2.45499300	11.62171300	24.50207300
H	2.31819700	12.57242600	25.00816000
C	2.16640900	3.50840000	23.22842200
H	1.61473200	2.62507700	23.56846600
C	5.52543300	11.12696000	21.61835600
H	6.16840700	11.39829500	22.46780500
H	4.93321500	12.00604100	21.32876800
C	1.79777500	9.31681700	24.08792700
C	3.51169600	11.46868700	23.57450000
H	4.17227500	12.30970800	23.38441700
C	6.95981300	4.90003700	19.67431500
H	6.57877500	4.41696000	18.76388300
H	7.74526400	4.26781900	20.11176200
C	4.46098400	1.69114200	20.91576600
H	4.61138100	0.68653400	20.53255900
C	8.07159600	9.22284900	19.77087400
H	8.77799900	10.03975800	19.55584300
H	7.51205900	9.01569500	18.84434600
C	-0.07299400	6.03930700	24.13604700
H	-0.55965800	5.81991300	23.17682400
H	-0.82533700	6.37375300	24.85884900
C	8.85738200	8.00446900	20.22222500
H	9.62035700	7.77157200	19.46373900
H	9.36247600	8.22951200	21.16628500
C	7.51057900	6.26025800	19.30955100
H	8.29021300	6.13633900	18.54259800
H	6.70205900	6.87323200	18.88252000
C	6.36149500	10.69728700	20.43519400
H	5.70632000	10.41563900	19.59620500
H	6.98231400	11.54871100	20.11707200
C	4.37018200	7.23971200	26.80575000
H	3.76256800	8.11262400	27.07380400
H	5.39843400	7.41406200	27.12919500
H	3.94971900	6.37005200	27.31677600
O	0.66972600	6.41983300	19.53668600
C	-0.16355900	7.24674000	18.73343300
H	0.27929500	8.23888900	18.60173400
H	-0.24752200	6.74610800	17.76767600
H	-1.15540200	7.35861000	19.18268700

RPO_mn15.log

SCF (MN15) = -192.825578
E(SCF)+ZPE(0 K)= -192.821175
H(298 K)= -192.820231
G(298 K)= -192.851937
Lowest Frequency = 213.9918cm-1

C	-0.64820600	-0.26895100	0.01377100
C	0.82062900	-0.26301300	0.00080400
O	0.08173600	0.95395100	-0.02312200
H	-1.15638800	-0.49862300	-0.92406600
H	1.36035600	-0.49760100	-0.91523500
H	1.35494300	-0.45995300	0.93025000
C	-1.42247300	-0.54470400	1.27378000
H	-2.34384800	0.04645900	1.30064100
H	-1.69461300	-1.60395700	1.32972300
H	-0.81703400	-0.29172900	2.15056200

TS1Co_mn15.log

SCF (MN15) = -2882.214563
E(SCF)+ZPE(0 K)= -2882.170052
H(298 K)= -2882.169107
G(298 K)= -2882.290791
Lowest Frequency = -549.8931cm-1

Co	5.11489800	7.68732600	21.31674400
O	4.51288200	5.97440100	21.93473500
O	3.70031400	8.50212100	22.30696700
O	3.65680900	4.09496900	23.55605100
O	6.42031200	7.83646400	22.71821700
O	1.28763600	8.88522500	23.21970100
O	0.44421000	7.33827800	25.40435200
O	1.46729500	4.56670800	25.27925300
O	5.30472100	7.08910300	24.53718300
N	5.73307900	9.34283800	20.64846000
N	6.49936200	6.91050200	20.29699400
C	3.14508600	9.62810400	21.98421100
C	1.83239100	9.90622800	22.49825700
C	5.33166700	4.99235700	22.16893700
C	4.91785000	3.94842000	23.06422500
C	7.06259700	5.78862300	20.56612400
H	7.96055800	5.51829800	20.00025900
C	3.09678400	11.87082600	20.95323000
H	3.60135400	12.62102900	20.34748700
C	6.32911100	7.43796000	23.94294800
C	7.04806300	9.19663900	20.03997200
H	7.79579300	9.15869400	20.84091500
H	7.26986500	10.03103400	19.36579300
C	7.44193900	3.74066700	21.86626800
H	8.40934500	3.66590300	21.37355900
C	1.21350800	11.12223300	22.26278500
H	0.22987900	11.32391900	22.67692100
C	3.75030700	10.63408200	21.18593000
C	7.03775500	2.78633600	22.77574700
H	7.68013400	1.94863500	23.02933300
C	5.06078900	10.43620500	20.61489500
H	5.51180100	11.29491500	20.10577000
C	3.11156400	3.07679300	24.37914100
H	3.63147200	3.05646100	25.34769500
H	3.22442300	2.09597300	23.89664100
C	6.60630000	4.84459500	21.55957400
C	5.76053200	2.89315200	23.37274600
H	5.44242600	2.13186300	24.07927900

C	0.01083900	9.06813000	23.80912800
H	-0.71905900	9.38939700	23.05298100
H	0.06963200	9.83547800	24.59442000
C	1.84808600	12.11774300	21.48339000
H	1.34510000	13.06410900	21.30987500
C	0.10077200	4.89932700	25.44397300
H	-0.40470400	4.12903400	26.04898300
H	-0.38564800	4.94014400	24.45712900
C	7.01413800	7.85372600	19.31522700
H	6.30826700	7.90359900	18.47716100
H	8.00163900	7.55068000	18.95046600
C	-0.00985800	6.23179100	26.16508900
H	-1.05824500	6.38820800	26.46344500
H	0.60656600	6.20438500	27.06932800
C	-0.44566700	7.74334400	24.37798100
H	-1.46023900	7.87122000	24.78690800
H	-0.47351800	6.99538100	23.57360600
C	1.63798200	3.35820300	24.56377300
H	1.14917600	3.43948200	23.58124000
H	1.18770100	2.52081200	25.12030900
C	7.67861100	7.38067100	24.64520700
H	7.54809500	7.42058500	25.72869400
H	8.33697700	8.18438200	24.30700300
H	8.15038700	6.42487100	24.38339300
K	2.72479300	6.63057700	23.90945000
O	0.77542900	5.54695800	22.23019300
C	0.58848900	4.99586800	21.13550900
O	0.97050300	5.38198000	19.98938900
C	2.37094700	6.85162600	20.06194000
C	3.51689600	6.39191000	19.28069800
H	2.40509100	6.76240000	21.13901800
H	1.69372300	7.56312800	19.60307400
O	3.97527300	7.64748500	19.76616700
H	4.05627500	5.54348000	19.71190700
C	3.41710200	6.34174200	17.77554100
H	2.88010000	5.43968800	17.46295900
H	2.88213200	7.22118300	17.40073800
H	4.41925200	6.32175200	17.33285200
O	-0.11993400	3.82549700	21.17667900
C	-0.34618400	3.14697700	19.94635900
H	0.59981600	2.86945400	19.47128000
H	-0.91373900	2.24851000	20.19476500
H	-0.92143500	3.76957500	19.25428000

TS1K_mn15.log

SCF (MN15) = -2882.192475
E(SCF)+ZPE(0 K)= -2882.147970
H(298 K)= -2882.147026
G(298 K)= -2882.268018
Lowest Frequency = -554.5235cm-1

Co	0.01974600	-0.28348300	-0.04661100
O	0.08037900	-0.02474500	1.84360400
O	1.91522200	-0.49322800	-0.05184800
O	0.76138800	-0.44264900	4.35257000
O	-0.32850300	-2.12273400	0.07442300
O	4.43589500	-0.14419200	0.51318800
O	5.43203100	-1.23532700	2.92065600
O	3.46300500	-0.93323300	5.13228400
O	1.01585700	-2.80792000	1.76290200
N	-0.09451500	-0.49452600	-1.92635300
N	-1.86007300	-0.10958500	-0.08291100
C	2.68562900	-0.20640100	-1.05805700
C	4.08562700	-0.01896600	-0.79894200
C	-0.85303600	-0.45839500	2.63724600
C	-0.53154000	-0.70287300	4.01656400
C	-2.63418600	-0.38826500	0.90311000
H	-3.71221400	-0.41351700	0.71221400
C	3.17475100	0.18612500	-3.44430000
H	2.80583700	0.25308500	-4.46577000
C	0.13141700	-2.98492600	0.92887000
C	-1.46884300	-0.75302900	-2.33525400
H	-1.68917300	-1.81030200	-2.14813800
H	-1.61434100	-0.52970200	-3.39828000
C	-3.17274300	-1.12661900	3.17945200
H	-4.19561100	-1.27135100	2.83750600
C	4.96834400	0.25531100	-1.82966600
H	6.02677300	0.38266200	-1.62293100
C	2.25171000	-0.09675500	-2.40432800
C	-2.82285300	-1.38092500	4.48815600
H	-3.55684300	-1.73885100	5.20350500
C	0.87310100	-0.34025200	-2.75701000
H	0.63939700	-0.39478400	-3.82618100
C	1.16555400	-0.57135700	5.70653700
H	1.10262400	-1.62259600	6.02221300
H	0.51504200	0.03250200	6.35440300
C	-2.20255300	-0.67422400	2.24891800
C	-1.48997600	-1.16092400	4.90524700
H	-1.22153000	-1.35758400	5.93917600
C	5.80903800	-0.05650100	0.86410100
H	6.24439600	0.87399800	0.47217200
H	6.35441100	-0.91103100	0.43879600
C	4.51203100	0.36668200	-3.16438200
H	5.22251800	0.58459400	-3.95568600
C	4.80490700	-0.48075900	5.18233900
H	5.17835900	-0.52525700	6.21812300
H	4.84581300	0.56537400	4.84114300
C	-2.34340100	0.11777900	-1.43686100
H	-2.19822200	1.17427900	-1.68254400
H	-3.40526500	-0.13599200	-1.52523800
C	5.67507800	-1.37175700	4.31140700
H	6.73239300	-1.15253400	4.52760800
H	5.48207500	-2.42054400	4.55841500
C	5.92261600	-0.02326100	2.37183300
H	6.98427400	0.10717500	2.63499300
H	5.34351900	0.82826000	2.75806100
C	2.58442400	-0.06330400	5.82283300
H	2.65408500	0.94467600	5.38720200
H	2.85706500	-0.01750400	6.88889600
C	-0.59755600	-4.31615200	0.83055400
H	-1.52952900	-4.23161700	1.40241300
H	0.01258200	-5.11007000	1.26557600
H	-0.85656600	-4.55169900	-0.20408900
K	2.68591600	-0.88496600	2.50228700
O	0.11406800	1.72357100	-0.45576100
C	0.94688600	2.61717700	-0.75102100
O	1.98533000	2.95954500	-0.12887900
C	2.28964300	2.19911800	1.58433200
C	3.57410400	2.68062900	2.10186300
H	2.23866400	1.19816600	1.18476900
H	1.39109900	2.60224600	2.04242000
O	3.32591700	1.82216900	3.16801200
H	4.43198900	2.38268200	1.46818100
C	3.66500900	4.15420800	2.45617000
H	3.72410400	4.77654100	1.55482000

H	2.78051000	4.45027300	3.03425800
H	4.55622500	4.33897700	3.06739300
O	0.71851000	3.36918100	-1.84999600
C	-0.45292200	3.11159900	-2.61717200
H	-1.35210200	3.27496100	-2.01499800
H	-0.42941500	3.81849900	-3.44676900
H	-0.45150900	2.08762000	-3.00357200

1_prop_Z_pbe.log

SCF (PBE0) = -2689.84649604
E(SCF)+ZPE(0 K)= -2689.290972
H(298 K)= -2689.250404
G(298 K)= -2689.364354
Lowest Frequency = 19.7827cm-1

Co	2.475267	6.367359	23.131130
K	5.798386	7.537114	22.053595
O	1.592046	6.140216	21.448016
O	3.165359	8.051616	22.622525
O	4.004266	5.592684	22.305737
O	4.623409	10.081830	21.947585
O	3.259546	6.416730	24.878317
O	5.917987	5.100550	20.630098
O	8.056538	6.894151	20.491097
O	7.139983	9.647076	20.772912
O	5.347527	7.227063	24.614722
O	0.490475	8.101828	21.176811
N	1.759845	4.683815	23.639302
N	0.944846	7.100952	23.954839
C	4.101558	4.366865	21.917198
C	5.170561	4.031799	21.020566
C	2.845789	9.172333	23.175760
C	3.639802	10.324209	22.858086
C	0.907463	7.011227	20.812994
C	0.820710	8.315900	24.338003
H	-0.076033	8.586288	24.902967
C	3.484957	1.980405	21.889310
H	2.819051	1.192394	22.232204
C	4.404151	6.817375	25.296446
C	0.669506	4.825471	24.588625
H	1.101912	4.940947	25.588930
H	0.013410	3.949175	24.579722
C	1.511551	10.641544	24.637206
H	0.665643	10.755939	25.310706
C	5.383354	2.729237	20.611285
H	6.206621	2.491171	19.946170
C	3.249348	3.305608	22.326141
C	2.326708	11.713253	24.351690
H	2.150138	12.684056	24.803006
C	2.146694	3.537314	23.216418
H	1.600524	2.653868	23.560152
C	5.455159	11.155777	21.550577
H	6.093159	11.468764	22.388292
H	4.850450	12.013765	21.228465
C	1.753307	9.371869	24.062224
C	3.397229	11.549950	23.448312
H	4.029126	12.401937	23.220792
C	7.020042	4.872845	19.771277
H	6.702354	4.333075	18.869301
H	7.784277	4.278788	20.290155
C	4.535342	1.690608	21.048871
H	4.718797	0.674150	20.716594
C	8.004606	9.235251	19.734518
H	8.695629	10.049204	19.465952
H	7.422170	8.986789	18.834210
C	-0.070870	6.097197	24.207159
H	-0.632233	5.936099	23.278838
H	-0.765639	6.416997	24.990479
C	8.820989	8.050045	20.204312
H	9.583788	7.823812	19.444718
H	9.331204	8.308553	21.136880
C	7.577706	6.208632	19.352601
H	8.394791	6.034171	18.636994
H	6.794521	6.786786	18.841616
C	6.290717	10.704067	20.381560
H	5.636471	10.378742	19.558399
H	6.881963	11.562611	20.029038
C	4.523153	6.782887	26.810826
H	4.045146	7.681342	27.217009
H	5.571961	6.782290	27.112650
H	4.007482	5.915436	27.228806
O	0.640124	6.550815	19.550796
C	-0.141601	7.407956	18.732921
H	0.359723	8.367188	18.572225
H	-0.257709	6.887233	17.781154
H	-1.124445	7.592231	19.177216

RPO_pbe.log

SCF (PBE0) = -192.927215339
E(SCF)+ZPE(0 K)= -192.841078
H(298 K)= -192.835751
G(298 K)= -192.867421
Lowest Frequency = 214.6233cm-1

C	-0.642918	-0.268776	0.021495
C	0.819261	-0.264049	0.000181
O	0.084415	0.959242	-0.025386
H	-1.153763	-0.500484	-0.914508
H	1.351585	-0.502895	-0.919158
H	1.362077	-0.454691	0.925365
C	-1.420747	-0.543688	1.274224
H	-2.342925	0.045966	1.293459
H	-1.694159	-1.602824	1.322070
H	-0.827725	-0.295923	2.159366

TS1Co_pbe.log

SCF (PBE0) = -2882.76293296
E(SCF)+ZPE(0 K)= -2882.119882
H(298 K)= -2882.074556
G(298 K)= -2882.196923
Lowest Frequency = -509.9148cm-1

Co	5.109134	7.650237	21.284003
O	4.553837	5.963097	21.961885
O	3.637402	8.431934	22.191131
O	3.629015	4.043098	23.458078
O	6.339200	7.946464	22.714549
O	1.354630	8.919452	23.314284
O	0.493536	7.307517	25.457044
O	1.456378	4.505058	25.232764
O	5.204780	7.326299	24.563313
N	5.694107	9.303632	20.568609
N	6.554033	6.889764	20.330048

C	3.160659	9.615069	21.994586
C	1.907889	9.943694	22.614855
C	5.335956	4.944992	22.129486
C	4.878700	3.866957	22.958029
C	7.099289	5.755840	20.575536
H	8.004385	5.490343	20.021981
C	3.191717	11.913577	21.103289
H	3.702410	12.670310	20.512979
C	6.208135	7.734310	23.976243
C	7.008882	9.192033	19.959703
H	7.759960	9.277126	20.753533
H	7.176633	9.982574	19.221392
C	7.392603	3.635156	21.756342
H	8.358870	3.549816	21.265702
C	1.351751	11.201922	22.484017
H	0.411459	11.436505	22.971753
C	3.785226	10.634014	21.226809
C	6.945431	2.644359	22.599935
H	7.552785	1.768046	22.801157
C	5.050606	10.412652	20.587129
H	5.502131	11.273269	20.085005
C	3.054701	3.016464	24.249353
H	3.582463	2.942748	25.209993
H	3.126907	2.050853	23.731769
C	6.605875	4.785924	21.513017
C	5.675462	2.762829	23.199956
H	5.326652	1.972441	23.856049
C	0.105190	9.120296	23.951631
H	-0.631369	9.510372	23.236569
H	0.215883	9.838755	24.775084
C	1.995492	12.198007	21.720221
H	1.541610	13.179331	21.629203
C	0.099343	4.871933	25.397029
H	-0.434030	4.098446	25.972254
H	-0.377635	4.958532	24.410691
C	7.083737	7.807370	19.337473
H	6.438972	7.764895	18.452371
H	8.106126	7.541670	19.050359
C	0.014646	6.176530	26.162188
H	-1.030800	6.337301	26.465377
H	0.626062	6.106910	27.067222
C	-0.389101	7.789075	24.459477
H	-1.394782	7.935442	24.881630
H	-0.453609	7.080928	23.622361
C	1.595854	3.332910	24.455111
H	1.104120	3.472868	23.482544
H	1.128668	2.477804	24.968134
C	7.485435	8.010510	24.751506
H	7.264920	8.159712	25.809904
H	8.014075	8.877225	24.348190
H	8.146647	7.142330	24.653018
K	2.715228	6.591154	23.899065
O	0.695276	5.626416	22.149492
C	0.526351	5.016591	21.087128
O	0.946395	5.313005	19.933019
C	2.401167	6.744217	19.961314
C	3.563839	6.209372	19.271847
H	2.369593	6.716679	21.037720
H	1.761072	7.435484	19.429137
O	4.021835	7.485933	19.703035
H	4.056529	5.381095	19.783985
C	3.545534	6.052254	17.775842
H	3.004139	5.140778	17.503100
H	3.055283	6.907834	17.301434
H	4.567300	5.974093	17.391330
O	-0.214248	3.866020	21.190866
C	-0.405592	3.101837	20.008865
H	0.550510	2.771194	19.592077
H	-0.997966	2.234749	20.306433
H	-0.944921	3.674140	19.248263

TS1K_pbe.log

SCF (PBE0) = -2882.096551
E(SCF)+ZPE(0 K)= -2882.052103
H(298 K)= -2882.051159
G(298 K)= -2882.173904
Lowest Frequency = -505.6722cm-1

Co	0.02738598	-0.26872547	-0.06409844
O	0.08004333	-0.09994441	1.82192366
O	1.91646681	-0.33166988	-0.03982410
O	0.71543573	-0.37654935	4.33654705
O	-0.19722853	-2.13496374	-0.02548313
O	4.44551496	-0.21167949	0.51462427
O	5.40963386	-1.26029560	2.98188793
O	3.41326304	-0.86148245	5.17645830
O	1.26319153	-2.88354829	1.52539315
N	-0.06896209	-0.42905799	-1.95127075
N	-1.86438650	-0.18487841	-0.11693665
C	2.70832699	-0.23049987	-1.05857951
C	4.11811991	-0.16447921	-0.80193467
C	-0.88344963	-0.42805737	2.62079713
C	-0.58475579	-0.59426544	4.01549915
C	-2.64908126	-0.40842694	0.87223886
H	-3.72387680	-0.44341086	0.67322589
C	3.24425157	-0.09170692	-3.45925053
H	2.89111813	-0.07530704	-4.48711608
C	0.37198319	-3.04110827	0.69530380
C	-1.42339525	-0.71438185	-2.39768076
H	-1.58673973	-1.79390262	-2.30376581
H	-1.56960742	-0.42111445	-3.44219535
C	-3.22849480	-0.95878630	3.18025676
H	-4.25356949	-1.08677022	2.84177176
C	5.02604164	-0.06255237	-1.83928982
H	6.08965830	-0.02074128	-1.63081162
C	2.29351856	-0.20097968	-2.41553699
C	-2.90096103	-1.13681841	4.50426602
H	-3.65572442	-1.41418282	5.23271267
C	0.91222572	-0.35296977	-2.77415240
H	0.68982931	-0.43063490	-3.84232924
C	1.11161143	-0.44028856	5.69681445
H	1.02493752	-1.47058132	6.06797448
H	0.47219545	0.21039167	6.30790327
C	-2.23754014	-0.61067899	2.23119240
C	-1.56717395	-0.95011376	4.92082507
H	-1.31657781	-1.09203328	5.96676284
C	5.81724870	-0.19441080	0.87766192
H	6.31607313	0.67876244	0.43653790
H	6.30952000	-1.10638473	0.51358244
C	4.58875784	-0.01837590	-3.17927602
H	5.31933912	0.06333207	-3.97723014
C	4.75422766	-0.40530023	5.20591074
H	5.12876919	-0.40752623	6.24188396
H	4.79482371	0.62366579	4.82131178
C	-2.36618161	0.02505905	-1.46333340

H	-2.34026253	1.09743226	-1.67619834
H	-3.39621118	-0.33100903	-1.56405236
C	5.62872944	-1.32751330	4.38012727
H	6.68202925	-1.10073950	4.60475563
H	5.43558188	-2.36669375	4.66409805
C	5.91851104	-0.08259388	2.37777868
H	6.98018755	0.04605439	2.63819205
H	5.34883894	0.79363933	2.71874461
C	2.53212718	0.05351749	5.79990197
H	2.62038810	1.03455770	5.31135349
H	2.78433939	0.15707490	6.86661978
C	-0.21284052	-4.41925344	0.44416942
H	0.49930734	-5.19065818	0.74186188
H	-0.49785428	-4.54681639	-0.60212755
H	-1.11689551	-4.52709785	1.05357827
K	2.68469762	-0.89644147	2.52775306
O	-0.00778127	1.72103755	-0.35543517
C	0.73612292	2.71082907	-0.54199386

O	1.76089914	3.07470566	0.08400879
C	2.23666898	2.25004223	1.70875040
C	3.57067722	2.69087106	2.11768322
H	2.13988863	1.26338525	1.28781589
H	1.39961841	2.64236316	2.27475251
O	3.41308372	1.81476941	3.18150767
H	4.35593200	2.38742854	1.40020772
C	3.74575108	4.15001129	2.48748693
H	3.74790398	4.78843608	1.59650638
H	2.93532905	4.47366411	3.15064381
H	4.69664138	4.28901452	3.01415155
O	0.40717498	3.57318113	-1.52850994
C	-0.72244479	3.26409509	-2.33587847
H	-1.63696175	3.25416856	-1.73656244
H	-0.77743603	4.05959743	-3.07919587
H	-0.59568039	2.29980900	-2.83350228

References

1. Deacy, A. C.; Moreby, E.; Phanopoulos, A.; Williams, C. K. Co(III)/Alkali-Metal(I) Heterodinuclear Catalysts for the Ring-Opening Copolymerization of CO_2 and Propylene Oxide. *J. Am. Chem. Soc.,* **2020**, 142, 19150-19160
2. Gaussian 16, Revision C.01, Frisch, M. J.; Trucks, G. W.; Schlegel, H. B.; Scuseria, G. E.; Robb, M. A.; Cheeseman, J. R.; Scalmani, G.; Barone, V.; Petersson, G. A.; Nakatsuji, H.; Li, X.; Caricato, M.; Marenich, A. V.; Bloino, J.; Janesko, B. G.; Gomperts, R.; Mennucci, B.; Hratchian, H. P.; Ortiz, J. V.; Izmaylov, A. F.; Sonnenberg, J. L.; Williams-Young, D.; Ding, F.; Lipparini, F.; Egidi, F.; Goings, J.; Peng, B.; Petrone, A.; Henderson, T.; Ranasinghe, D.; Zakrzewski, V. G.; Gao, J.; Rega, N.; Zheng, G.; Liang, W.; Hada, M.; Ehara, M.; Toyota, K.; Fukuda, R.; Hasegawa, J.; Ishida, M.; Nakajima, T.; Honda, Y.; Kitao, O.; Nakai, H.; Vreven, T.; Throssell, K.; Montgomery, J. A., Jr.; Peralta, J. E.; Ogliaro, F.; Bearpark, M. J.; Heyd, J. J.; Brothers, E. N.; Kudin, K. N.; Staroverov, V. N.; Keith, T. A.; Kobayashi, R.; Normand, J.; Raghavachari, K.; Rendell, A. P.; Burant, J. C.; Iyengar, S. S.; Tomasi, J.; Cossi, M.; Millam, J. M.; Klene, M.; Adamo, C.; Cammi, R.; Ochterski, J. W.; Martin, R. L.; Morokuma, K.; Farkas, O.; Foresman, J. B.; Fox, D. J. Gaussian, Inc., Wallingford CT, 2016.
3. Chai, J.-D.; Head-Gordon, M. Systematic Optimization of Long-Range Corrected Hybrid Density Functionals. *J. Chem. Phys.* **2007**, *128*, 084106.
4. Alipour, M.; Fallahzadeh, P. First Principles Optimally Tuned Range-Separated Density Functional Theory for Prediction of Phosphorus–Hydrogen Spin–Spin Coupling Constants. *Phys. Chem. Chem. Phys.* **2016**, *18*, 18431–18440
5. Becke, A. D. Density-Functional Thermochemistry. III. The Role of Exact Exchange. *J. Chem. Phys.* **1993**, *98*, 5648–5652
6. Lee, C.; Yang, W.; Parr, R. G. Development of the Colle-Salvetti Correlation-Energy Formula into a Functional of the Electron Density. *Phys. Rev. B* **1988**, *37*, 785–789
7. Grimme, S.; Ehrlich, S.; Goerigk, L. Effect of the Damping Function in Dispersion Corrected Density Functional Theory. *J. Comp. Chem.* **2011**, *32*, 1456–1465
8. Becke, A. D.; Johnson, E. R. A Density-Functional Model of the Dispersion Interaction. *J. Chem. Phys.* **2005**, *123*, 154101
9. Zhao, Y.; Truhlar, D. G. The M06 Suite of Density Functionals for Main Group Thermochemistry, Thermochemical Kinetics, Noncovalent Interactions, Excited States, and Transition Elements: Two New Functionals and Systematic Testing of Four M06-Class Functionals and 12 Other Functionals. *Theor. Chem. Account.* **2008**, *120*, 215–241
10. Grimme, S.; Antony, J.; Ehrilich, S.; Krieg, H. A Consistent and Accurate *Ab Initio* Parametrization of Density Functional Dispersion Correction (DFT-D) for the 94 Elements H-Pu. *J. Chem. Phys.* **2010**, *132*, 154104
11. Perdew, J. P.; Burke, K.; Ernzerhof, M. Generalized Gradient Approximation Made Simple. *Phys. Rev. Lett.* **1996**, *77*, 3865–3868
12. Adamo, C.; Barone, V. Toward Reliable Density Functional Methods Without Adjustable Parameters: The PBE0 Model. *J. Chem. Phys.* **1999**, *110*, 6158–6170
13. *NBO 6.0.* Glendening, E. D.; Badenhoop, J. K.; Reed, A. E.; Carpenter, J. E.; Bohmann, J. A.; Morales, C. M.; Landis, C. R.; Weinhold, F. Theoretical Chemistry Institute, University of Misconsin, Madison (2013)
14. AIMAll (Version 13.10.19), Keith, T. A. TK Gristmill Software, Overland Park, KS, USA (2013) (aim.tkgristmill.com)
15. Johnson, E. R.; Keinan, S.; Mori-Sánchez, P.; Contreras-García, J.; Cohen, A. J.; Yang, W. Revealing Noncovalent Interactions. *J. Am. Chem. Soc.* **2010**, *132*, 6498–6506
16. Barone, V.; Cossi, M. Quantum Calculation of Molecular Energies and Energy Gradients in Solution by a Conductor Solvent Model, *J. Phys. Chem. A.*, **1998**, 102, 1995-2001
17. Luchini, G.; Alegre-Requena, J. V.; Funes-Ardoiz, I. and Paton, R.S. Goodvibes:automated thermochemistry for heterogeneous computational chemistry data. *F1000Research*, **2020**, 9, 291
18. Foltran, S.; Cloutet, E.; Cramail, H. and Tassaing, T. In situ FTIR investigation of the solubility and swelling of model epoxides in supercritical CO_2. *J. Supercrit. Fluids*, **2012**, 63, 52-58
